# One-Pot Oxidative Amidation of Aldehydes via the Generation of Nitrile Imine Intermediates

**DOI:** 10.1021/acs.joc.4c00575

**Published:** 2024-05-23

**Authors:** Martyn C. Henry, Laura Minty, Alexander C. W. Kwok, Jessica M. L. Elwood, Adam J. Foulis, Jonathan Pettinger, Craig Jamieson

**Affiliations:** † Department of Pure and Applied Chemistry, University of Strathclyde, Glasgow G1 1XL, United Kingdom; ‡ GSK, Medicines Research Centre, Gunnels Wood Road, Stevenage SG1 2NY, United Kingdom

## Abstract

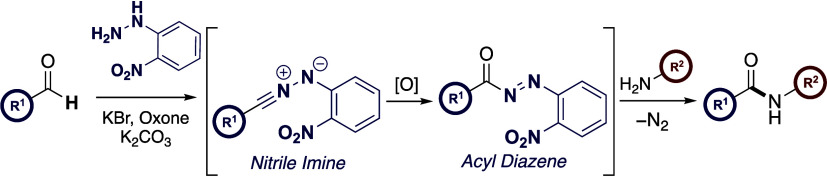

A one-pot procedure for the oxidative amidation of aldehydes via the *in situ* generation of reactive nitrile imine (NI) intermediates has been developed. Distinct from our progenitor processes, mechanistic and control experiments revealed that the NI undergoes rapid oxidation to an acyl diazene species, which then facilitates *N*-acylation of an amine. A range of substrates have been explored, including application in the synthesis of pharmaceutically relevant compounds.

## Introduction

The efficient and selective formation of amide bonds is a key deliverable in organic synthesis as this ubiquitous functional group comprises the backbone of peptides, proteins, and a range of other important biomolecules.^1^ The amide motif is prevalent across biologically active and pharmaceutically relevant small molecules, and accordingly, amidation processes account for a significant proportion (25%) of all reactions carried out in a drug discovery setting.^2^ The importance of the amide bond has directed considerable efforts toward the development of novel and efficient amidation methodologies, which has led to the creation of a large body of coupling chemistries.^3^

**Scheme 1:**
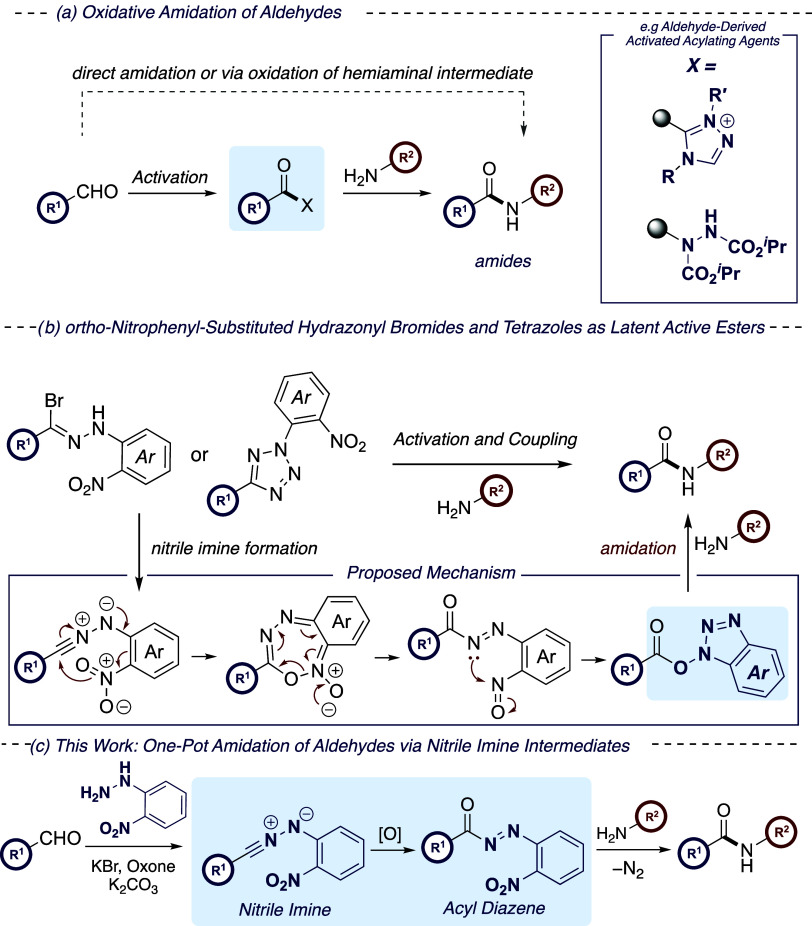
Relevant Antecedence and Proposed Study

The mainstay of amide coupling methods involves the reaction of amines with electrophilic carboxylic acid derivatives, activated via the addition of stoichiometric quantities of activating or coupling reagents.^4^ While this method is very effective and highly utilized, some limitations remain, particularly regarding recent safety concerns associated with the use of coupling reagents.^5^ Accordingly, in an effort to avoid the use of toxic, sensitizing, and atom-inefficient coupling reagents, efforts have been made toward enabling the direct coupling of aldehydes with an appropriate amine source to yield amides in a formal oxidative process (Scheme 1a).^6^ This transformation typically relies on the *in situ* activation/oxidation of the aldehyde partner via conversion into an electrophilic acylating agent such as an NHC adduct,^7^ acyl halide,^8^ acyl-imide,^9^ or active ester.^10^

Alternatively, it has been reported that unactivated aldehydes can be directly employed with unactivated amines (or preactivated chloroamines) in amidation reactions mediated by transition metal catalysis,^11^ photoredox catalysis,^12^ or electrocatalysis.^13^ A range of metal-free aldehyde oxidative amidation processes have also been developed utilizing reagents such as hypervalent iodine species, hydrogen peroxide, or oxoammonium salts.^14^ Recently, our laboratories have shown how hydrazonyl bromides and *N*-aryl-substituted tetrazoles bearing an *ortho*-nitrophenyl ring may serve as latent active esters (LAEs)^15^ via an underexploited nitrile imine (NI) rearrangement reported independently by both Huisgen and Gibson (Scheme 1b).^16^ We leveraged this transformation for the facile synthesis of amide bonds and explored the orthogonality of this process to enable the *N*-acylation of proteinogenic amino acids and peptides.

While hydrazonyl halides and tetrazoles are frequently employed as efficient NI precursors, there is a paucity of methods for the generation of NIs directly from aldehyde-derived hydrazones. In this regard, a recent disclosure by Song and Tong demonstrated how the oxidative bromination of hydrazones via the combination of potassium bromide, Oxone, and potassium carbonate resulted in the formation of NIs which were then employed in a [3 + 2] cycloaddition with various dipolarophiles in a one-pot procedure.^17^ Inspired by this work, we reasoned that aldehydes, when combined with an inexpensive 2-nitrophenylhydrazine auxiliary, could be converted directly into amides by exploiting this oxidative protocol. At the outset of the current study, it was proposed that the treatment of 2-nitrophenyl-substituted hydrazone with KBr/Oxone/K_2_CO_3_ would trigger a 1,7-electrocyclization between the nascent NI and ancillary *ortho*-nitro group resulting in the *in situ* formation of an HOBt-type active ester which could be trapped with amines to provide the desired amides. Herein, we report the development of a one-pot method for the direct oxidative amidation of aldehydes and show how our investigations revealed that this amidation method is in fact mechanistically distinct from our original design hypothesis based on the progenitor processes (Scheme 1c).

## Results and Discussion

The study commenced with the investigation of the one-pot amidation of 4-methylbenzaldehyde (**1**) using 2-nitrophenylhydrazine as an auxiliary and benzylamine as the nucleophile (Table 1). Initially, the conditions of Song and Tong were applied using KBr, Oxone, and K_2_CO_3_ in acetonitrile (Table 1, entry 1). Following full conversion to the hydrazone, the *in situ* oxidation to form the hydrazonyl bromide and subsequent hydrodehalogenation were investigated. The reaction was found to be highly sensitive to the nature of the solvent, with no conversion from the hydrazone to the amide **2a** observed when using acetonitrile, toluene, dichloromethane, and tetrahydrofuran (Table 1, entries 1–4). This was attributed to the limited solubility of KBr, Oxone, and K_2_CO_3_ in these systems. When using a mixture of acetonitrile and water (9:1), traces of desired amide **2a** (<5% NMR yield) were observed (entry 5). While the use of acetonitrile/H_2_O helped improve upon these solubility issues, the presence of water in large quantities was found to result largely in the decomposition of the highly reactive nitrile imine intermediate. The use of 1,4-dioxane and CHCl_3_ resulted in a marginal improvement in the yield, with amide **2a** formed in 10 and 13%, respectively (entries 6 and 7). DMF was found to be an effective solvent in this process, albeit in a low NMR yield (entry 8).

**Table 1:**
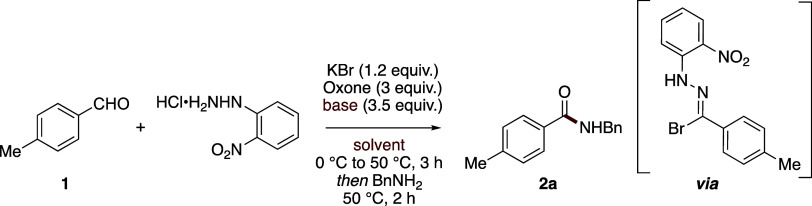
Preliminary Investigations into Solvent and Base Selection

entry	solvent	base	NMR yield (%)^a^
1	MeCN	K_2_CO_3_	0
2	toluene	K_2_CO_3_	0
3	CH_2_Cl_2_	K_2_CO_3_	0
4	THF	K_2_CO_3_	0
5	MeCN/H_2_O	K_2_CO_3_	traces
6	1,4-dioxane	K_2_CO_3_	10
7	CHCl_3_	K_2_CO_3_	13
8	DMF	K_2_CO_3_	28
9	DMF	K_3_PO_4_	27
10	DMF	Cs_2_CO_3_	25
11	DMF	Li_2_CO_3_	16
12	DMF	KO^*t*^Bu	28
13	DMF	LiOH	18
14	DMF	DBU	8
15	DMF	Et_3_N	9

a Yield determined via ^1^H NMR spectroscopy with reference to 1,3,5-trimethoxybenzene as an internal standard.

Encouraged by this result, a screen was then carried out to determine the optimal base for this transformation. Performing the reaction with K_3_PO_4_ and Cs_2_CO_3_ (entries 9 and 10) resulted in comparable assay yields to K_2_CO_3_; however, the use of Li_2_CO_3_ led to a diminished NMR yield of amide **2a** (entry 11). Potassium *tert*-butoxide was an effective base in this process, with a solution yield of **2a** of 28%, while lithium hydroxide gave **2a** in 18% yield (entries 12 and 13). Organic bases were found to perform poorly in this process with the use of DBU providing **2a** in only 8% yield, while Et_3_N gave **2a** in 9% yield (entries 14 and 15).

**Scheme 2:**
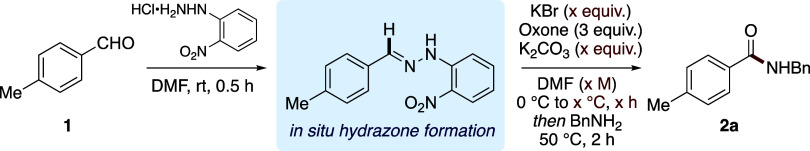
Design of Experiments Optimization Study

We next undertook a design of experiments (DoE) study as an expedient means of optimizing this aldehyde amidation process (Scheme 2).^18,19^ Accordingly, a two-level factorial, five-factor, half-fractional design was utilized assessing the effect of KBr stoichiometry (1.2–3 equiv), base stoichiometry (2–4 equiv), concentration (0.05–0.18 M), temperature (30–60 °C), and reaction time (1–3 h).

**Figure 1:**
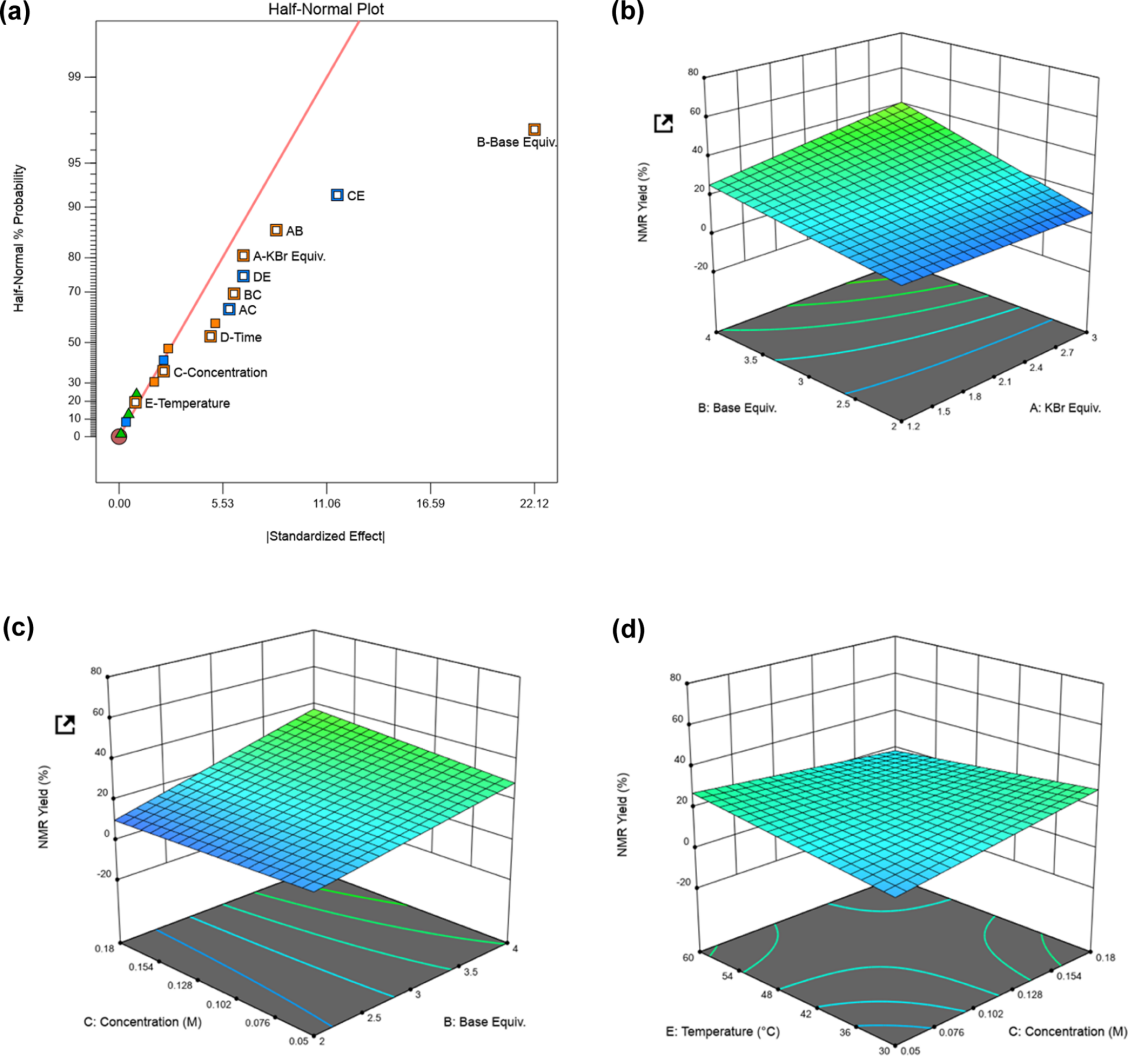
Design of experiments analysis. (a) Half-normal plot showing important factors within the two-level, half-fractional design. (b) 3-D response surface outlining the dependence of KBr and base stoichiometry on reaction conversion. (c) 3-D response surface highlighting relationship between concentration and base stoichiometry. (d) 3-D response surface showing more limited interaction between concentration and temperature.

Generally, the quantity of base was found to have a profound effect on the reaction as evidenced by the half-normal plot (Figure 1a), with increased stoichiometry significantly improving the reaction outcome. Additionally, the experiments revealed interesting two-dimensional relationships. For example, higher conversion was favored when increasing the stoichiometry of the base in combination with increased equivalents of KBr (Figure 1b), and increasing the concentration and stoichiometry of the base also resulted in elevated conversions (Figure 1c). For factors such as temperature (*C*) and concentration (*E*), there was negligible interaction observed (Figure 1d), although the half-normal plot initially suggested this as having a bearing on reaction outcome. The optimized conditions were 2 equiv of KBr, 5 equiv of K_2_CO_3_ at a concentration of 0.18 M at 50 °C for 1 h which afforded amide **2a** in 81% NMR yield which was then isolated in 61% yield. The discrepancy observed relates to more protracted purification required to separate the amide product from a hydrolysis byproduct of the reaction (*vide infra*).

**Scheme 3:**
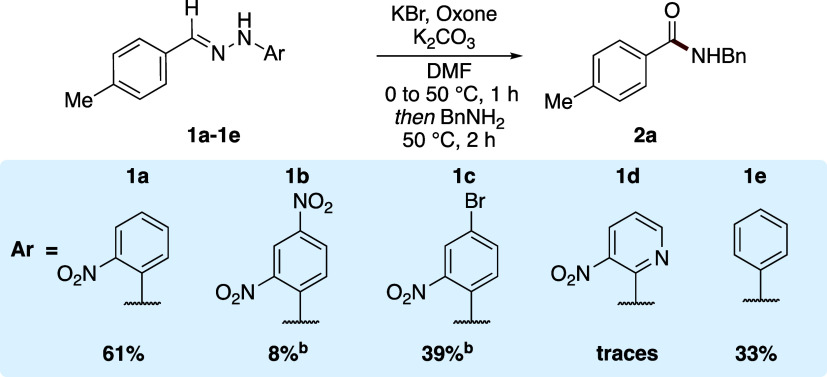
Variation of *N*-Aryl Ring and Removal of *ortho*-Nitro Substituent Yield determined via ^1^H NMR spectroscopy with reference to 1,3,5-trimethoxybenzene as an internal standard. Isolated yields unless otherwise stated.

To further enhance the conversion and tune the reactivity of the hydrazone species, the functionality of the 2-nitrophenyl aryl ring was also examined. Hydrazone compounds **1a**–**1e** were subjected to the optimized amidation reaction conditions and the yield of amide **2a** was determined (Scheme 3). Amide **2a** was isolated in 58% yield when 2-nitrophenylhydrazone **1a** was employed while 4-bromo-2-nitrophenylsubstituted hydrazone **1c** gave amide **2a** in 39% NMR yield. With 2,4-dinitrophenylhydrazone (**1b**) and pyridine derivative (**1d)**, formation of the desired amide **2a** was observed in very low yield (<10%) likely due to the poor solubility of these substrates. A negative control experiment was next performed with hydrazone substrate **1e**, without the crucial *ortho*-nitro functionality needed to facilitate the electrocyclization/cycloreversal which furnishes the active ester (*cf.*Scheme 1b). Surprisingly, this resulted in the formation of amide **2a** in 33% isolated yield. This finding implied that the emerging process is mechanistically distinct to our progenitor systems, and is not proceeding through an HOBt-type active ester via the anticipated *ortho*-nitrophenyl NI rearrangement.

**Scheme 4:**
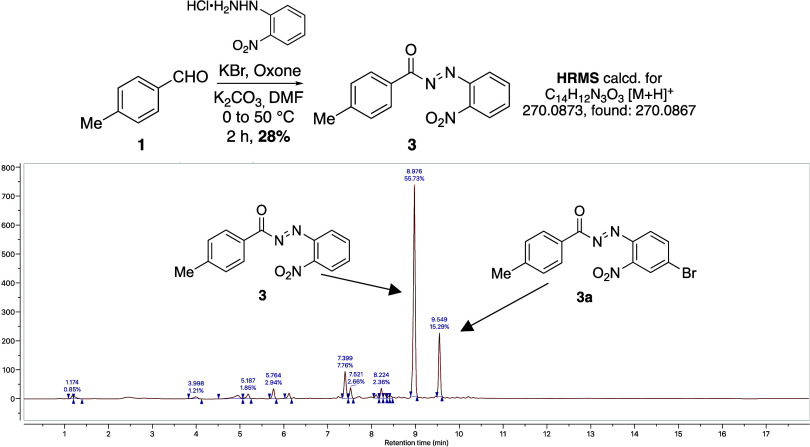
Isolation of Reactive Intermediate 3 and LC–MS Profile of Reaction Mixture

Based on the above, we then sought to further investigate the mechanism of this KBr/Oxone-mediated amidation process (Scheme 4). First, the reaction was attempted without the addition of benzylamine; however, no active ester formation was observed. Instead, acyl diazene **3** was isolated from the reaction mixture in 28% yield which was fully characterized by ^1^H/^13^C NMR spectroscopy and high-resolution mass spectrometry. Under these strongly oxidative conditions, bromination of the 4-position of the 2-nitrophenyl ring (**3a**) was also observed by liquid chromatography–mass spectrometry (LC–MS) analysis (Scheme 4).

**Scheme 5:**
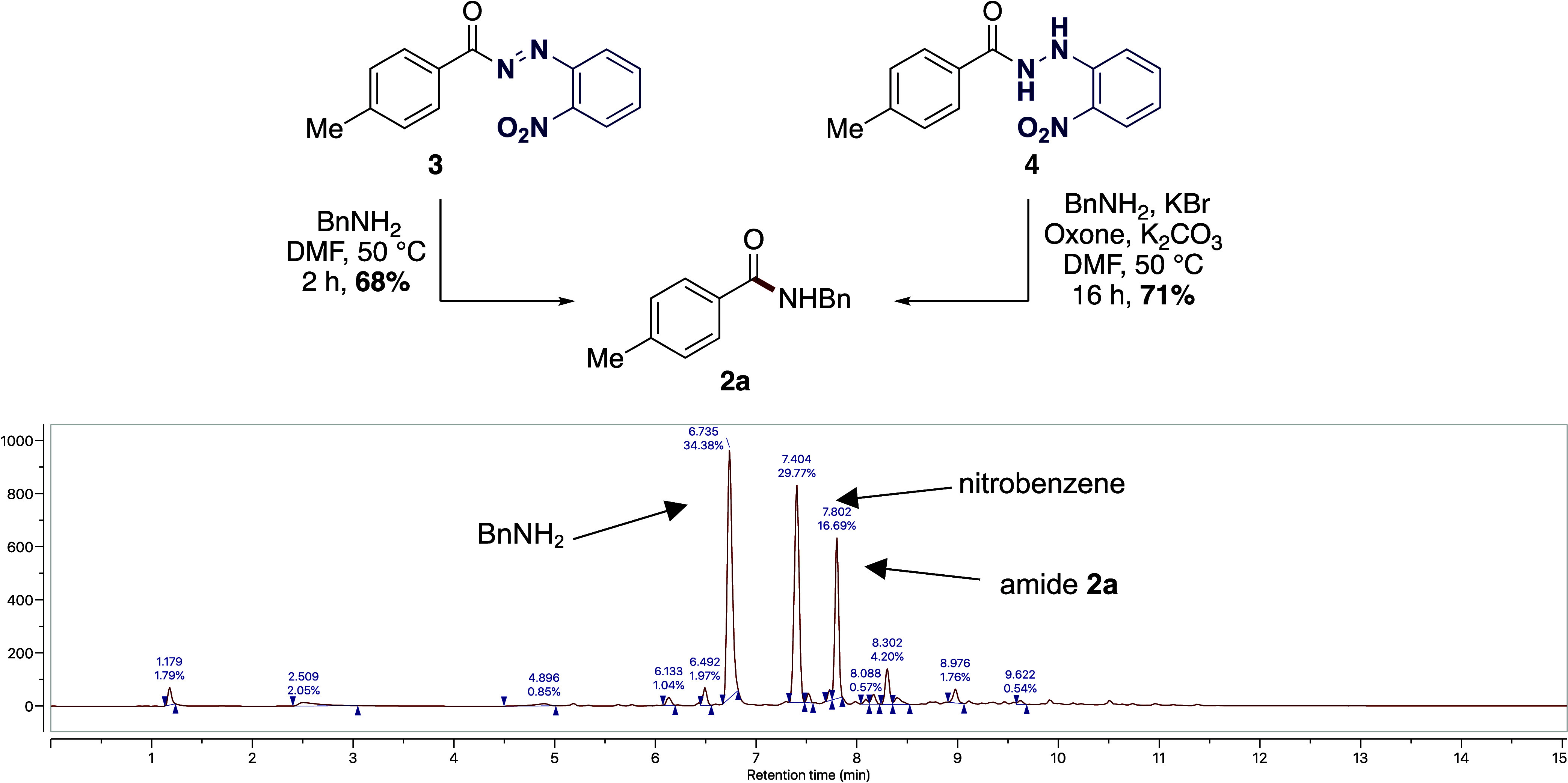
Delineation of Role of Reactive Intermediates and LC–MS Reaction Profile

Acyl diazene **3** was next reacted with benzylamine in DMF at 50 °C which afforded the desired amide **2a** in 68% isolated yield, providing further evidence that **3** is an intermediate in this process (Scheme 5). To investigate the role of hydrazide **4** as an intermediate in the process, it was independently prepared and then subjected to the oxidative conditions with KBr/Oxone which afforded amide 2a in 71% yield. Previous protocols have been reported that involve the oxidative coupling of acyl hydrazides with amines.^20^ It was reasoned that the NI was rapidly hydrolyzed to provide a hydrazide intermediate **4** which was oxidized to diazene intermediate **3***in situ*. In our manifold, acyl diazene **3** is a reactive acylating agent and reacts with the requisite amine to form the amide products, with loss of nitrogen and concomitant formation of nitrobenzene as a byproduct which was detected by LC analysis (Scheme 5), and confirmed via gas chromatography–MS (GC–MS).

**Scheme 6:**
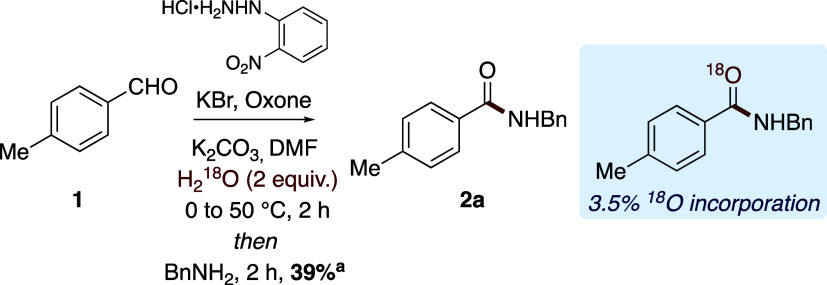
Exploration of Nitrile Imine Hydrolysis Yield determined via ^1^H NMR spectroscopy with reference to 1,3,5-trimethoxybenzene as an internal standard.

To probe the possibility of NI hydrolysis with adventitious water, the reaction mixture was doped with H_2_^18^O; however, minimal ^18^O incorporation was observed (Scheme 6). Furthermore, there is no decrease in efficiency when the reaction is performed using anhydrous solvent. However, degassing the reaction solvent with nitrogen results in a slight decrease in observed NMR yield, suggesting that molecular oxygen and Oxone are responsible for the oxidation of the NI species. In general, the HPLC profiles of the reactions shown above are representative of the process, with the desired product being obtained in reaction times between 2 and 16 h

**Scheme 7:**
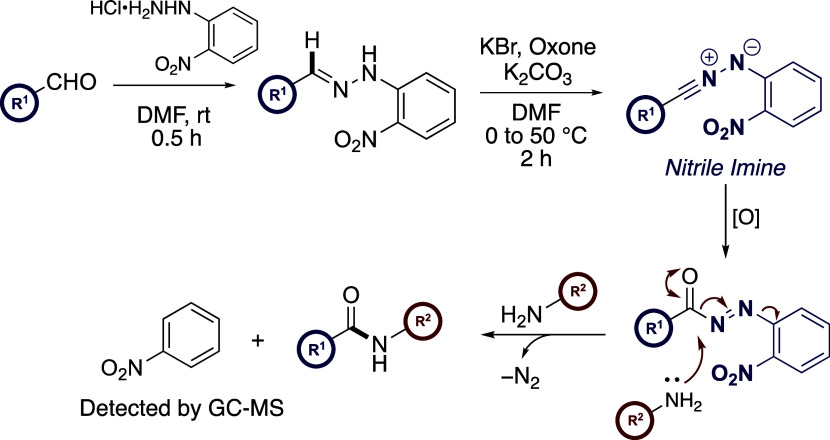
Proposed Mechanism

On the basis of these control experiments, a reaction mechanism is proposed in Scheme 7. After condensation of the aldehyde and hydrazine, the resulting hydrazone is converted to the highly reactive NI intermediate via KBr/Oxone-mediated bromination and subsequent hydrodehalogenation in the presence of potassium carbonate. Before the NI 1,3-dipole can engage in a 1,7-electrocyclization with the *ortho*-nitro motif to form an HOBt-type active ester, under the strongly oxidizing conditions of the reaction that differ from our progenitor processes which utilized only base promotion,^15^ the acyl diazene **3** is formed, likely through the intermediacy of a hydrazide species **4**. This mechanistic proposal is similar to that described previously by Müller and Waldmann, where activated acyl diazene intermediates are generated via enzyme-mediated oxidative cleavage of phenyl hydrazides with mushroom tyrosinase under an oxygen atmosphere before hydrolysis to provide carboxylic acids.^21^

**Scheme 8:**
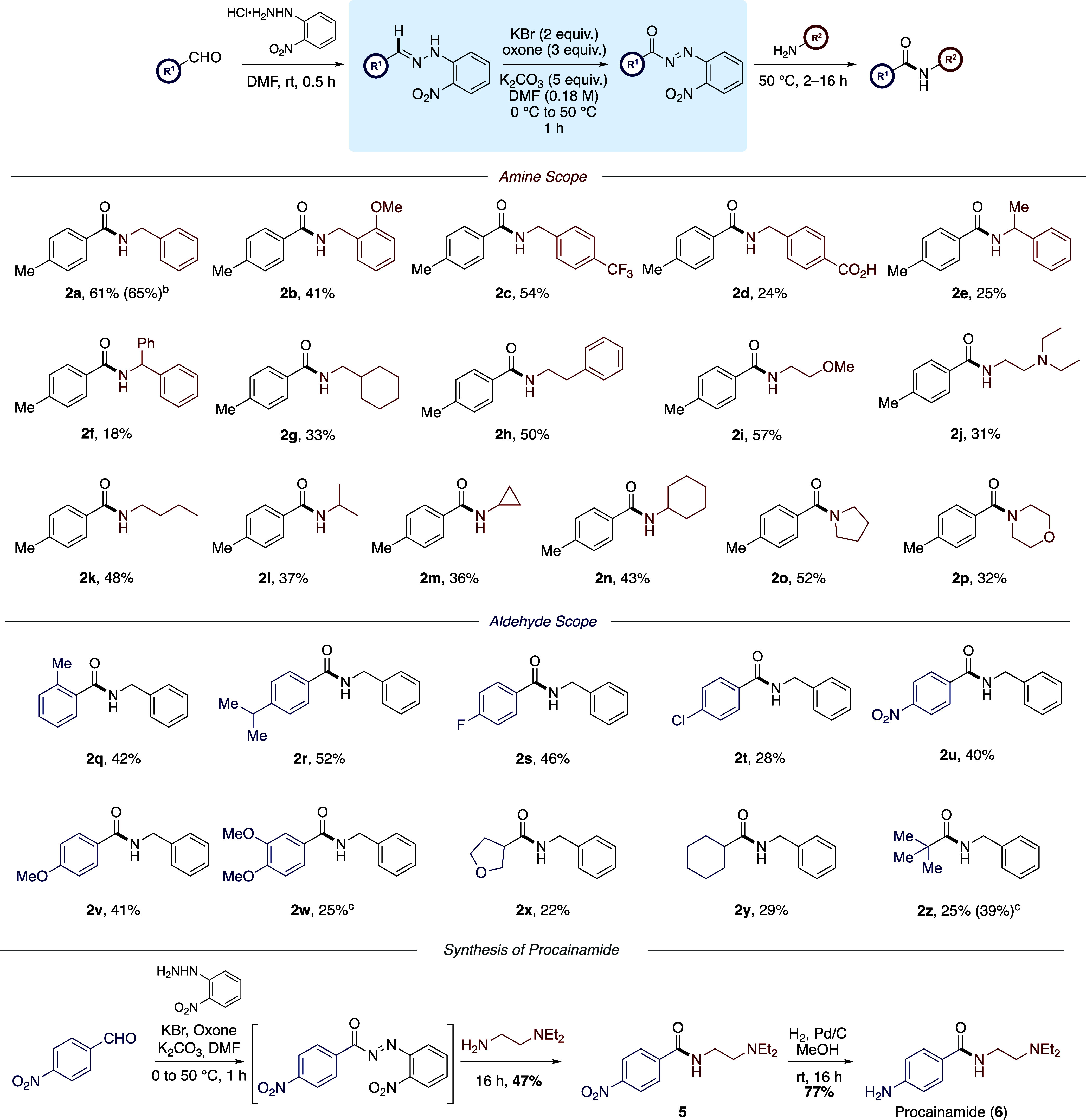
Exploration of Amine and Aldehyde Substrates Reaction performed on a 1.0 mmol scale. Amidation was performed starting from the hydrazone. Reactions were performed on a 0.5 mmol scale and isolated yields are reported.

With the optimized conditions in hand and a more complete understanding of the reaction mechanism, the scope of transformation was explored for the preparation of a range of amides (Scheme 8). Under these conditions, the amidation of 4-methylbenzaldehyde (**1**) with benzylamine was achieved in 61% yield, which was increased slightly to 65% yield of **2a** on a 1 mmol scale. Next, a range of primary benzylamine derivatives were coupled with 4-methylbenzaldehyde which afforded amides **2b** and **2c** in moderate yields (41 and 54%, respectively). Furthermore, a benzylamine derivative bearing an unprotected carboxylic acid functional group was applied under these conditions and afforded amide **2d** in 24% yield. For substrates **2e** and **2f**, lower yields (25 and 18%) were noted when using sterically hindered amines as a slower coupling reaction resulting in competitive hydrolysis of the acyl diazene intermediate. In general, and as intimated previously, where lower yields were observed with the process, the carboxylic acid resulting from hydrolysis of the incipient acyl diazene usually accounted for the mass balance. Using this one-pot, multistep approach, a range of primary amines underwent *N*-acylation to provide amides **2g**–**2l**, in moderate yields (31–57%). Cyclopropylamine and cyclohexylamine were coupled with 4-methylbenzaldehyde under these strongly oxidative conditions which provided amides **2m** and **2n** in 36 and 43% yields, respectively.

Next, more sterically hindered cyclic secondary amines were applied in the one-pot procedure. While pyrrolidine was an effective nucleophile and gave **2o** in 52% yield, morpholine was less efficient in this process and **2p** was isolated in 32% yield. Despite an extended reaction time for the amine *N*-acylation step (>16 h), the competing formation of 4-methylphenylcarboxylic acid had a negative effect on the isolated yield of amide **2p**.

With benzylamine as the *N*-nucleophile, the scope of the aldehyde component was next examined. With a methyl substituent in the *ortho*-position, amide **2q** was isolated in 42% yield while *para*-isopropyl analogue was isolated in 52% yield. Aldehydes featuring electron-withdrawing groups in the *para*-position gave the corresponding amides **2s**–**2u** in 41–58% yield. In relation to the nitrobenzene-derived substrate **2u**, the yield obtained (40%) compares very favorably with our progenitor process which used a 2,5-diaryl tetrazole precursor to furnish the amide product in 39% isolated yield.^15^ Substrates bearing electron-donating groups were generally less efficient under the strongly oxidative conditions; however, mono- and dimethoxy analogues **2v** and **2w** were isolated in 41 and 25%, respectively. In relation to α,β-unsaturated aldehydes such as cinnamaldehyde, these were not productive substrates with <10% isolated yield obtained when using benzylamine as a nucleophile. Aliphatic aldehydes were applied under these one-pot reaction conditions and gave amides **2x**, **2y**, and **2z** in comparatively lower yield (22–29%). In the case of pivaldehyde, the corresponding hydrazone could be prepared and subjected to the oxidative amidation conditions in a two-step process improving the yield of **2z** to 39%, which is then directly more aligned with the yield obtained (63%) in our earlier reaction manifold using the more elaborate tetrazole-derived precursors as input, therefore, has the benefit of using a simple aldehyde as a starting material.^15^ Procainamide (**6**), a class Ia sodium channel blocker used in the treatment of cardiac arrhythmias,^22^ was synthesized using our methodology. In a one-pot process, 4-nitrobenzaldehyde was converted to the corresponding hydrazone and, upon treatment with KBr, Oxone, and K_2_CO_3_, the acyl diazene was formed *in situ*. The intermediate acyl diazene was then treated with *N*,*N*-diethylethylenediamine which gave amide **5** in 47%. Subsequent reduction of the nitro functionality afforded procainamide (**6**) in 77% yield. The yield associated with the amidation step compares favorably with our earlier reported synthesis^15a^ of intermediate **5** which utilized a preformed diaryl tetrazole precursor to furnish the amide in 65% yield, whereas the current study employs the more readily available benzaldehyde derivative as a feedstock.

## Conclusions

In summary, we have developed a one-pot procedure that consists of five distinct steps for the synthesis of amide bonds directly from the requisite aldehyde in a formal oxidative process using readily available reagents. This involves the condensation of a simple 2-nitrophenyl-substituted hydrazine with an aldehyde, followed by KBr-Oxone-mediated bromination of the hydrazone intermediate. In contrast to our previous studies utilizing *N*-(2-nitrophenyl)-hydrazonyl bromides and tetrazoles as NI precursors, this process does not proceed *via* the anticipated formation of an HOBt-type active ester instead proceeding *via* a distinct reaction pathway. Upon the generation of the NI dipole under these strongly oxidative conditions, our observations suggest the formation of an acyl diazene species, which then serves as an activated *N*-acylating agent, allowing the synthesis of a small library of amide products and known drug procainamide (**6**).

## Experimental Section

### General Information

General experimental information and details of reaction screening, design of experiments, and mechanistic studies are provided in the Supporting Information. ***Caution!*** Oxone is an oxidizing agent which when in contact with combustible material may cause fire.

## Experimental Procedures

### General Procedure for One-Pot Amidation of Aldehydes

To a stirred solution of 2-nitrophenylhydrazine hydrochloride (0.500 mmol, 1 equiv) in *N*,*N*-dimethylformamide (3 mL) was added the appropriate aldehyde (0.550 mmol, 1.1 equiv). The resulting mixture was stirred at room temperature for 0.5 h. The reaction mixture was cooled to 0 °C, and potassium bromide (1.00 mmol, 2 equiv), Oxone (1.5 mmol, 3 equiv), and potassium carbonate (2.5 mmol, 5 equiv) were added in simultaneously. The resulting suspension was stirred at 0 °C for 5 min, warmed to 50 °C, and stirred for 2 h. The amine (2.5 mmol, 5 equiv) was added and the mixture was stirred at 50 °C for 2–16 h. The reaction mixture was cooled to room temperature and diluted with ethyl acetate (30 mL) and washed with 1 M aqueous hydrochloric acid (2 × 30 mL), 1 M aqueous sodium hydroxide (2 × 30 mL), and brine (30 mL). The organic layer was dried (MgSO_4_), filtered, and concentrated *in vacuo*. The crude residue was purified by flash column chromatography, eluting with ethyl acetate and petroleum ether, to afford the desired amide product.

#### *N*-Benzyl-4-methylbenzamide (**2a**)^23^

The reaction was performed according to the general procedure using 4-methylbenzaldehyde (65.0 μL, 0.550 mmol) and benzylamine (273 μL, 2.50 mmol). Purification by flash column chromatography (dichloromethane) afforded *N*-benzyl-4-methylbenzamide (**2a**) (68.5 mg, 61%) as an orange solid. Spectroscopic data were consistent with the literature.^23^*R*_f_ = 0.30 (petroleum ether/ethyl acetate, 4:1); ^1^H NMR (500 MHz, CDCl_3_) δ 7.69 (d, *J* = 8.2 Hz, 2H), 7.37–7.34 (m, 4H), 7.32–7.28 (m, 1H), 7.23 (d, *J* = 8.2 Hz, 2H), 6.35 (br s, 1H), 4.65 (d, *J* = 5.7 Hz, 2H), 2.39 (s, 3H); ^13^C{^1^H} NMR (126 MHz, CDCl_3_) δ 167.4, 142.1, 138.4, 131.7, 129.4, 128.9, 128.1, 127.8, 127.1, 44.3, 21.6; LCMS (ESI) *m*/*z*: [M + H]^+^ Calcd for C_15_H_16_NO 226.1; Found 226.1 at 7.21 min.

#### Synthesis of *N*-Benzyl-4-methylbenzamide (**2a**) on a 1 mmol Scale

To a stirred solution of 2-nitrophenylhydrazine hydrochloride (189 mg, 1.00 mmol) in *N*,*N*-dimethylformamide (6 mL) was added the appropriate aldehyde (130 μL, 1.10 mmol). The resulting mixture was stirred at room temperature for 0.5 h. The reaction mixture was cooled to 0 °C, and potassium bromide (238 mg, 2.00 mmol), Oxone (1.84 g, 3.00 mmol), and potassium carbonate (691 mg, 5.00 mmol) were added simultaneously. The resulting suspension was stirred at 0 °C for 5 min, warmed to 50 °C, and stirred for 2 h. Benzylamine (546 μL, 5.00 mmol) was added and the mixture was stirred at 50 °C for 2 h. The reaction mixture was cooled to room temperature and diluted with ethyl acetate (80 mL) and washed with 1 M aqueous hydrochloric acid (2 × 80 mL), 1 M aqueous sodium hydroxide (2 × 80 mL), and brine (80 mL). The organic layer was dried (MgSO_4_), filtered, and concentrated *in vacuo*. Purification by flash column chromatography (dichloromethane) afforded *N*-benzyl-4-methylbenzamide (**2a**) (146 mg, 65%) as an orange solid. Spectroscopic data are as reported above for *N*-benzyl-4-methylbenzamide (**2a**).

#### *N*-(2-Methoxybenzyl)-4-methylbenzamide (**2b**)^15^

The reaction was performed according to the general procedure using 4-methylbenzaldehyde (65.0 μL, 0.550 mmol) and 2-methoxybenzylamine (326 μL, 2.50 mmol). Purification by flash column chromatography (15% ethyl acetate in petroleum ether) followed by trituration in hexane afforded *N*-(2-methoxybenzyl)-4-methylbenzamide (**2b**) (52.3 mg, 41%) as an off-white solid. Spectroscopic data were consistent with the literature.^15^

R*f* = 0.45 (petroleum ether/ethyl acetate, 7:3); ^1^H NMR (500 MHz, CDCl_3_) δ 7.71–7.61 (m, 2H), 7.37–7.27 (m, 2H), 7.21 (d, *J* = 8.0 Hz, 2H), 6.99–6.86 (m, 2H), 6.59 (s, 1H), 4.64 (d, *J* = 5.8 Hz, 2H), 3.89 (s, 3H), 2.38 (s, 3H); ^13^C{^1^H} NMR (126 MHz, CDCl_3_) δ 157.8, 141.8, 130.2, 129.3 (2 × CH), 129.1, 127.1 (2 × CH), 126.5, 121.0, 110.5, 55.6, 40.1, 21.6; LCMS (ESI) m/z: [M + H]+ Calcd for C_16_H_18_NO_2_ 256.1; Found 256.2 at 7.96 mins.

##### 4-Methyl-*N*-(4-(trifluoromethyl)benzyl)benzamide (**2c**)

The reaction was performed according to the general procedure using 4-methylbenzaldehyde (65.0 μL, 0.550 mmol) and 4-(trifluoromethyl)benzylamine (0.356 mL, 2.50 mmol). Purification by flash column chromatography using a gradient system (10–20% ethyl acetate in petroleum ether) afforded 4-methyl-*N*-(4-(trifluoromethyl)benzyl)benzamide (**2c**) (79.6 mg, 54%) as an orange solid. *R*_f_ = 0.16 (petroleum ether/ethyl acetate, 4:1); FT-IR (neat) ν_max_ 3003, 2950, 1647, 1558, 1336, 1323, 1181, 1115, 1070, 1030, 849, 729, 685, 632 cm^–1^; ^1^H NMR (500 MHz, CDCl_3_) δ 7.70 (d, *J* = 8.2 Hz, 2H), 7.60 (d, *J* = 8.1 Hz, 2H), 7.47 (d, *J* = 8.0 Hz, 2H), 7.25 (d, *J* = 8.1 Hz, 2H), 6.45 (s, 1H), 4.70 (d, *J* = 5.9 Hz, 2H), 2.40 (s, 3H); ^13^C{^1^H} NMR (126 MHz, CDCl_3_) δ 167.6, 142.7, 142.4, 131.3, 129.9 (q, ^2^*J*_CF_ = 32.6 Hz) 129.5 (2 × CH), 128.1 (2 × CH), 127.1 (2 × CH), 125.8 (q, ^3^*J*_*CF*_ = 3.7 Hz, 2 × CH), 124.3 (q, ^1^*J*_CF_ = 273.5 Hz), 43.6, 21.6; HRMS (ESI) *m*/*z*: [M + Na]^+^ Calcd for C_16_H_15_F_3_NNaO 316.0920; Found 316.0915.

#### 4-((4-Methylbenzamido)methyl)benzoic Acid (**2d**)^24^

The reaction was performed according to the general procedure using 4-methylbenzaldehyde (65.0 μL, 0.550 mmol) and 4-(aminomethyl)benzoic acid (378 mg, 2.50 mmol). Following the standard workup, the crude residue was dissolved in dichloromethane (20 mL) and extracted with 1 M aqueous sodium hydroxide (2 × 20 mL). The combined aqueous phase was acidified to pH 1 with concentrated hydrochloric acid and then extracted with dichloromethane (3 × 30 mL). The combined organic extracts were dried (MgSO_4_), filtered, and concentrated *in vacuo*. Trituration of the residue with diethyl ether afforded 4-((4-methylbenzamido)methyl)benzoic acid (**2d**) (32.0 mg, 24%) as a beige solid. Spectroscopic data were consistent with the literature.^24^^1^H NMR (500 MHz, DMSO-*d*_6_) δ 12.94 (s, 1H), 9.04 (t, *J* = 5.9 Hz, 1H), 7.90 (d, *J* = 8.0 Hz, 2H), 7.81 (d, *J* = 8.0 Hz, 2H), 7.41 (d, *J* = 8.0 Hz, 2H), 7.28 (d, *J* = 8.0 Hz, 2H), 4.53 (d, *J* = 5.9 Hz, 2H), 2.35 (s, 3H); ^13^C{^1^H} NMR (126 MHz, DMSO-*d*_6_) δ 167.3, 166.1, 144.8, 141.2, 131.4, 129.3, 128.8, 127.3, 127.1, 42.4, 20.9; LCMS (ESI) *m*/*z*: [M + H]^+^ Calcd for C_16_H_16_NO_3_ 270.1; Found 270.4 at 6.78 min.

#### 4-Methyl-*N*-(1-phenylethyl)benzamide (**2e**)^25^

The reaction was performed according to the general procedure using 4-methylbenzaldehyde (65.0 μL, 0.550 mmol) and 1-phenylethylamine (320 μL, 2.50 mmol). Purification by flash column chromatography (15% ethyl acetate in petroleum ether) afforded 4-methyl-*N*-(1-phenylethyl)benzamide (**2e**) (30.4 mg, 25%) as an off-white solid. Spectroscopic data were consistent with the literature.^25^*R*_f_ = 0.36 (petroleum ether/ethyl acetate, 4:1); ^1^H NMR (500 MHz, CDCl_3_) δ 7.67 (d, *J* = 7.9 Hz, 2H), 7.42–7.32 (m, 2H), 7.28 (d, *J* = 7.2 Hz, 2H), 7.22 (d, *J* = 7.9 Hz, 2H), 6.28 (d, *J* = 7.6 Hz, 2H), 5.34 (quin., *J* = 7.0 Hz, 1H), 2.39 (s, 3H), 1.60 (d, *J* = 7.0 Hz, 3H); ^13^C{^1^H} NMR (126 MHz, CDCl_3_) δ 166.6, 143.4, 142.0, 131.9, 129.4, 128.9, 127.6, 127.1, 126.4, 49.3, 21.9, 21.6; LCMS: *m*/*z* calculated for C_16_H_18_NO 239.1 [M + H]^+^, Found: 240.3 at 8.05 min.

#### *N*-Benzhydryl-4-methylbenzamide (**2f**)^26^

The reaction was performed according to the general procedure using 4-methylbenzaldehyde (65.0 μL, 0.550 mmol) and 1-phenylethylamine (430 μL, 2.50 mmol). Purification by flash column chromatography (15% ethyl acetate in petroleum ether) afforded *N*-benzhydryl-4-methylbenzamide (**2f**) (27.4 mg, 18%) as a white solid. Spectroscopic data were consistent with the literature.^26^*R*_f_ = 0.47 (petroleum ether/ethyl acetate, 4:1); ^1^H NMR (500 MHz, CDCl_3_) δ 7.72 (d, *J* = 7.9 Hz, 2H), 7.39–7.26 (m, 5H), 7.24 (d, *J* = 7.9 Hz, 2H), 6.62 (br d, *J* = 7.8 Hz, 1H), 6.45 (d, *J* = 7.8 Hz, 1H), 2.40 (s, 3H); ^13^C{^1^H} NMR (126 MHz, CDCl_3_) δ 166.5, 142.3, 141.7, 131.5, 129.4, 128.9, 57.5, 21.6; LCMS (ESI): *m*/*z* calculated for C_21_H_20_NO 302.2 [M + H]^+^, Found: 302.2 at 9.86 min.

#### *N*-(Cyclohexylmethyl)-4-methylbenzamide (**2g**)^27^

The reaction was performed according to the general procedure using 4-methylbenzaldehyde (65.0 μL, 0.550 mmol) and cyclohexanemethylamine (325 μL, 2.50 mmol). The crude residue was purified by flash column chromatography (15% ethyl acetate in petroleum ether) and then washed with 1 M aq. sodium hydroxide (2 × 20 mL) to afford *N*-(cyclohexylmethyl)-4-methylbenzamide (**2g**) (37.8 mg, 33%) as an orange solid. Spectroscopic data were consistent with the literature.^27^*R*_f_ = 0.58 (petroleum ether/ethyl acetate, 7:3); ^1^H NMR (500 MHz, CDCl_3_) δ 7.66 (d, *J* = 8.1 Hz, 2H), 7.23 (d, *J* = 8.1 Hz, 2H), 6.10 (br s, 1H), 3.30 (t, *J* = 6.4 Hz, 2H), 2.39 (s, 3H), 1.82–1.71 (m, 4H), 1.71–1.63 (m, 1H), 1.59 (dd, *J* = 7.1, 3.8 Hz, 1H), 1.31–1.13 (m, 3H), 1.04–0.96 (m, 2H); ^13^C{^1^H} NMR (126 MHz, CDCl_3_) δ 167.6, 141.8, 129.3 (2 × CH), 127.0 (2 × CH), 46.3, 38.2, 31.1 (2 × CH_2_), 26.6, 26.0 (2 × CH_2_), 21.6; LCMS (ESI): *m*/*z* calculated for C_15_H_22_NO 232.2 [M + H]^+^, Found: 232.3 at 8.54 min.

#### 4-Methyl-*N*-phenethylbenzamide (**2h**)^28^

The reaction was performed according to the general procedure using 4-methylbenzaldehyde (65.0 μL, 0.550 mmol) and phenylethylamine (0.314 mL, 2.50 mmol). Purification by flash column chromatography using a gradient system (10–30% ethyl acetate in petroleum ether) afforded 4-methyl-*N*-phenethylbenzamide (**2h**) (60.3 mg, 50%) as an orange solid. Spectroscopic data were consistent with the literature.^28^*R*_f_ = 0.16 (petroleum ether/ethyl acetate, 9:1); ^1^H NMR (500 MHz, CDCl_3_) δ 7.62 (d, *J* = 8.1 Hz, 2H), 7.34 (t, *J* = 7.5 Hz, 2H), 7.30–7.23 (m, 3H), 7.21 (d, *J* = 7.9 Hz, 2H), 6.31 (t, *J* = 5.9 Hz, 1H), 3.72 (q, *J* = 6.7 Hz, 2H), 2.94 (t, *J* = 7.0 Hz, 2H), 2.40 (s, 3H); ^13^C{^1^H} NMR (126 MHz, CDCl_3_) δ 167.6, 141.9, 139.1, 131.9, 129.3 (2 × CH), 128.9 (2 × CH), 128.8 (2 × CH), 126.9 (2 × CH), 126.6, 41.2, 35.8, 21.5; LCMS (ESI) *m*/*z*: [M + H]^+^ Calcd for C_16_H_18_NO 240.3; Found 240.2 at 7.98 min.

#### *N*-(2-Methoxyethyl)-4-methylbenzamide (**2i**)

The reaction was performed according to the general procedure using 4-methylbenzaldehyde (65.0 μL, 0.550 mmol) and 2-methoxyethylamine (217 μL, 2.50 mmol). Purification by flash column chromatography using a gradient system (20–60% ethyl acetate in petroleum ether) afforded *N*-(2-methoxyethyl)-4-methylbenzamide (**2i**) (55.5 mg, 57%) as an orange solid. *R*_f_ = 0.30 (ethyl acetate); FT-IR (neat) ν_max_ 3302, 2981, 1632, 1614, 1551, 1508, 1337, 1306, 1194, 1115, 839, 748, 648, 635 cm^–1^; ^1^H NMR (500 MHz, CDCl_3_) δ 7.67 (d, *J* = 8.0 Hz, 2H), 7.21 (d, *J* = 8.0 Hz, 2H), 6.54 (s, 1H), 3.63 (q, *J* = 5.1 Hz, 2H), 3.54 (t, *J* = 5.1 Hz, 2H), 3.37 (s, 3H), 2.38 (s, 3H); ^13^C{^1^H} NMR (126 MHz, CDCl_3_) δ 167.5, 141.9, 131.8, 129.3, 127.1, 71.4, 58.9, 39.7, 21.5; HRMS (ESI) *m*/*z*: [M + H]^+^ Calcd for C_11_H_16_NO_2_ 194.1176; Found 194.1174.

#### *N*-(2-(Diethylamino)ethyl)-4-methylbenzamide (**2j**)^29^

The reaction was performed according to the general procedure using 4-methylbenzaldehyde (65.0 μL, 0.550 mmol) and *N*,*N*-diethylethylenediamine (350 μL, 2.50 mmol). The reaction mixture was diluted with ethyl acetate (30 mL) and extracted with 2 M aqueous hydrochloric acid (2 × 30 mL), and the combined aqueous phase was adjusted to pH 10 by the addition of 13 M aqueous sodium hydroxide. The aqueous phase was extracted with ethyl acetate (3 × 30 mL) and the combined organic extracts were dried (MgSO_4_), filtered, and concentrated *in vacuo*. The crude residue was filtered through an SCX-II cartridge, eluting first with methanol followed by 7 M ammonia in methanol. Purification of the resulting residue by flash column chromatography using a gradient system (0–5% methanol in dichloromethane to 5% (7 M ammonia in methanol) in dichloromethane) afforded *N*-(2-(diethylamino)ethyl)-4-methylbenzamide (**2j**) (36.4 mg, 31%) as a red oil. Spectroscopic data were consistent with the literature.^29^^1^H NMR (400 MHz, CDCl_3_) δ 7.75 (d, *J* = 8.3 Hz, 2H), 7.45 (s, 1H), 7.25–7.19 (m, 2H), 3.59 (q, *J* = 5.5 Hz, 2H), 2.83 (t, *J* = 5.5 Hz, 2H), 2.75 (q, *J* = 7.2 Hz, 4H), 2.38 (s, 3H), 1.14 (t, *J* = 7.2 Hz, 6H); ^13^C{^1^H} NMR (101 MHz, CDCl_3_) δ 167.6, 141.9, 131.5, 129.3, 127.2, 52.1, 47.6, 36.8, 21.6, 11.1; LCMS (ESI) *m*/*z*: [M + H]^+^ Calcd for C_14_H_23_N_2_O 235.2; Found 235.2 at 5.41 min.

#### *N*-Butyl-4-methylbenzamide (**2k**)^14a^

The reaction was performed according to the general procedure using 4-methylbenzaldehyde (65.0 μL, 0.550 mmol) and butylamine (247 μL, 2.50 mmol). Purification by flash column chromatography using a gradient system (10–20% ethyl acetate in petroleum ether) afforded *N*-butyl-4-methylbenzamide (**2k**) (44.1 mg, 48%) as an orange solid. Spectroscopic data were consistent with the literature.^14^*R*_f_ = 0.42 (petroleum ether/ethyl acetate, 1:1); ^1^H NMR (400 MHz, CDCl_3_) δ 7.64 (d, *J* = 8.0 Hz, 2H), 7.20 (d, *J* = 8.0 Hz, 2H), 6.16 (s, 1H), 3.43 (td, *J* = 7.3, 5.7 Hz, 2H), 2.38 (s, 3H), 1.58 (quin., *J* = 7.3 Hz, 2H), 1.40 (sext., *J* = 7.3 Hz, 2H), 0.94 (t, *J* = 7.3 Hz, 3H); ^13^C{^1^H} NMR (101 MHz, CDCl_3_) δ 167.6, 141.8, 132.1, 129.3, 126.9, 39.9, 31.9, 21.5, 20.3, 13.9; LCMS (ESI) *m*/*z*: [M + H]^+^ Calcd for C_12_H_18_NO 192.1; Found 192.2 at 7.60 min.

#### *N*-Isopropyl-4-methylbenzamide (**2l**)^30^

The reaction was performed according to the general procedure using 4-methylbenzaldehyde (65.0 μL, 0.550 mmol) and isopropylamine (214 μL, 2.50 mmol). Purification by flash column chromatography using a gradient system (10–20% ethyl acetate in petroleum ether) afforded *N*-isopropyl-4-methylbenzamide (**2l**) (33.1 mg, 37%) as an orange solid. Spectroscopic data were consistent with the literature.^30^*R*_f_ = 0.18 (petroleum ether/ethyl acetate, 4:1); ^1^H NMR (400 MHz, CDCl_3_) δ 7.64 (d, *J* = 8.2 Hz, 2H), 7.25–7.17 (m, 2H), 5.87 (br s, 1H), 4.35–4.21 (m, 1H), 2.39 (s, 3H), 1.26 (d, *J* = 6.5 Hz, 6H); ^13^C{^1^H} NMR (101 MHz, CDCl_3_) δ 166.8, 141.8, 132.3, 129.3, 126.9, 41.9, 23.1, 21.6; LCMS (ESI) *m*/*z*: [M + H]^+^ Calcd for C_11_H_16_NO 178.1; Found 178.2 at 7.04 min.

#### *N*-Cyclopropyl-4-methylbenzamide (**2m**)^31^

The reaction was performed according to the general procedure using 4-methylbenzaldehyde (65.0 μL, 0.550 mmol) and cyclopropylamine (173 μL, 2.50 mmol). Purification by flash column chromatography using a gradient system (20–40% ethyl acetate in petroleum ether) afforded *N*-cyclopropyl-4-methylbenzamide (**2m**) (31.3 mg, 36%) as an orange solid. Spectroscopic data were consistent with the literature.^31^*R*_f_ = 0.17 (petroleum ether/ethyl acetate, 1:1); ^1^H NMR (500 MHz, CDCl_3_) δ 7.63 (d, *J* = 8.2 Hz, 2H), 7.20 (d, *J* = 7.9 Hz, 2H), 6.27 (s, 1H), 2.89 (tt, *J* = 7.2, 3.6 Hz, 1H), 2.38 (s, 3H), 0.88–0.81 (m, 2H), 0.65–0.57 (m, 2H); ^13^C{^1^H} NMR (126 MHz, CDCl_3_) δ 168.9, 142.0, 131.7, 129.3, 127.0, 23.2, 21.6, 6.9; LCMS (ESI) *m*/*z*: [M + H]^+^ Calcd for C_11_H_14_NO 176.1; Found 176.2 at 6.56 min.

#### *N*-Cyclohexyl-4-methylbenzamide (**2n**)^32^

The reaction was performed according to the general procedure using 4-methylbenzaldehyde (65.0 μL, 0.550 mmol) and cyclohexylamine (286 μL, 2.50 mmol). Purification by flash column chromatography (15% ethyl acetate in petroleum ether) afforded *N*-cyclohexyl-4-methylbenzamide (**2n**) (46.9 mg, 43%) as an orange solid. Spectroscopic data were consistent with the literature.^32^*R*_f_ = 0.48 (petroleum ether/ethyl acetate, 7:3); ^1^H NMR (500 MHz, CDCl_3_) δ 7.69–7.60 (m, 2H), 7.22 (d, *J* = 7.9 Hz, 2H), 5.90 (br s, 1H), 4.02–3.93 (m, 1H), 2.39 (s, 3H), 2.06–1.99 (m, 2H), 1.75 (dt, *J* = 13.6, 4.0 Hz, 2H), 1.69–1.62 (m, 1H), 1.51–1.38 (m, 2H), 1.29–1.18 (m, 3H); ^13^C{^1^H} NMR (126 MHz, CDCl_3_) δ 166.7, 141.7, 132.4, 129.3, 126.9, 48.7, 33.4, 25.8, 25.1, 21.6; LCMS (ESI) *m*/*z*: [M + H]^+^ Calcd for C_14_H_20_NO 218.1; Found 218.2 at 8.09 min.

#### Pyrrolidin-1-yl(*p*-tolyl)methanone (**2o**)^33^

The reaction was performed according to the general procedure using 4-methylbenzaldehyde (65.0 μL, 0.550 mmol) and pyrrolidine (205 μL, 2.50 mmol). Purification by flash column chromatography using a gradient system (10–100% ethyl acetate in petroleum ether) afforded pyrrolidin-1-yl(*p*-tolyl)methanone (**2o**) (49.0 mg, 52%) as an orange solid. Spectroscopic data were consistent with the literature.^33^*R*_f_ = 0.20 (ethyl acetate); ^1^H NMR (400 MHz, CDCl_3_) δ 7.43–7.37 (m, 2H), 7.17 (dt, *J* = 7.8, 0.7 Hz, 2H), 3.62 (t, *J* = 7.0 Hz, 2H), 3.42 (t, *J* = 6.6 Hz, 2H), 2.35 (s, 4H), 1.98–1.80 (m, 4H); ^13^C{^1^H} NMR (126 MHz, CDCl_3_) δ 170.0, 140.0, 134.4, 128.9 (2 × CH), 127.3 (2 × CH), 49.8, 46.3, 26.5, 24.6, 21.5; LCMS (ESI) *m*/*z*: [M + H]^+^ Calcd for C_12_H_16_NO 190.1; Found 190.2 at 7.04 min.

#### Morpholino(*p*-tolyl)methanone (**2p**)^34^

The reaction was performed according to the general procedure using 4-methylbenzaldehyde (65.0 μL, 0.550 mmol) and morpholine (219 μL, 2.50 mmol). The crude residue was purified by flash column chromatography (15% ethyl acetate in petroleum ether) followed by further purification by filtration through a short pad of silica, eluting with 15% ethyl acetate in hexane. This afforded morpholino(*p*-tolyl)methanone (**2p**) (32.4 mg, 32%) as an orange solid. Spectroscopic data were consistent with the literature.^34^*R*_f_ = 0.18 (petroleum ether/ethyl acetate, 7:3); ^1^H NMR (500 MHz, CDCl_3_) δ 7.31 (d, *J* = 8.1 Hz, 2H), 7.21 (d, *J* = 7.8 Hz, 2H), 3.69 (s, 8H), 2.38 (s, 3H); ^13^C{^1^H} NMR (126 MHz, CDCl_3_) δ 170.8, 140.2, 132.5, 129.3 (2 × CH), 127.4 (2 × CH), 67.1, 59.7, 38.3, 31.4, 29.8; LCMS (ESI) *m*/*z*: [M + H]^+^ Calcd for C_12_H_16_NO_2_ 206.1; Found 206.2 at 6.48 min.

#### *N*-Benzyl-2-methylbenzamide (**2q**)^35^

The reaction was performed according to the general procedure using 2-methylbenzaldehyde (65.0 μL, 0.550 mmol) and benzylamine (273 μL, 2.50 mmol). Purification by flash column chromatography (dichloromethane) afforded *N*-benzyl-2-methylbenzamide (**2q**) (47.2 mg, 42%) as an orange solid. Spectroscopic data were consistent with the literature.^35^*R*_f_ = 0.53 (petroleum ether/ethyl acetate, 7:3); ^1^H NMR (500 MHz, CDCl_3_) δ 7.39–7.27 (m, 7H), 7.23–7.13 (m, 2H), 6.15 (br s, 1H), 4.60 (d, *J* = 5.8 Hz, 2H), 2.45 (s, 3H); ^13^C{^1^H} NMR (126 MHz, CDCl_3_) δ 170.0, 138.3, 136.3, 136.3, 131.1, 130.0, 128.9 (2 × CH), 127.9 (2 × CH), 127.7, 126.8, 125.8, 44.0, 19.9; LCMS (ESI) *m*/*z*: [M + H]^+^ Calcd for C_15_H_16_NO 226.1; Found 226.2 at 7.63 min.

#### *N*-Benzyl-4-isopropylbenzamide (**2r**)^36^

The reaction was performed according to the general procedure using 4-isopropylbenzaldehyde (81.5 μL, 0.550 mmol) and benzylamine (273 μL, 2.50 mmol). Purification by flash column chromatography (dichloromethane) afforded *N*-benzyl-2-methylbenzamide (**2r**) (65.9 mg, 52%) as an orange solid. Spectroscopic data were consistent with the literature.^37^*R*_f_ = 0.13 (Petroleum ether/ethyl acetate, 4:1); ^1^H NMR (400 MHz, CDCl_3_) δ 7.69–7.61 (m, 2H), 7.28–7.16 (m, 7H), 6.53 (d, *J* = 5.9 Hz, 1H), 4.54 (d, *J* = 5.7 Hz, 2H), 2.87 (p, *J* = 6.9 Hz, 1H), 1.18 (d, *J* = 6.9 Hz, 6H); ^13^C{^1^H} NMR (101 MHz, CDCl_3_) δ 167.5, 152.9, 138.5, 132.0, 128.8 (2 × CH), 127.9 (2 × CH), 127.6, 127.2 (2 × CH), 126.7 (2 × CH), 44.1, 34.2, 23.9. (2 × CH_3_); LCMS (ESI) *m*/*z*: [M + H]^+^ Calcd for C_17_H_20_NO 254.1; Found 254.2 at 8.52 min.

#### *N*-Benzyl-4-fluorobenzamide (**2s**)^37^

The reaction was performed according to the general procedure using 4-fluorobenzaldehyde (59.0 μL, 0.550 mmol) and benzylamine (273 μL, 2.50 mmol). Purification by flash column chromatography (10–20% ethyl acetate in petroleum ether) afforded *N*-benzyl-4-fluorobenzamide (**2s**) (52.9 mg, 46%) as an orange solid. Spectroscopic data were consistent with the literature.^37^*R*_f_ = 0.28 (Petroleum ether/ethyl acetate, 4:1); ^1^H NMR (400 MHz, CDCl_3_) δ 7.88–7.71 (m, 2H), 7.44–7.26 (m, 5H), 7.18–7.03 (m, 2H), 6.42 (s, 1H), 4.63 (d, *J* = 5.7 Hz, 2H); ^13^C{^1^H} NMR (101 MHz, CDCl_3_) δ 166.5, 164.9 (*J*_CF_ = 253.1 Hz), 138.2, 130.7 (*J*_CF_ = 3.6 Hz), 129.4 (*J*_CF_ = 8.7 Hz, 2 × CH), 128.9 (2 × CH), 128.0 (2 × CH), 127.8, 115.7 (*J*_CF_ = 21.9 Hz, 2 × CH), 44.3; ^19^F NMR (376 MHz, CDCl_3_) δ −108.1; LCMS (ESI) *m*/*z*: [M + H]^+^ Calcd for C_14_H_13_FNO 230.1; Found 230.2 at 7.59 min.

#### *N*-Benzyl-4-chlorobenzamide (**2t**)^10a^

The reaction was performed according to the general procedure using 4-chlorobenzaldehyde (77.3 mg, 0.550 mmol) and benzylamine (273 μL, 2.50 mmol). Purification by flash column chromatography (dichloromethane) afforded *N*-benzyl-4-chlorobenzamide (**2t**) (34.6 mg, 28%) as an orange solid. Spectroscopic data were consistent with the literature.^10^*R*_f_ = 0.41 (petroleum ether/ethyl acetate, 7:3); ^1^H NMR (500 MHz, CDCl_3_) δ 7.77–7.68 (m, 2H), 7.42–7.28 (m, 7H), 6.41 (s, 1H), 4.63 (d, *J* = 5.6 Hz, 2H); ^13^C{^1^H} NMR (126 MHz, CDCl_3_) δ 166.5, 138.0, 138.0, 132.9, 131.7, 129.0 (2 × CH), 128.5 (2 × CH), 128.1 (2 × CH), 127.9 (2 × CH), 44.4; LCMS (ESI) *m*/*z*: [M + H]^+^ Calcd for C_14_H_13_ClNO 246.1; Found 246.2 at 8.05 min.

#### *N*-Benzyl-4-nitrobenzamide (**2u**)^37^

The reaction was performed according to the general procedure using 4-nitrobenzaldehyde (83.1 mg, 0.550 mmol) and benzylamine (273 μL, 2.50 mmol). Purification by flash column chromatography using a gradient system (10–20% ethyl acetate in petroleum ether) afforded *N*-benzyl-4-nitrobenzamide (**2u**) (51.4 mg, 40%) as an orange solid. Spectroscopic data were consistent with the literature.^37^*R*_f_ = 0.47 (petroleum ether, ethyl acetate, 7:3); ^1^H NMR (400 MHz, CDCl_3_) δ 8.36–8.18 (m, 2H), 8.04–7.86 (m, 2H), 7.47–7.26 (m, 5H), 6.57 (s, 1H), 4.65 (d, *J* = 5.6 Hz, 2H); ^13^C{^1^H} NMR (101 MHz, CDCl_3_) δ 165.5, 149.8, 140.0, 137.6, 129.1 (2 × CH), 128.3 (2 × CH), 128.13 (2 × CH), 128.10, 124.0 (2 × CH), 44.6; LCMS (ESI) *m*/*z*: [M + H]^+^ Calcd for C_14_H_13_N_2_O_3_ 257.1; Found 257.2 at 7.70 min.

#### *N*-Benzyl-4-methoxybenzamide (**2v**)^38^

The reaction was performed according to the general procedure using 4-methoxybenzaldehyde (67.0 μL, 0.550 mmol) and benzylamine (273 μL, 2.50 mmol). Purification by flash column chromatography using a gradient system (10–20% ethyl acetate in petroleum ether) afforded *N*-benzyl-4-methoxybenzamide (**2v**) (49.8 mg, 41%) as an orange solid. Spectroscopic data were consistent with the literature.^38^*R*_f_ = 0.33 (petroleum ether/ethyl acetate, 7:3); ^1^H NMR (500 MHz, CDCl_3_) δ 7.74 (d, *J* = 8.8 Hz, 2H), 7.44–7.27 (m, 5H), 6.91 (d, *J* = 8.8 Hz, 2H), 6.37 (s, 1H), 4.63 (d, *J* = 5.6 Hz, 2H), 3.84 (s, 3H); ^13^C{^1^H} NMR (126 MHz, CDCl_3_) δ 167.2, 162.4, 138.4, 128.9 (2 × CH), 128.9 (2 × CH), 128.0 (2 × CH), 127.7, 126.7, 113.9 (2 × CH), 55.6, 44.3; LCMS (ESI) *m*/*z*: [M + H]^+^ Calcd for C_15_H_16_NO_2_ 242.1; Found 242.2 at 7.46 min.

#### *N*-Benzyl-3,4-dimethoxybenzamide (**2w**)^37^

To a solution of 2-nitrophenylhydrazine hydrochloride (94.5 mg, 0.500 mmol) in ethanol (2 mL) was added dimethoxybenzaldehyde (91.5 mg, 0.550 mmol) followed by a few drops of sulfuric acid. The resulting suspension was stirred under reflux for 16 h. The resulting red precipitate was filtered, dried under high vacuum, and used immediately in the amidation step. (*E*)-1-(3,4-Dimethoxybenzylidene)-2-(2-nitrophenyl)hydrazine was dissolved in *N*,*N*-dimethylformamide (3 mL) and cooled to 0 °C. Potassium bromide (119 mg, 1.00 mmol), Oxone (922 mg, 1.50 mmol), and potassium carbonate (346 mg, 2.50 mmol) were added simultaneously and the suspension was stirred at 0 °C for 10 min. The reaction mixture was warmed to 50 °C and stirred for 2 h. Benzylamine (273 μL, 2.50 mmol) was added and the resulting mixture was stirred at 50 °C for 2 h. After cooling to room temperature, the reaction mixture was diluted with ethyl acetate (20 mL) and washed with 1 M aqueous hydrochloric acid (2 × 20 mL), 1 M aqueous sodium hydroxide (2 × 20 mL), and then brine (20 mL). The organic phase was dried (MgSO_4_), filtered, and concentrated *in vacuo*. The residue was purified by flash column chromatography using a gradient system (20–50% ethyl acetate in petroleum ether) which afforded *N*-benzyl-3,4-dimethoxybenzamide (**2w**) (33.4 mg, 25%) as an orange solid. Spectroscopic data were consistent with the literature.^37^*R*_f_ = 0.15 (petroleum ether/ethyl acetate, 1:1); ^1^H NMR (400 MHz, CDCl_3_) δ 7.45 (d, *J* = 2.0 Hz, 1H), 7.37–7.26 (m, 6H), 6.83 (d, *J* = 8.4 Hz, 1H), 4.62 (d, *J* = 5.7 Hz, 2H), 3.90 (s, 6H); ^13^C{^1^H} NMR (101 MHz, CDCl_3_) δ 166.4, 151.3, 148.5, 137.9, 128.3 (2 × CH), 127.4 (2 × CH), 127.1, 126.5, 118.8, 110.2, 109.8, 55.5 (2 × CH_3_), 43.6; LCMS (ESI) *m*/*z*: [M + H]^+^ Calcd for C_16_H_18_NO_3_ 272.1; Found 272.2 at 7.15 min.

#### *N*-Benzyltetrahydrofuran-3-carboxamide (**2x**)^39^

The reaction was performed according to the general procedure using tetrahydrofuran-3-carboxaldehyde (110 μL, 0.550 mmol; 50% in H_2_O) and benzylamine (273 μL, 2.50 mmol). Purification by flash column chromatography using a gradient system (0–50% ethyl acetate/dichloromethane) afforded *N*-benzyltetrahydrofuran-3-carboxamide (**2x**) (22.3 mg, 22%) as an orange oil. Spectroscopic data were consistent with the literature.^39^*R*_f_ = 0.15 (petroleum ether/ethyl acetate, 7:3); ^1^H NMR (500 MHz, CDCl_3_) δ 7.34 (dd, *J* = 8.0, 6.5 Hz, 2H), 7.28 (dq, *J* = 8.9, 1.9 Hz, 3H), 5.90 (s, 1H), 4.45 (d, *J* = 5.7 Hz, 2H), 4.00–3.88 (m, 3H), 3.81 (td, *J* = 8.2, 6.7 Hz, 1H), 2.92 (dq, *J* = 8.4, 6.1 Hz, 1H), 2.27–2.09 (m, 2H); ^13^C{^1^H} NMR (126 MHz, CDCl_3_) δ 173.7, 138.2, 128.9 (2 × CH), 127.9 (2 × CH), 127.7, 71.1, 68.3, 45.7, 43.8, 30.6; LCMS (ESI) *m*/*z*: [M + H]^+^ Calcd for C_12_H_16_NO_2_ 206.1; Found 206.1 at 7.29 min.

#### *N*-Benzylcyclohexanecarboxamide (**2y**)^40^

The reaction was performed according to the general procedure using cyclohexanecarboxaldehyde (67.0 μL, 0.550 mmol) and benzylamine (273 μL, 2.50 mmol). Purification by flash column chromatography using a gradient system (10–20% ethyl acetate/petroleum ether) afforded *N*-benzylcyclohexanecarboxamide (**2y**) (31.6 mg, 29%) as a yellow oil. Spectroscopic data were consistent with the literature.^40^*R*_f_ = 0.24 (petroleum ether/ethyl acetate, 7:3); ^1^H NMR (400 MHz, CDCl_3_) δ 7.23–7.11 (m, 5H), 5.60 (br s, 1H), 4.31 (d, *J* = 5.6 Hz, 2H), 1.98 (tt, *J* = 11.8, 3.4 Hz, 1H), 1.82–1.61 (m, 4H), 1.55 (dd, *J* = 7.4, 3.4 Hz, 1H), 1.34 (qd, *J* = 12.2, 3.4 Hz, 2H), 1.19–1.06 (m, 3H); ^13^C{^1^H} NMR (101 MHz, CDCl_3_) δ 176.1, 138.7, 128.8 (2 × CH), 127.9 (2 × CH), 127.6, 45.7, 43.5, 29.9 (2 × CH_2_), 25.9 (3 × CH_2_); LCMS (ESI) *m*/*z*: [M + H]^+^ Calcd for C_14_H_20_NO 218.1; Found 218.2 at 8.03 min.

#### *N*-Benzylpivalamide (**2z**)^41^

The reaction was performed according to the general procedure using pivaldehyde (60.0 μL, 0.550 mmol) and benzylamine (273 μL, 2.50 mmol). Purification by flash column chromatography using a gradient system (10–20% ethyl acetate/petroleum ether) afforded *N*-benzylpivalamide (**2z**) (23.9 mg, 25%) as a yellow oil. Spectroscopic data were consistent with the literature.^41^*R*_f_ = 0.28 (petroleum ether/ethyl acetate, 7:3); ^1^H NMR (500 MHz, CDCl_3_) δ 7.30–7.12 (m, 5H), 5.92 (s, 1H), 4.35 (d, *J* = 5.6 Hz, 2H), 1.15 (s, 9H); ^13^C{^1^H} NMR (126 MHz, CDCl_3_) δ 178.4, 138.8, 128.8 (2 × CH), 127.7 (2 × CH), 127.5, 43.7, 38.8, 27.7 (3 × CH_3_); LCMS (ESI) *m*/*z*: [M + H]^+^ Calcd for C_12_H_18_NO 192.3; Found 192.3 at 7.51 min.

#### Synthesis of *N*-Benzylpivalamide (**2z**) from Hydrazone Intermediate

To a solution of 2-nitrophenylhydrazine hydrochloride (483 mg, 2.55 mmol) in ethanol (4 mL) was added pivaldehyde (252 μL, 2.32 mmol) followed by a few drops of concentrated sulfuric acid. The resulting suspension was stirred under reflux for 16 h. After cooling to room temperature, the reaction mixture was diluted with diethyl ether (20 mL) and washed with 1 M aqueous hydrochloric acid (2 × 20 mL), saturated aqueous sodium bicarbonate (20 mL), and brine (20 mL). The organic phase was dried (MgSO_4_), filtered, and concentrated in vacuo to afford (*E*)-1-(2,2-dimethylpropylidene)-2-(2-nitrophenyl)hydrazine (349 mg, 68%) as an orange solid which was used immediately in the next step without purification. (*E*)-1-(2,2-Dimethylpropylidene)-2-(2-nitrophenyl)hydrazine (111 mg, 0.500 mmol) was dissolved in *N*,*N*-dimethylformamide (3 mL) and cooled to 0 °C. Potassium bromide (119 mg, 1.00 mmol), Oxone (922 mg, 1.50 mmol), and potassium carbonate (346 mg, 2.50 mmol) were added simultaneously and the suspension was stirred at 0 °C for 10 min. The reaction mixture was warmed to 50 °C and stirred for 2 h. Benzylamine (273 μL, 2.50 mmol) was added and the resulting mixture was stirred at 50 °C for 2 h. After cooling to room temperature, the reaction mixture was diluted with ethyl acetate (20 mL) and washed with 1 M aqueous hydrochloric acid (2 × 20 mL), aqueous saturated sodium carbonate solution (2 × 20 mL), and then brine (20 mL). The organic phase was dried (MgSO_4_), filtered, and concentrated *in vacuo*. The residue was purified by flash column chromatography using a gradient system (5–20% ethyl acetate in hexane) which afforded *N*-benzylpivalamide (**2z**) (37.5 mg, 39%) as a yellow oil. Spectroscopic data are as reported above for *N*-benzylpivalamide (**2z**).

#### *N*-(2-(Diethylamino)ethyl)-4-nitrobenzamide (**5**)^29^

The reaction was performed according to the general procedure using 4-nitrobenzaldehyde (83.0 mg, 0.550 mmol) and *N*,*N*-diethylethylenediamine (350 μL, 2.50 mmol). The amidation step was carried out at 50 °C for 16 h. The reaction mixture was filtered over a Celite plug, washed with ethyl acetate, and then concentrated *in vacuo*. The residue was dissolved in methanol and filtered through and SCX-II cartridge, eluting first with methanol then 7 M ammonia in methanol. Purification of the resulting residue by flash chromatography using a gradient system (5–10% methanol in dichloromethane) afforded *N*-(2-(diethylamino)ethyl)-4-nitrobenzamide (**5**) (54.9 mg, 47%) as a brown oil. Spectroscopic data were consistent with the literature.^29^*R*_f_ = 0.33 (dichloromethane/methanol, 9:1); ^1^H NMR (500 MHz, CDCl_3_) δ 8.27 (d, *J* = 8.8 Hz, 2H), 7.97 (d, *J* = 8.8 Hz, 2H), 7.44 (s, 1H), 3.54 (q, *J* = 5.4 Hz, 2H), 2.74 (t, *J* = 5.8 Hz, 2H), 2.65 (q, *J* = 7.1 Hz, 4H), 1.08 (t, *J* = 7.1 Hz, 6H); ^13^C{^1^H} NMR (126 MHz, CDCl_3_) δ 165.3, 149.7, 140.2, 128.3, 123.9, 51.4, 47.0, 37.3, 11.6; LCMS (ESI) *m*/*z*: [M + H]^+^ Calcd for C_13_H_20_N_3_O_3_ 266.1; Found 266.2 at 5.27 min.

#### 4-Amino-*N*-(2-(diethylamino)ethyl)benzamide, Procainamide (**6**)^29^

*N*-(2-(Diethylamino)ethyl)-4-nitrobenzamide (**5**) (60.0 mg, 0.226 mmol) was dissolved in methanol (4 mL), and palladium on carbon (12 mg; 10 wt % Pd) was added. The reaction mixture was sparged with hydrogen for 0.5 h and then stirred under a hydrogen atmosphere for 16 h. The reaction mixture was filtered over a Celite plug, washed with methanol, and then concentrated *in vacuo*. This afforded 4-amino-*N*-(2-(diethylamino)ethyl)benzamide (**6**) (41.1 mg, 77%) as a colorless oil. Spectroscopic data were consistent with the literature.^29^*R*_f_ = 0.28 (7 M ammonia in methanol/dichloromethane, 9:1); ^1^H NMR (500 MHz, CDCl_3_) δ 7.59 (d, *J* = 8.3 Hz, 2H), 6.78 (s, 1H), 6.70–6.58 (m, 2H), 4.00 (s, 2H), 3.52–3.36 (m, 2H), 2.61 (t, *J* = 6.0 Hz, 2H), 2.54 (q, *J* = 7.1 Hz, 4H), 1.01 (t, *J* = 7.1 Hz, 6H); ^13^C{^1^H} NMR (126 MHz, CDCl_3_) δ 167.2, 149.6, 128.7 (2 × CH), 124.4, 114.2 (2 × CH), 51.6, 46.9 (2 × CH_2_), 37.3, 12.1 (2 × CH_3_); LCMS (ESI) *m*/*z*: [M + H]^+^ Calcd for C_13_H_22_N_3_O 235.2; Found 235.2 at 3.74 min.

## Data Availability

The data underlying this study are available in the published article and its Supporting Information.

## Supporting Information Available

Experimental procedures for reaction screening; optimization and mechanistic studies; characterization data; and NMR spectra of all compounds (PDF)
