# Supplementary material for: One-Pot Oxidative Amidation of Aldehydes via the Generation of Nitrile Imine Intermediates
Source: J Org Chem. 2024 May 23;89(11):7913–26. doi: 10.1021/acs.joc.4c00575 (PMC11165588; doi:10.1021/acs.joc.4c00575)

Supporting Information for:

One-Pot Oxidative Amidation of Aldehydes *via* the Generation of Nitrile Imine Intermediates

Martyn C. Henry,[a] Laura Minty,[a] Alexander C. W. Kwok,[a] Jessica M. L. Elwood,[a] Adam J. Foulis,[a] Jonathan Pettinger,[b] and Craig Jamieson[a]*

[a]*Department of Pure and Applied Chemistry, University of Strathclyde, Glasgow G1 1XL, United Kingdom. Email: craig.jamieson@strath.ac.uk*

[b]*GSK, Medicines Research Centre, Gunnels Wood Road, Stevenage, SG1 2NY, United Kingdom.*

Table of Contents

1. General Experimental S2
2. Optimization of Reaction Conditions S4
3. Design of Experiments Details S10
4. Experimental Procedures for Reaction Mechanism Elucidation S14
5. ^{1}H and ^{13}C NMR Spectra for all Compounds S27

1. General Experimental

All reagents and starting materials were obtained from commercial sources and used as received without further purification, unless otherwise stated. Oxone®, monopersulfate ($2KHSO_5$•$KHSO_4$•K_2SO_4) was used in this study. Acetone, dichloromethane, *N*,*N*-dimethylformamide (Rathburn, peptide grade), ethyl acetate, hexane, methanol, petroleum ether 40–60 °C, and tetrahydrofuran were used as obtained from suppliers without further purification. All dry solvents were purified using a PureSolv SPS-400-5 Solvent Purification System.

All reactions were performed using round-bottom flasks or microwave vials of appropriate volume. Reactions were carried out at elevated temperatures using a temperature regulated hotplate/stirrer and DrySyn block with a contact thermometer. Room temperature generally refers to ~ 20 °C. Reactions requiring a reduced temperature were performed using an ice bath (0 °C) and a temperature probe unless otherwise stated. Brine refers to a saturated aqueous solution of sodium chloride.

Reactions were monitored by thin layer chromatography (TLC) using Merck silica gel 60 covered aluminium backed plated F254. TLC plates were visualised under UV light and staining using potassium permanganate solution, vanillin or ninhydrin. Flash column chromatography was performed with Fluorochem silica gel 60A (40−63 μm).

Infrared spectra were recorded on a Thermo Scientific Nicolet™ iS50 FTIR spectrometer; wavenumbers are indicated in cm^{-1}. ^{1}H, ^{19}F and ^{13}C NMR spectra were recorded on a Bruker 500 NMR spectrometer at 500, 471 and 126 MHz, respectively or on a Bruker AV3 400 NMR spectrometer at 400, 376 or 101 MHz using the deuterated solvent as the internal deuterium lock. Chemical shifts (δ) are reported in ppm relative to the residual protic solvent where δ ($CDCl_3$) = 7.26 ppm (^{1}H) and δ ($CDCl_3$) = 77.16 ppm (^{13}C); δ (DMSO-d_6) = 2.50 ppm (^{1}H) and δ (DMSO-d_6) = 39.5 ppm (^{13}C); ^{1}H signals are described as singlets (s), doublets (d), triplets (t), quartets (q), multiplets (m), broad (br), app (apparent) or a combination of these and coupling constants are measured in Hz.

Low-resolution mass spectra were obtained using an Agilent Technologies 1200 series instrument with a 6130 single quadrupole LC/MS using a poroshell EC-C18 column. Analysis was performed using a gradient method, eluting with 5–95% acetonitrile (containing 5nM ammonium acetate)/water (containing 5nM ammonium acetate) over 18 minutes at a flow rate of 1 mL/min, with UV monitoring at 254 or 214 nm. GCMS data were recorded on an Agilent 7890A GC system coupled to a 5975C inert XL EI/CI MSD triple-axis mass detector. The column temperature was 320 °C, and the carrier gas was helium with a flow rate of 1 mL/min. High-resolution mass spectra were recorded using a Thermo Scientific Orbitrap Exactive Plus equipped with a Vanquish LC.

General Optimization Procedure

To a stirred solution of 2-nitrophenylhydrazine hydrochloride (56.9 mg, 0.300 mmol, 1 equiv.) in the requisite solvent (5 mL) was added 4-methylbenzaldehyde (38.9 µL, 0.330 mmol, 1.1 equiv.). The resulting suspension was stirred at room temperature for 1 h, cooled to 0 °C and the halogen salt (1.2 equiv.), oxidant (3 equiv.) and base (3.5 equiv.) were added. After stirring at 0 °C for 10 minutes, the reaction mixture was warmed to 50 °C and stirred for 3 h. Benzylamine (82.0 µL, 0.75 mmol, 2.5 equiv.) was added and the mixture was stirred at 50 °C for 2 h. The reaction mixture was diluted with ethyl acetate (20 mL) and washed with 1 M aq. hydrochloric acid (2 × 20 mL), 1 M aq. sodium hydroxide (2 × 20 mL) and then brine (20 mL). The organic layer was dried ($MgSO_4$), filtered, spiked with 1,3,5-trimethoxybenzene (50.5 mg, 0.300 mmol) and concentrated *in vacuo*. The residue was dissolved in $CDCl_3$ (0.7 mL) and analysed *via* ^{1}H NMR spectroscopy.

In cases where amide **2a** was isolated during the optimzation process, purification of the crude residue by flash column chromatography (dichloromethane) afforded *N*-benzyl-4-methylbenzamide (**2a**) as an orange solid. Spectroscopic data were consistent with the literature.[1] R*f* = 0.30 (petroleum ether/ethyl acetate, 4:1); ^{1}H NMR (500 MHz, $CDCl_3$) δ 7.69 (d, *J* = 8.2 Hz, 2H), 7.37–7.34 (m, 4H), 7.32–7.28 (m, 1H), 7.23 (d, *J* = 8.2 Hz, 2H), 6.35 (br s, 1H), 4.65 (d, *J* = 5.7 Hz, 2H), 2.39 (s, 3H); ^{13}C{^{1}H} NMR (126 MHz, $CDCl_3$) δ 167.4, 142.1, 138.4, 131.7, 129.4, 128.9, 128.1, 127.8, 127.1, 44.3, 21.6; LCMS (ESI) m/z: $[M + H]^+$ Calcd. for $C_{15}H_{16}NO$ 226.1; Found 226.1 at 7.21 mins.

Solvent Screen[a]

Table S1: Selection of optimal solvent

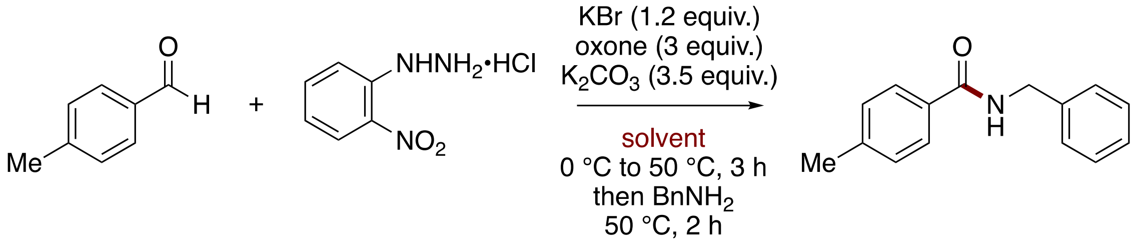

Entry	Solvent	Yield (%)[b]
1	MeCN	n/a
2	$MeCN/H_2O$ (9:1)	traces
3	CH_2Cl_2	n/a
4	toluene	n/a
5	THF	n/a
6	1,4-dioxane	10%
7	$CHCl_3$	13%
8	acetone	traces
9	EtOAc	traces
10	*t*BuOH	traces
11	DMSO	traces
12	DMF	28

[a]Reactions performed on a 0.3 mmol scale. [b]Yield determined by ^1H NMR spectroscopy with reference to 1,3,5-trimethoxybenzene.

Base Screen[a]

Table S2: Selection of the optimal base

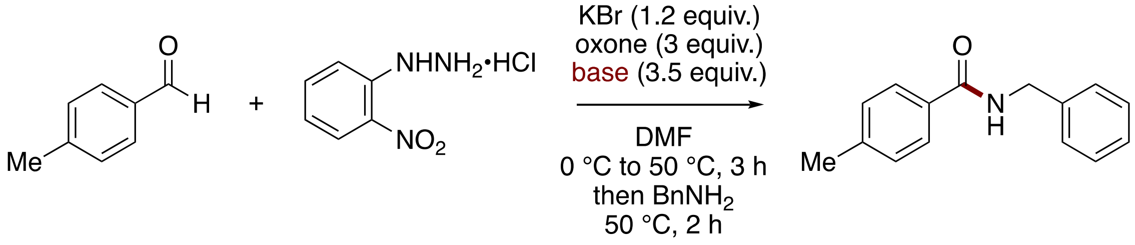

Entry	Base	Yield (%)[b]
1	K_2CO_3	28
2	K_3PO_4	27
3	Cs_2CO_3	25
4	Li_2CO_3	16
5	KO^tBu	28
6	LiOH	18
7	DBU	8
8	Et_3N	traces
9	DIPEA	traces

[a]Reactions performed on a 0.3 mmol scale. [b]Yield determined by ^{1}H NMR spectroscopy with reference to 1,3,5-trimethoxybenzene.

Oxidant Screen[a]

Table S3: Selection of the optimal oxidant

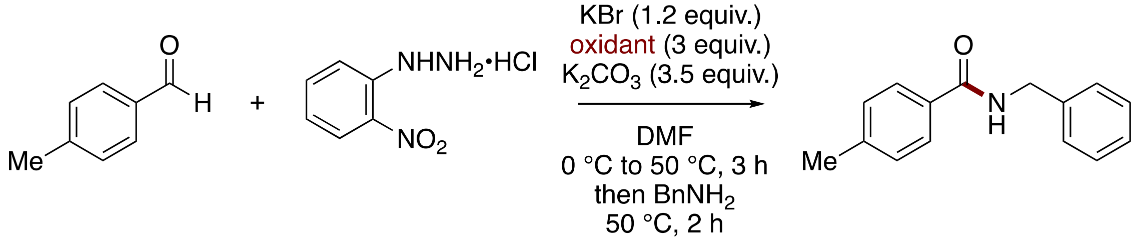

Entry	Oxidant	Yield (%)[b]
1	Oxone	28
2	NBS	18
3	NCS	9
4	NIS	9
5	mCPBA	4
6	$PhI(OAc)_2$	4
7	$(NH_4)_2S_2O_8$	traces

[a]Reactions performed on a 0.3 mmol scale. [b]Yield determined by ^1H NMR spectroscopy with reference to 1,3,5-trimethoxybenzene.

Halogen Salt Screen[a]

Table S4: Selection of optimal halogen salt

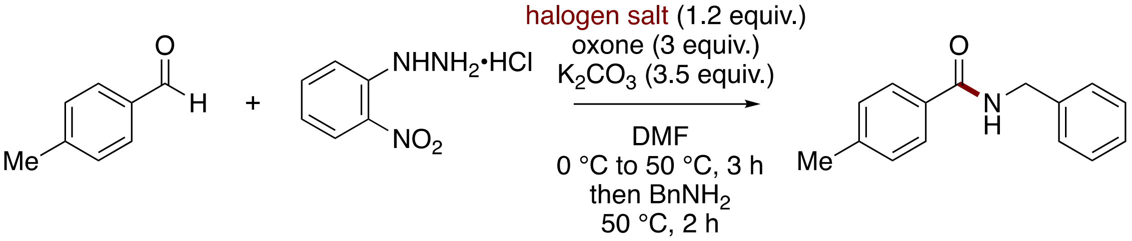

Entry	Halogen Salt	Yield (%)[b]
1	KBr	28
2	KCl	35
3	NaCl	5
4	NaBr	32
5	LiCl	19
6	LiBr	23

[a]Reactions performed on a 0.3 mmol scale. [b]Yield determined by ^{1}H NMR spectroscopy with reference to 1,3,5-trimethoxybenzene. KBr and KCl were viewed as being equally effective within the limits of error of the NMR assay.

Reaction Time (Oxidation Step) Study[a]

Table S5: The effect of the reaction time of the oxidation step upon the isolated yield of ***2a***

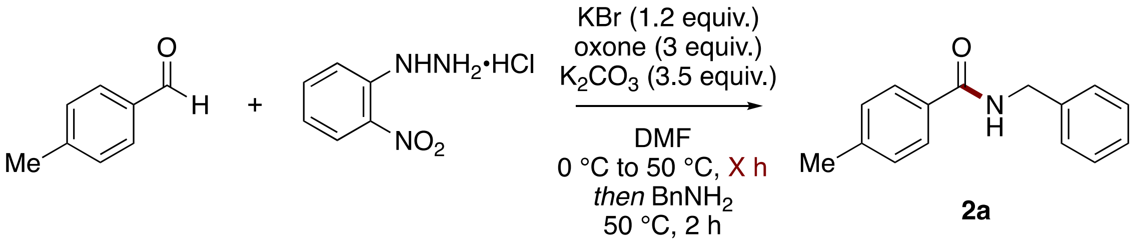

2a

Entry	Reaction Time	Yield (%)[b]
1	0.5 h	16
2	1	67
3	2	42
4	3	32
5	4	36
6	5	38
7	19	39

[a]Reactions performed on a 0.3 mmol scale. [b]Isolated yields

Design of Experiments (DoE) Study

Table S6: Data obtained in the DoE Study

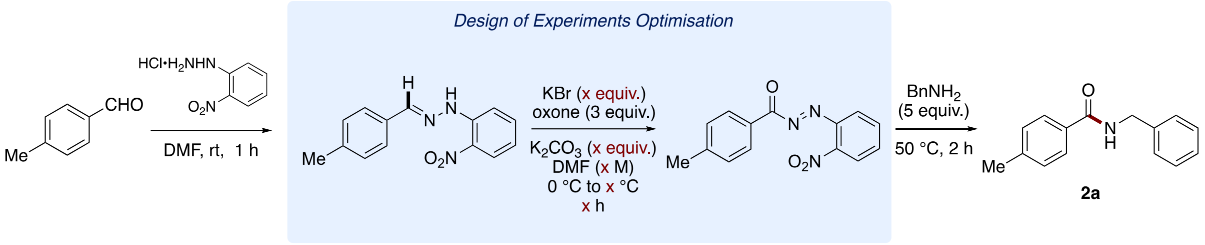

Entry	KBr Equiv.	Base Equiv.	Concentration (M)	Temperature (°C)	Time (h)	NMR Yield (%)[a]
1	1.2	2	0.05	60	1	20
2	3	2	0.05	30	1	7
3	1.2	4	0.05	30	1	6
4	3	4	0.05	60	1	48
5	1.2	2	0.18	30	1	15
6	3	2	0.18	60	1	6
7	1.2	4	0.18	60	1	23
8	3	4	0.18	30	1	39
9	1.2	2	0.05	30	3	10
10	3	2	0.05	60	3	18
11	1.2	4	0.05	60	3	26
12	3	4	0.05	30	3	39

13	1.2	2	0.18	60	3	6
14	3	2	0.18	30	3	13
15	1.2	4	0.18	30	3	51
16	3	4	0.18	60	3	40
17	2.1	3	0.115	45	2	54
18	2.1	3	0.115	45	2	57
19	2.1	3	0.115	45	2	63
20	2.1	3	0.115	45	2	49

[a]Yield determined by ^{1}H NMR spectroscopy with reference to 1,3,5-trimethoxybenzene.

Pareto t-Value Chart of Standardized Effects

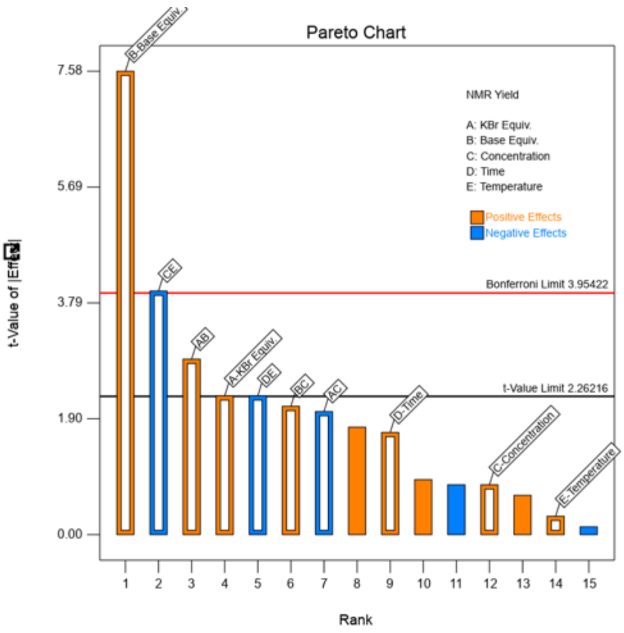

Figure 1: Pareto chart showing the standardized effects for the oxidative amidation of aldehydes (response = ^{1}H NMR spectroscopy yield with reference to 1,3,5-trimethoxybenzene).

Half-Normal Plot

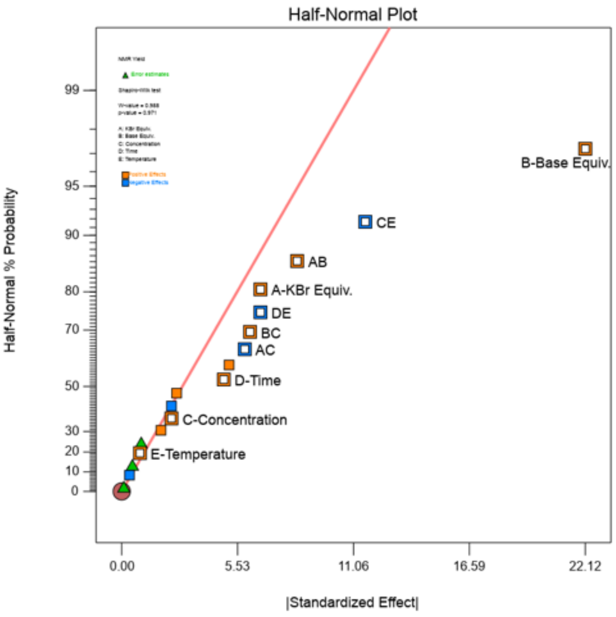

Figure S2: Half-Normal Plot of the Design of Experiments Study showing the significant effects of the base stoichiometry (B) and the relationship between concentration (C) and temperature (E).

Multi-Factor Interaction Plots

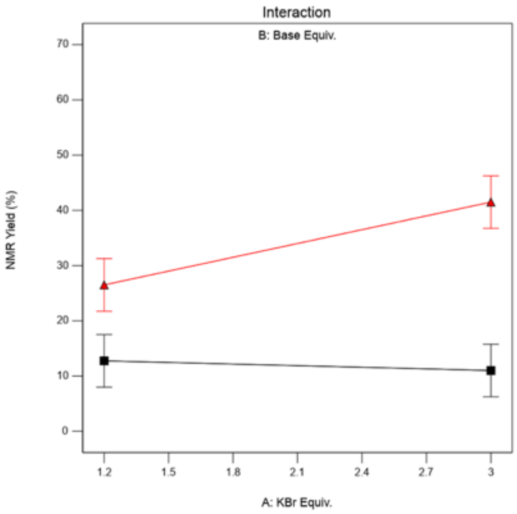

Figure S3: Interaction between KBr and base stoichiometry

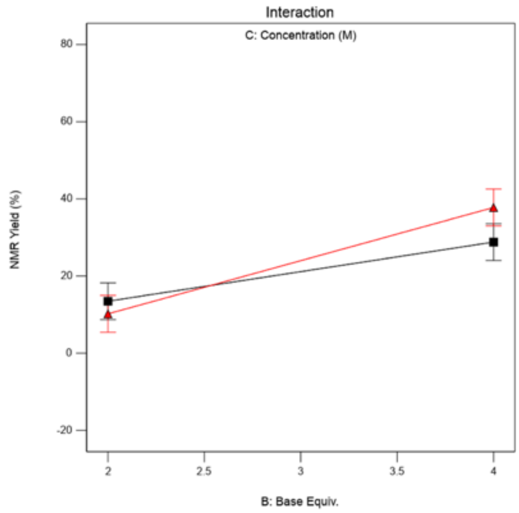

Figure S4: Interaction between base stoichiometry and concentration

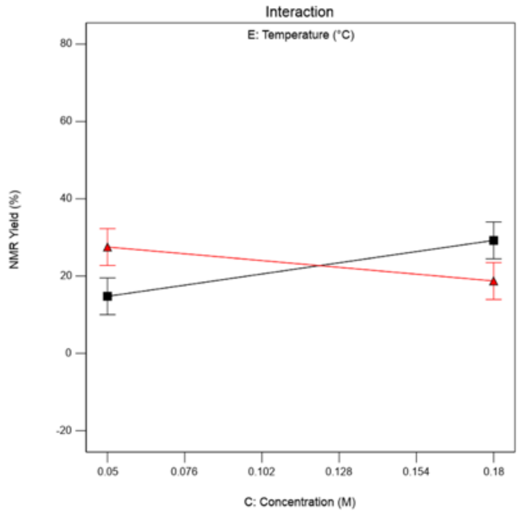

Figure S5: Interaction between concentration and temperature

Figure S6: 3-D Response Curve showing the effects of both KBr and base stoichiometry upon ^{1}H NMR yield (concentration = 0.18 M, time = 3 h, temperature = 30 °C)

Hydrazine Auxiliary Screen

General Procedure A: Synthesis of Hydrazone Substrates

To a stirred solution of 4-methylbenzaldehyde (1.0 equiv.) in ethanol (10 mL) was added the appropriate hydrazine (1.0–1.1 equiv.) followed by a few drops of concentrated sulfuric acid. The resulting suspension was stirred at rt for 16 h. The reaction mixture was filtered, and the resulting precipitate was washed with 2 M aqueous hydrochloric acid, water and ethanol and then dried under high-vacuum to afford the desired product.

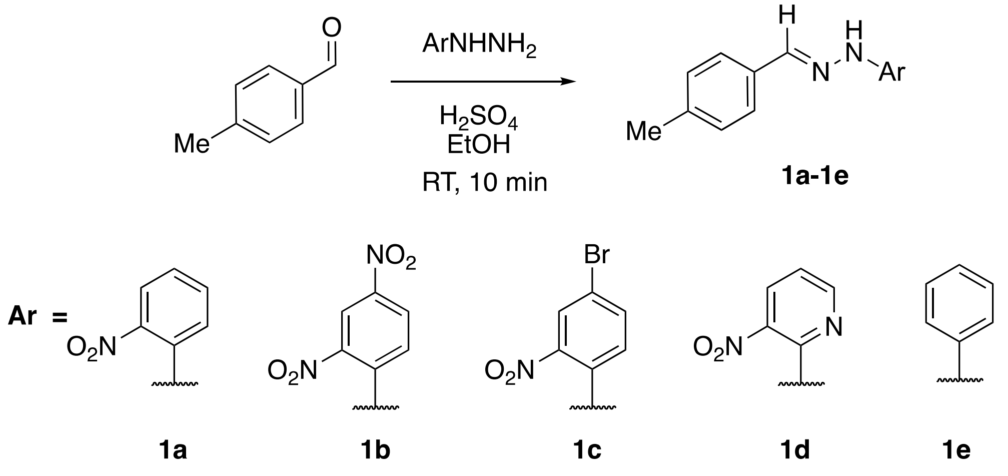

(*E*)-1-(4-Methylbenzylidene)-2-(2-nitrophenyl)hydrazine (1a)

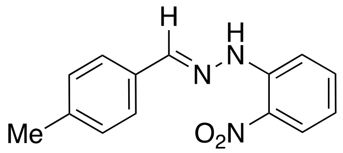

The reaction was performed according to general procedure A using 4-methylbenzaldehyde (491 µL, 4.16 mmol) and 2-nitrophenylhydrazine hydrochloride (868 mg, 4.58 mmol) in ethanol (10 mL). This afforded (*E*)-1-(4-methylbenzylidene)-2-

(2-nitrophenyl)hydrazine (**1a**) (783 mg, 74%) as a red solid. FT-IR ν_{max} (neat) 3292, 1616, 1574, 1497, 1418, 1337, 1327, 1263, 1215, 1175, 1119, 1092, 993, 810, 737 cm^{-1}; ^1H NMR (500 MHz, $CDCl_3$) δ 11.02 (s, 1H), 8.17 (dd, *J* = 8.6, 1.6 Hz, 1H), 8.00 (d, *J* = 10.2 Hz, 1H)), 7.63 (d, *J* = 7.8 Hz, 2H), 7.55 (ddd, *J* = 8.6, 6.9, 1.6 Hz, 1H), 7.23 (d, *J* = 7.8 Hz, 2H), 6.83 (ddd, *J* = 8.6, 6.9, 1.6 Hz, 1H), 2.40 (s, 3H); $^{13}C\{^1H\}$ NMR (126 MHz, $CDCl_3$) δ 144.1, 142.2, 140.3, 136.3, 131.7, 131.1, 129.7 (2 × CH), 126.1, 121.2 (2 × CH), 118.2, 116.4, 21.7; HRMS (ESI) m/z: $[M + H]^+$ Calcd. for $C_{14}H_{14}N_3O_2$ 256.1081; Found 256.1077.

(*E*)-1-(2,4-Dinitrophenyl)-2-(4-methylbenzylidene)hydrazine (1b)

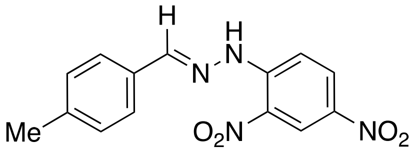

The reaction was performed according to general procedure A using 4-methylbenzaldehyde (200 µL, 1.70 mmol) and 2,4-dinitrophenylhydrazine (299 mg, 1.51 mmol). The reaction mixture was stirred at room temperature for 16 h. This afforded (*E*)-1-(2,4-dinitrophenyl)-2-(4-methylbenzylidene)hydrazine (**1b**) (329 mg, 73%) as an orange solid. FT-IR ν_{max} (neat) 3285, 1611, 1582, 1501, 1420, 1321, 1312, 1269, 1221, 1132, 1078, 816, 743, 716 cm^{-1}; ^1H NMR (500 MHz, DMSO-d_6) δ 11.58 (s, 1H), 8.81 (s, 1H), 8.62 (s, 1H), 8.33 (d, *J* = 9.6 Hz, 1H), 8.04 (d, *J* = 9.6 Hz, 1H), 7.65 (d, *J* = 7.7 Hz, 2H), 7.27 (d, *J* = 7.7 Hz, 2H), 2.35 (s, 3H); $^{13}C\{^1H\}$ NMR (126 MHz, DMSO-d_6) δ 149.5, 144.5, 140.5, 136.9, 131.1, 129.7, 129.5 (2 × CH), 129.3, 127.3 (2 × CH), 123.0, 116.7, 21.1; HRMS (ESI) m/z: $[M - H]^+$ Calcd. for $C_{14}H_{11}N_4O_4$ 299.0786; Found 299.0788.

(*E*)-1-(4-Bromo-2-nitrophenyl)-2-(4-methylbenzylidene)hydrazine (1c)

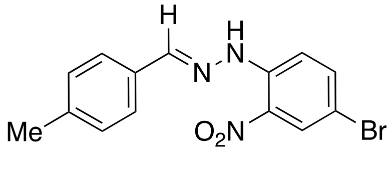

The reaction was performed according to general procedure A using 4-methylbenzaldehyde (196 µL, 1.66 mmol) and 2-nitro-4-bromohydrazine (425 mg, 1.83 mmol). This afforded (*E*)-1-(4-bromo-2-nitrophenyl)-2-(4-methylbenzylidene)hydrazine (**1c**) (550 mg, quant.) as a red solid. FT-IR ν_{max} (neat) 3283, 1614, 1560, 1493, 1425, 1335, 1308, 1260, 1217, 1138, 1096, 1063, 810 cm^{-1}; ^{1}H NMR (400 MHz, $CDCl_3$) δ 10.95 (s, 1H), 8.31 (d, J = 2.3 Hz, 1H), 7.98 (s, 1H), 7.90 (d, J = 9.2 Hz, 1H), 7.72–7.52 (m, 3H), 7.23 (d, J = 7.8 Hz, 2H), 2.40 (s, 3H); ^{13}C{^{1}H} NMR (101 MHz, $CDCl_3$) δ 144.9, 141.2, 140.7, 139.0, 131.4, 131.2, 129.8 (2 × CH), 128.2, 127.3 (2 × CH), 118.2, 109.7, 21.7; HRMS (ESI) m/z: [M + H]$^+$ Calcd. for $C_{14}H_{13}{}^{79}BrN_3O_2$ 334.0186; Found 334.0183.

(*E*)-2-(2-(4-Methylbenzylidene)hydrazineyl)-3-nitropyridine (1d)

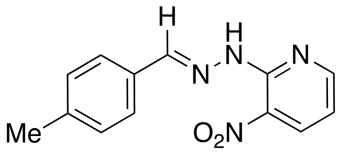

The reaction was performed according to general procedure A using 4-methylbenzaldehyde (196 µL, 1.66 mmol) and 2-hydrazino-3-nitropyridine (426 mg, 1.83 mmol). This afforded (*E*)-2-(2-(4-Methylbenzylidene)hydrazineyl)-3-nitropyridine (**1d**) (424 mg, quant.) as an orange solid. FT-IR ν_{max} (neat) 3296, 1643, 1595, 1572, 1528, 1491, 1443, 1292, 1256, 1229, 1198, 1142, 1074, 1036, 816, 746 cm^{-1}; ^{1}H NMR (400 MHz, DMSO-d_6) δ 11.52 (s, 1H), 8.54 (d, J = 4.7 Hz, 1H), 8.50–8.33 (m, 3H), 7.60 (d, J = 7.7 Hz, 2H), 7.26 (d, J = 7.7 Hz, 2H), 7.02 (dd, J = 8.2, 4.7 Hz, 1H), 6.08 (s, 1H), 2.34 (s, 3H); ^{13}C{^{1}H} NMR (101 MHz, DMSO-d_6) δ 152.9, 147.7, 146.8, 139.7, 135.8, 131.6, 129.9, 129.4 (2 × CH), 127.0 (2 × CH), 114.5, 21.0; HRMS (ESI) m/z: [M + H]$^+$ Calcd. for $C_{13}H_{13}N_4O_2$ 257.1033; Found 257.1027.

(*E*)-1-(4-methylbenzylidene)-2-phenylhydrazine (1e)

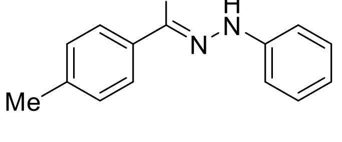

The reaction was performed according to general procedure A using 4-methylbenzaldehyde (4.24 mL, 36.0 mmol) and phenylhydrazine (3.55 mL, 36.0 mmol). The reaction mixture was stirred at room temperature for 16 h. This afforded (*E*)-1-(4-methylbenzylidene)-2-phenylhydrazine (**1e**) (2.25 g, 30%) as a red solid. ^{1}H NMR (500 MHz, DMSO-d_6) δ 10.24 (s, 1H), 7.84 (s, 1H), 7.53 (d, *J* = 7.9 Hz, 2H), 7.24 – 7.16 (m, 4H), 7.06 (d, *J* = 7.9 Hz, 2H), 6.73 (t, *J* = 7.2 Hz, 1H), 2.31 (s, 3H); $^{13}C\{^{1}H\}$ NMR (126 MHz, DMSO-d_6) δ 145.40, 137.35, 136.60, 133.10, 129.22 (2 × CH), 129.05 (2 × CH), 125.58 (2 × CH), 118.51, 111.88 (2 × CH), 20.89; LCMS (ESI) m/z: $[M + H]^+$ Calcd. for $C_{14}H_{15}N_2$ 211.1230; Found 211.200.

General Procedure for Hydrazone Amidation

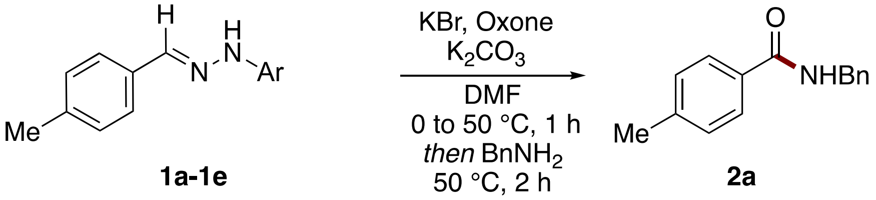

The requisite hydrazone **1a-1e** was dissolved in *N*,*N*-dimethylformamide (3 mL) and cooled to 0°C and then potassium bromide (2 equiv.), Oxone® (3 equiv.) and potassium carbonate (5 equiv.) were added. The resulting suspension was stirred at 0 °C for 10 minutes, warmed to 50 °C and stirred for 2 h. Benzylamine (5 equiv.) was added and the mixture was stirred at 50 °C for 2 h. The reaction mixture was diluted with ethyl acetate (20 mL) and washed with 1 M aqueous hydrochloric acid (2 × 20 mL), 1 M aqueous sodium hydroxide (2 × 20 mL) and then brine (20 mL). The organic phse was dried ($MgSO_4$), filtered, spiked with 1,3,5-trimethoxybenzene (1 equiv.) and concentrated *in vacuo*. The residue was dissolved in $CDCl_3$ (0.7 mL) and analysed *via* ^1H NMR spectroscopy.

Entry	Hydrazone Substitution (X)	Yield (%)[a]
1	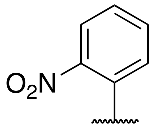	80 (61)[b]
2	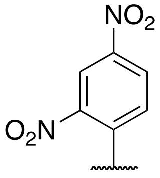	8%
3	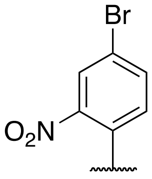	39%
4	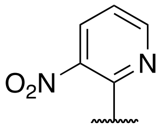	traces
5	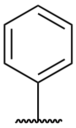	33%[b]

[a]Yield determined by ^{1}H NMR spectroscopy with reference to 1,3,5-trimethoxybenzene. [b]Isolated yield

Isolation of (*E*)-((2-Nitrophenyl)diazenyl)(*p*-tolyl)methanone (3)

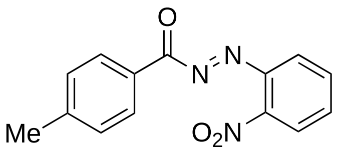

To a stirred solution of 2-nitrophenylhydrazine hydrochloride (94.5 mg, 0.500 mmol) in *N*,*N*-dimethylformamide (3 mL) was added 4-methylbenzaldehyde (65.0 µL, 0.550 mmol). The resulting suspension was stirred at room temperature for 0.5 h, cooled to 0 °C and potassium bromide (119 mg, 1.00 mmol, 2 equiv.), Oxone® (922 mg, 1.5 mmol, 3 equiv.) and potassium carbonate (346 mg, 2.5 mmol, 5 equiv.) were added. The reaction mixture was stirred at 0 °C for 10 minutes then warmed to 50 °C and stirred for 2 h. After cooling to room temperature, the reaction mixture was diluted with ethyl acetate (30 mL) and washed with 1 M aqueous hydrochloric acid (2 × 30 mL), water (2 × 30 mL) and brine (30 mL). The organic phase was dried ($MgSO_4$), filtered and concentrated *in vacuo*. Purification by flash column chromatography using a gradient system (10–20% ethyl acetate in petroleum ether) afforded (*E*)-((2-nitrophenyl)diazenyl)(*p*-tolyl)methanone (**3**) (35.3 mg, 28%) as an orange solid. FT-IR (neat) ν_{max} 2943, 1719, 1619, 1531, 1352, 1264, 1020 cm^{-1}; ^{1}H NMR (400 MHz, $CDCl_3$) δ 8.05–7.99 (m, 1H), 7.98–7.89 (m, 2H), 7.75 (td, *J* = 7.7, 1.5 Hz, 1H), 7.69 (td, *J* = 7.7, 1.6 Hz, 1H), 7.57–7.52 (m, 1H), 7.39–7.32 (m, 2H), 2.46 (s, 3H); $^{13}C\{^{1}H\}$ NMR (101 MHz, $CDCl_3$) δ 181.5, 147.2, 146.5, 145.7, 133.8, 132.3, 131.0 (2 × CH), 129.9 (2 × CH), 126.8, 124.6, 119.0, 22.1; HRMS (ESI) m/z: $[M + H]^+$ Calcd. for $C_{14}H_{12}N_3O_3$ 270.0873; Found 270.0867.

LCMS of crude reaction mixture:

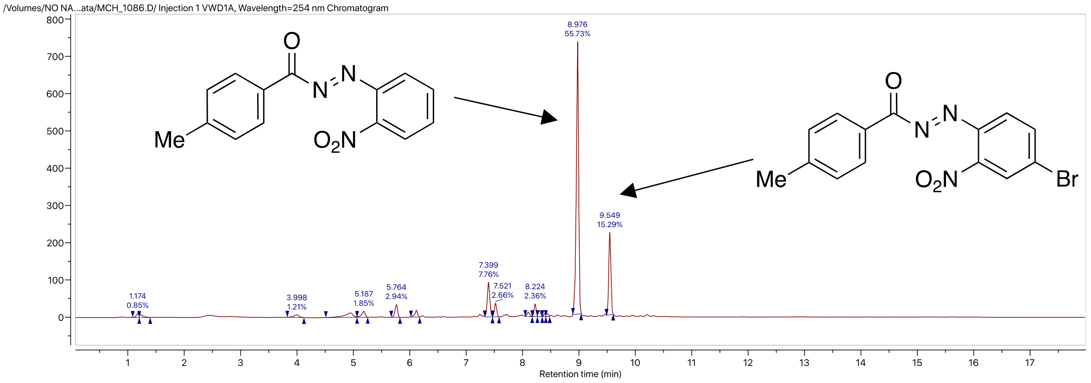

TLC of crude material after work-up (as described above):

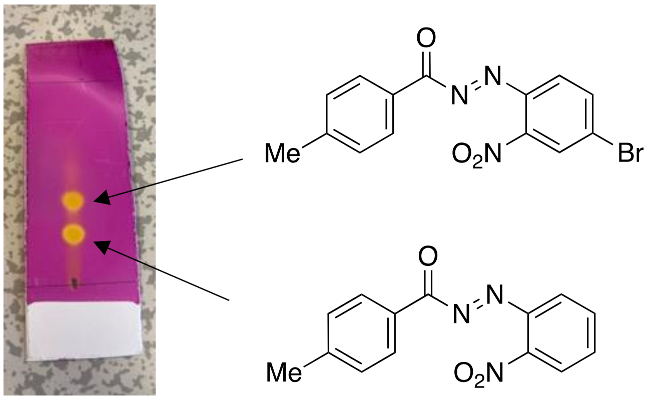

From this reaction, (*E*)-((4-bromo-2-nitrophenyl)diazenyl)(*p*-tolyl)methanone was also isolated (16.4 mg, 9.4%) as an orange solid. R*f* = 0.17 (petroleum ether/diethyl ether, 19:1); FT-IR (neat) ν_{max} 2941, 2874, 1691, 1633, 1544, 1358, 1296, 1274, 1181, 765, 738 cm^{-1}; ^{1}H NMR (400 MHz, $CDCl_3$) δ 8.14 (d, *J* = 2.0 Hz, 1H), 7.94–7.84 (m, 3H), 7.48 (d, *J* = 8.5 Hz, 1H), 7.39–7.32 (m, 2H), 2.46 (s, 3H); $^{13}C\{^1H\}$ NMR (101 MHz, $CDCl_3$) δ 181.2, 147.8, 146.6, 144.1, 136.8, 130.9 (2 × CH), 130.0 (2 × CH), 127.6, 126.7, 126.3, 120.3, 22.1; HRMS (ESI) m/z: $[M + H]^+$ Calcd. for $C_{14}H_{11}{}^{79}BrN_3O_3$ 347.9978; Found 347.9984.

Amidation of (*E*)-((2-Nitrophenyl)diazenyl)(*p*-tolyl)methanone (3)

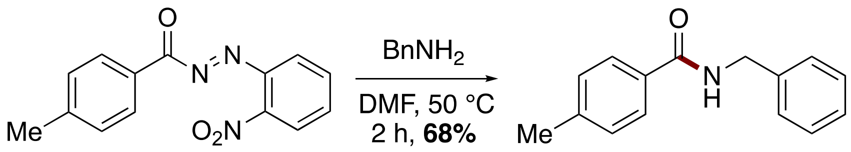

(*E*)-((2-Nitrophenyl)diazenyl)(*p*-tolyl)methanone (50.0 mg, 0.186 mmol) was dissolved in *N*,*N*-dimethylformamide (2 mL) and benzyl amine (101 µL, 0.928 mmol) was added and the mixture was stirred at 50 °C for 2 h. After cooling to room temperature, the reaction mixture was diluted with ethyl acetate (20 mL) and washed with 1 M aqueous hydrochloric acid (2 × 20 mL) then brine (20 mL). The organic phase was dried ($MgSO_4$), filtered and concentrated *in vacuo*. Purification by flash column chromatography (dichloromethane) afforded *N*-benzyl-4-methylbenzamide (**2a**) (28.5 mg, 68%) as an orange solid. Characterisation data as previously reported for *N*-benzyl-4-methylbenzamide (**2a**).

LCMS UV trace of crude reaction mixture (2 h after amine addition):

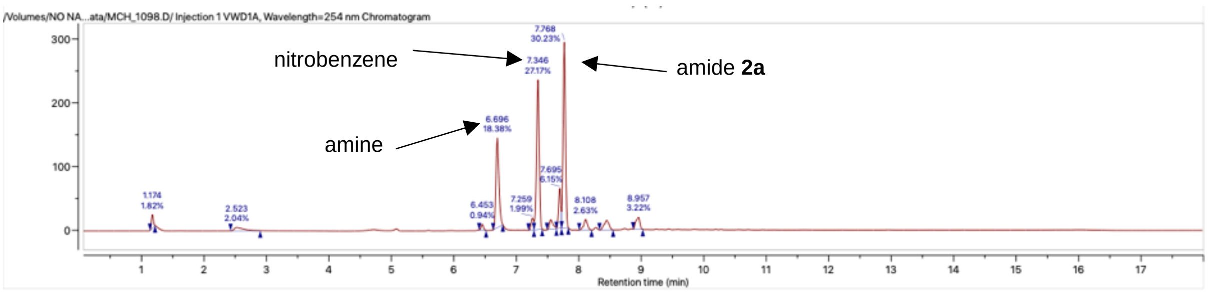

4-Methyl-*N*'-(2-nitrophenyl)benzohydrazide (4)

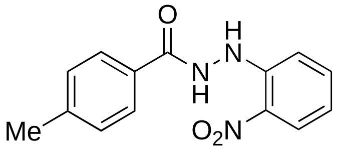

4-Methylbenzoic acid (200 mg, 1.47 mmol) and 2-nitrophenylhydrazine hydrochloride (334 mg, 1.76 mmol) were suspended in *N*,*N*-dimethylformamide (10 mL) and triethylamine (615 µL, 4.41 mmol) was added. The mixture was cooled to 0 °C and propanephosphonic acid anhydride, T3P (1.40 mL, 2.21 mmol; 50% in ethyl acetate) was added dropwise. The reaction mixture was warmed to room temperature and stirred for 20 h. The reaction mixture was diluted with ethyl acetate (30 mL) and washed with 1 M aqueous hydrochloric acid (2 × 30 mL), water (3 × 30 mL) then brine (30 mL). The organic phase was dried ($MgSO_4$), filtered and concentrated *in vacuo*. Purification by flash column chromatography (petroleum ether/ethyl acetate, 1:1) afforded 4-methyl-*N*'-(2-nitrophenyl)benzohydrazide (**4**) (168 mg, 42%) as a dark orange solid. FT-IR (neat) ν_{max} 3380, 3180, 2936, 2865, 1651, 1629, 1580, 1522, 1500, 1354, 1332, 1270, 1225, 1146, 853, 742 cm^{-1}; ^1H NMR (500 MHz, $CDCl_3$) δ 9.02 (s, 1H), 8.09 (dd, *J* = 8.5, 1.5 Hz, 1H), 8.04 (s, 1H), 7.68 (d, *J* = 8.0 Hz, 2H), 7.38 (ddd, *J* = 8.6, 7.0, 1.5 Hz, 1H), 7.19 (d, *J* = 7.6 Hz, 2H), 7.08 (dd, *J* = 8.5, 1.2 Hz, 1H), 6.79 (ddd, *J* = 8.5, 7.0, 1.3 Hz, 1H), 2.35 (s, 3H); $^{13}C\{^1H\}$ NMR (126 MHz, $CDCl_3$) δ 145.4, 143.6, 136.2, 133.6, 129.7, 129.0, 127.4, 126.6, 119.1, 114.5, 21.7; HRMS (ESI) m/z: $[M + H]^+$ Calcd. for $C_{14}H_{14}N_3O_3$ 272.1030; Found 272.1028.

Amidation of 4-Methyl-*N*'-(2-nitrophenyl)benzohydrazide (4)

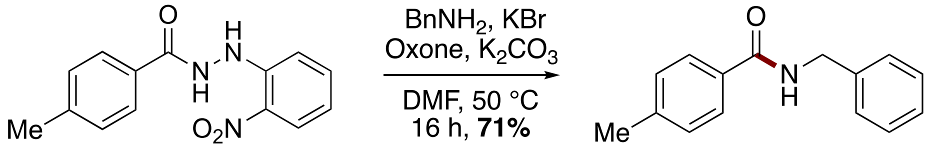

4-Methyl-*N*'-(2-nitrophenyl)benzohydrazide (**4**) (50.0 mg, 0.184 mmol) was dissolved in *N*,*N*-dimethylformamide (2 mL) and cooled to 0 °C. Potassium bromide (44.0 mg, 0.368 mmol), Oxone® (339 mg, 0.552 mmol) and potassium carbonate (127 mg, 0.922 mmol) were added simultaneously. Benzyl amine (101 µL, 0.928 mmol) was added

and the reaction mixture was stirred at 50 °C for 16 h. After cooling to room temperature, the reaction mixture was diluted with ethyl acetate (20 mL) and washed with 1 M aqueous hydrochloric acid (2 × 20 mL) then brine (20 mL). The organic phase was dried ($MgSO_4$), filtered and concentrated *in vacuo*. Purification by flash column chromatography (dichloromethane) afforded *N*-benzyl-4-methylbenzamide (**2a**) (29.3 mg, 68%) as an orange solid. Characterisation data as previously reported for *N*-benzyl-4-methylbenzamide (**2a**).

Isotopic labelling studies with ^{18}O-water

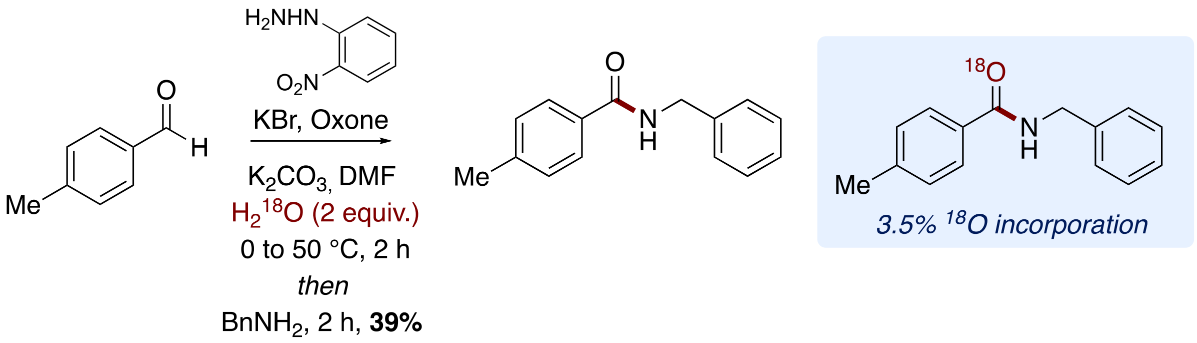

To a stirred solution of 2-nitrophenylhydrazine hydrochloride (94.5 mg, 0.500 mmol) in *N*,*N*-dimethylformamide (2 mL) was added 4-methylbenzaldehyde (65.0 µL, 0.550 mmol). The resulting suspension was stirred at room temperature for 0.5 h, cooled to 0 °C and potassium bromide (119 mg, 1.00 mmol), Oxone® (922 mg, 1.50 mmol) and potassium carbonate (346 mg, 2.50 mmol) were added followed by $H_2{}^{18}O$ (50.0 µL, 2.50 mmol). After stirring at 0 °C for 10 minutes, the reaction mixture was warmed to 50 °C and stirred for 2 h. Benzyl amine (0.273 mL, 2.50 mmol) was added and the mixture was stirred at 50 °C for 2 h. The reaction mixture was diluted with ethyl acetate (20 mL) and washed with 1 M aq. hydrochloric acid (2 × 20 mL) and then brine (20 mL). The organic layer was dried ($MgSO_4$), filtered and concentrated *in vacuo*. The crude mixture was analysed directly *via* HRMS.

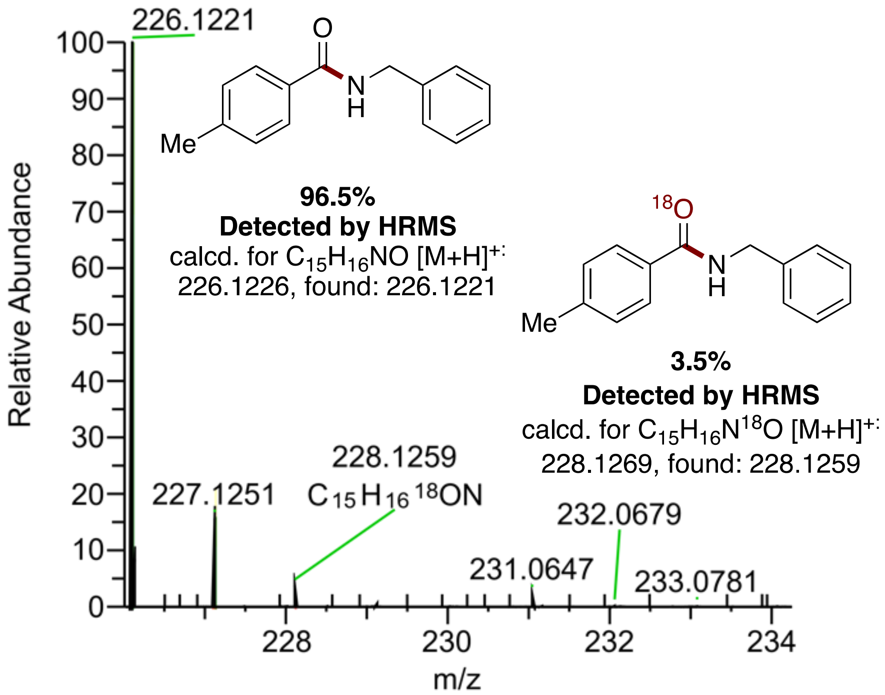

The crude mixture was then spiked with 1,3,5-trimethoxybenzene (84.1 mg, 0.500 mmol) and dissolved in $CDCl_3$ (0.7 mL) and analysed *via* ^{1}H NMR spectroscopy. This showed 39% conversion to amide **2a**.

One-Pot Amidation of 4-Methylbenzaldehyde with Nitrogen Sparging

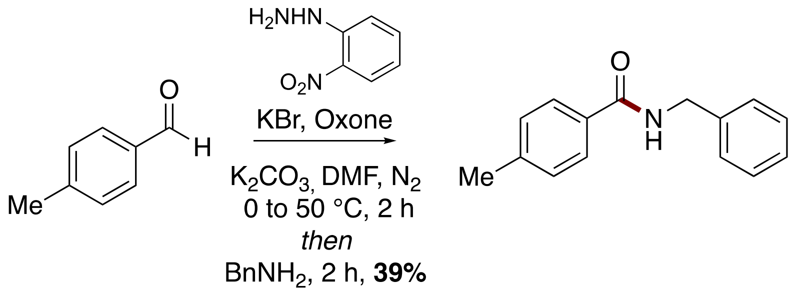

To a stirred solution of 2-nitrophenylhydrazine hydrochloride (94.5 mg, 0.500 mmol) in *N*,*N*-dimethylformamide (3 mL) was added 4-methylbenzaldehyde (65.0 µL, 0.550 mmol). The resulting suspension was sparged with nitrogen gas for 0.5 h with sonication and then stirred at room temperature for 0.5 h. The mixture was then cooled to 0 °C and potassium bromide (119 mg, 1.00 mmol, 2 equiv.), Oxone® (922 mg, 1.5 mmol, 3 equiv.) and potassium carbonate (346 mg, 2.5 mmol, 5 equiv.) were added. The reaction mixture was stirred at 0 °C for 10 minutes then warmed to 50 °C and stirred for 2 h. After cooling to room temperature, the reaction mixture was diluted with ethyl acetate (30 mL) and washed with 1 M aqueous hydrochloric acid (2 × 30 mL),

water (2 × 30 mL) and brine (30 mL). The organic phase was dried ($MgSO_4$), filtered and concentrated *in vacuo*. The crude mixture was then spiked with 1,3,5-trimethoxybenzene (84.1 mg, 0.500 mmol) and dissolved in $CDCl_3$ (0.7 mL) and analysed *via* ^{1}H NMR spectroscopy. This showed 39% conversion to amide **2a**.

Detection of Nitrobenzene

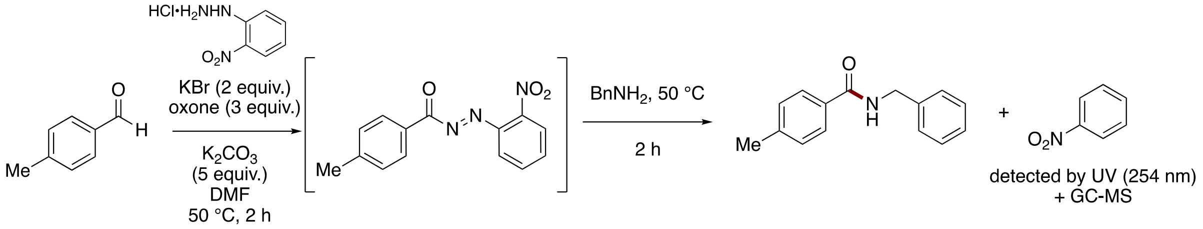

To a stirred solution of 2-nitrophenylhydrazine hydrochloride (94.5 mg, 0.500 mmol) in *N*,*N*-dimethylformamide (3 mL) was added 4-methylbenzaldehyde (65.0 µL, 0.550 mmol). The resulting suspension was stirred at room temperature for 0.5 h, cooled to 0 °C and potassium bromide (119 mg, 1.00 mmol), Oxone® (922 mg, 1.50 mmol) and potassium carbonate (346 mg, 2.50 mmol) were added followed. After stirring at 0 °C for 10 minutes, the reaction mixture was warmed to 50 °C and stirred for 2 h. Benzyl amine (0.273 mL, 2.50 mmol) was added and the mixture was stirred at 50 °C for 2 h. An aliquot of the reaction mixture was removed, dissolved in acetonitrile (1 mL), filtered, and analysed *via* LCMS and GCMS.

TLC of the reaction mixture, 2 h after amine addition:

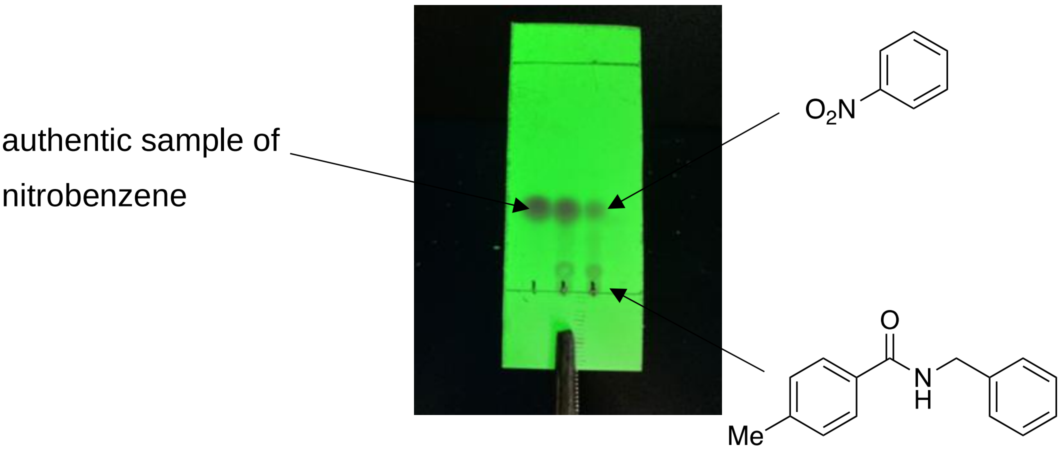

LCMS UV trace of authentic sample of nitrobenzene (254 nm):

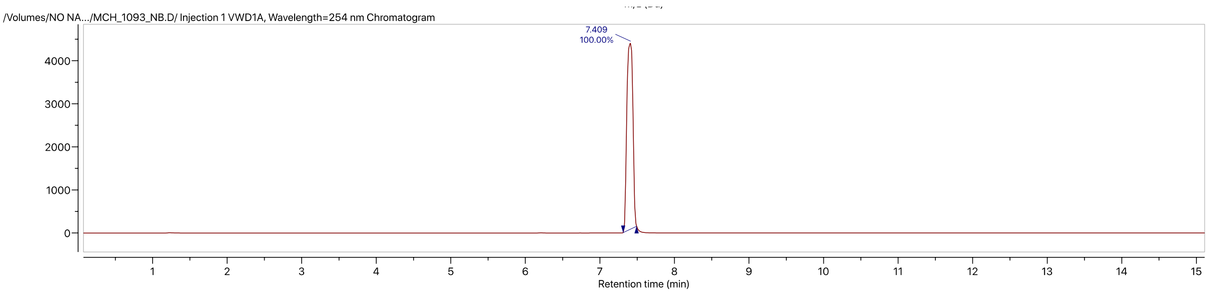

LCMS UV trace of reaction mixture, 2 h after amine addition (254 nm):

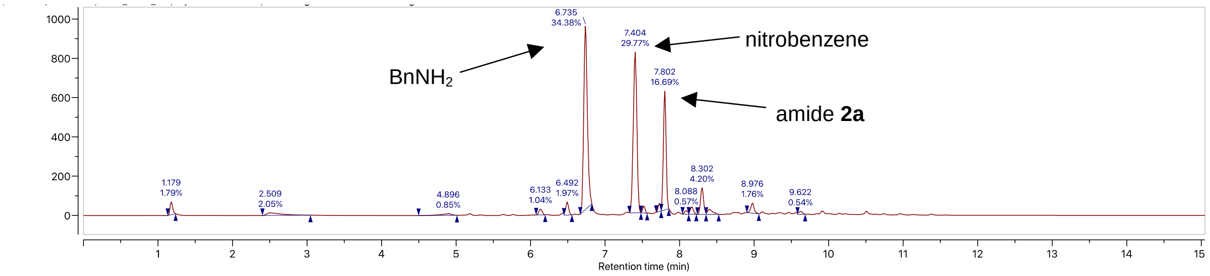

GCMS of reaction mixture: Nitrobenzene detected at 7.70 mins

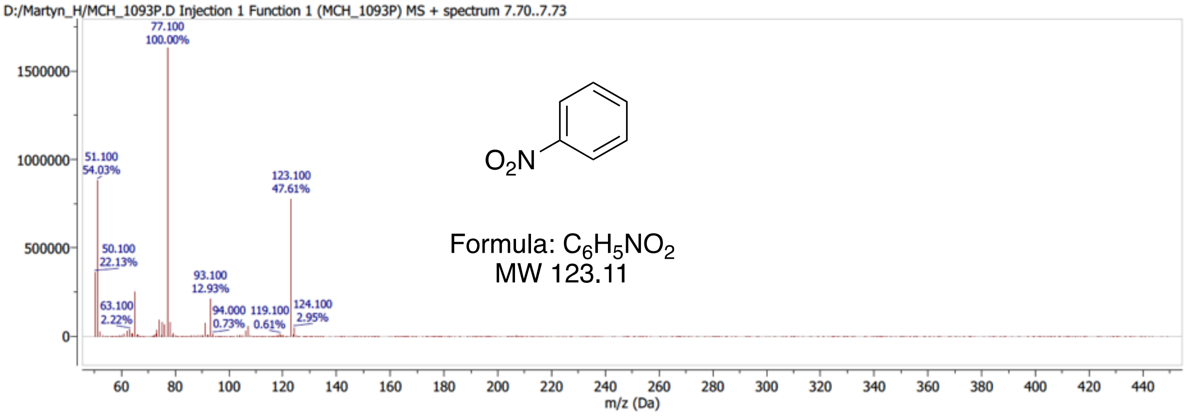

^1H NMR: 500 MHz, $CDCl_3$

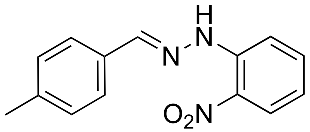

1a

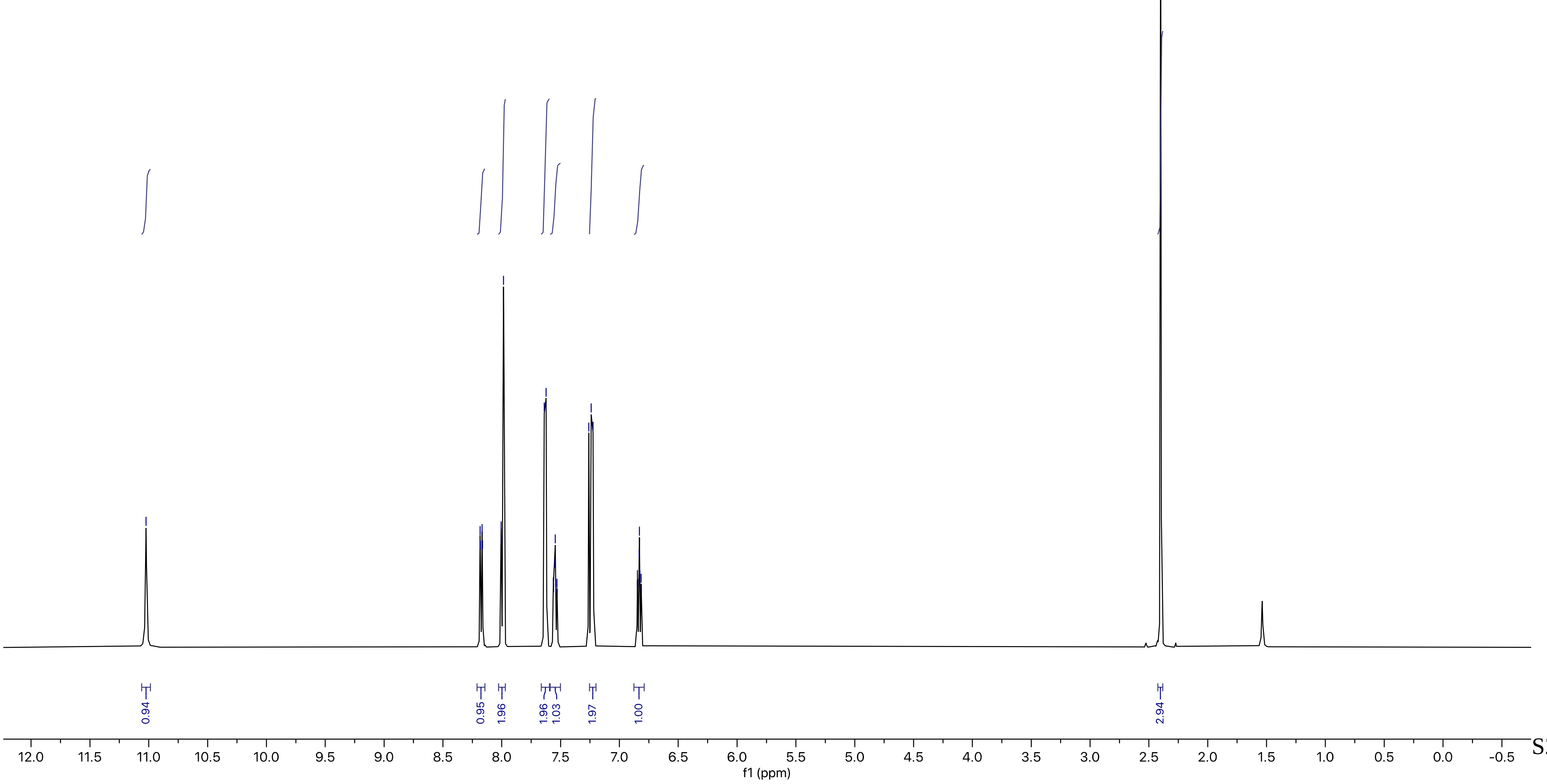

$^{13}C\{^{1}H\}$ NMR: 126 MHz, $CDCl_3$

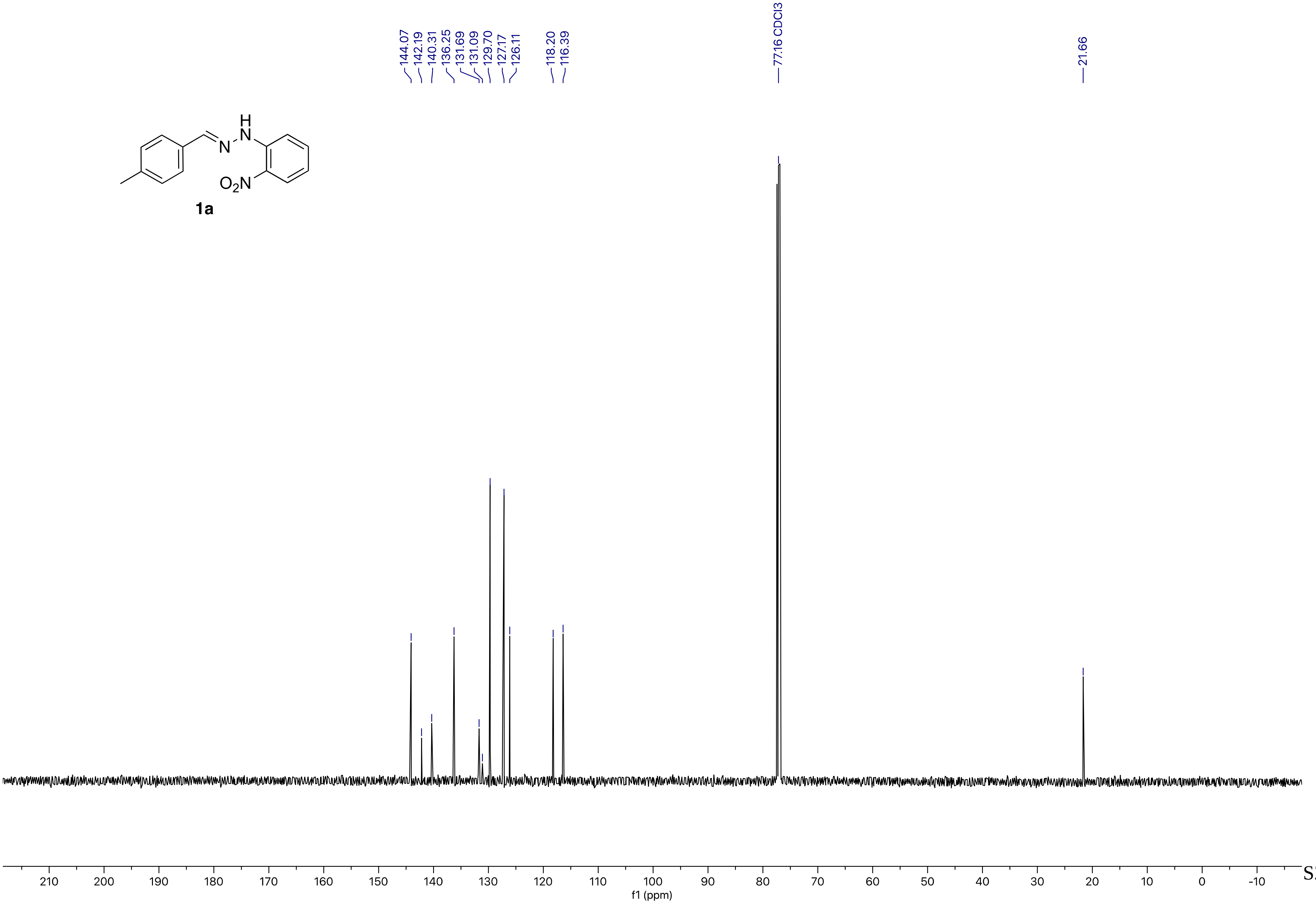

^{1}H NMR: 500 MHz, DMSO-d_6

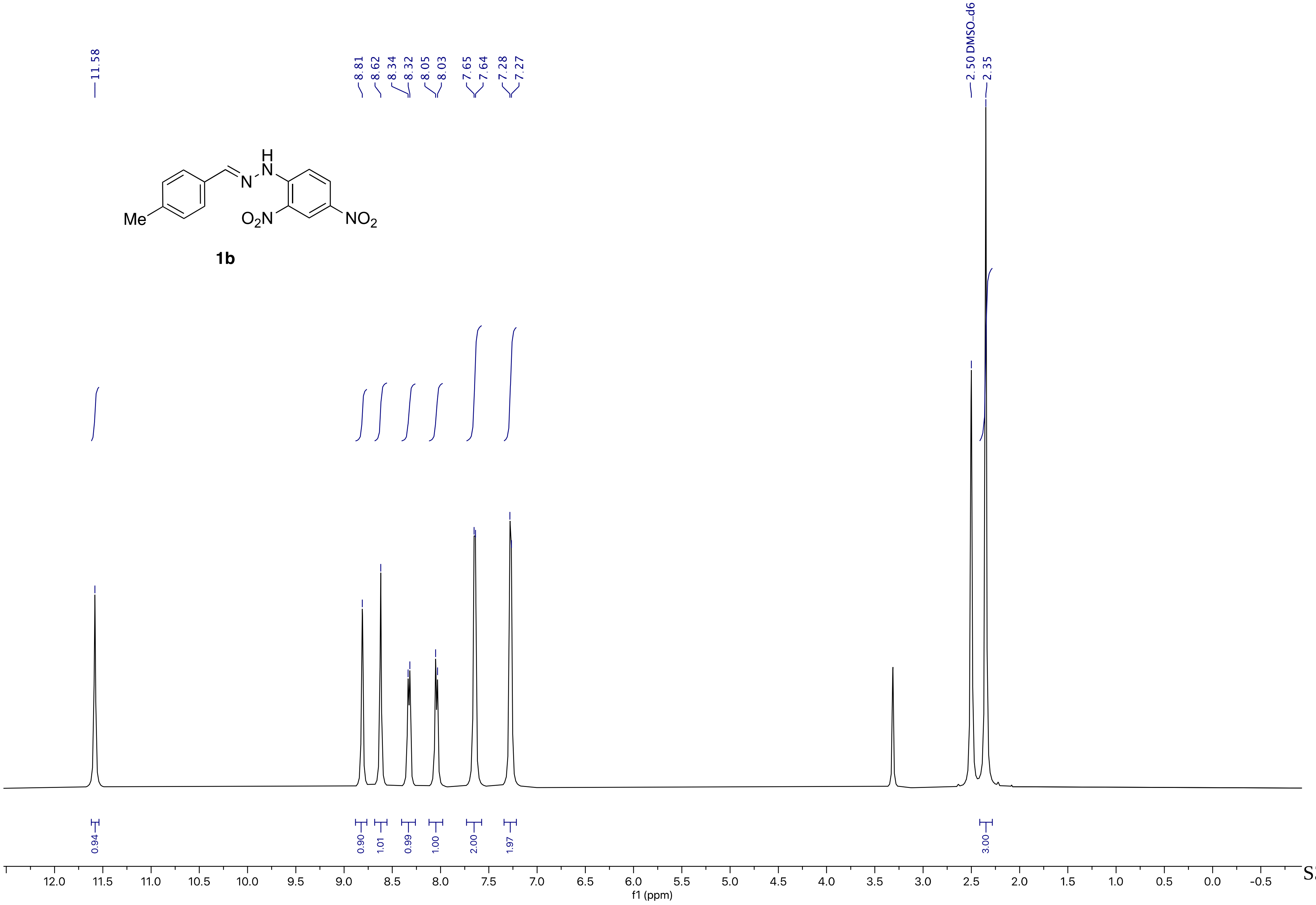

$^{13}C\{^1H\}$ NMR: 126 MHz, DMSO-d_6

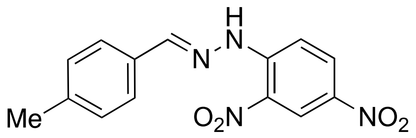

1b

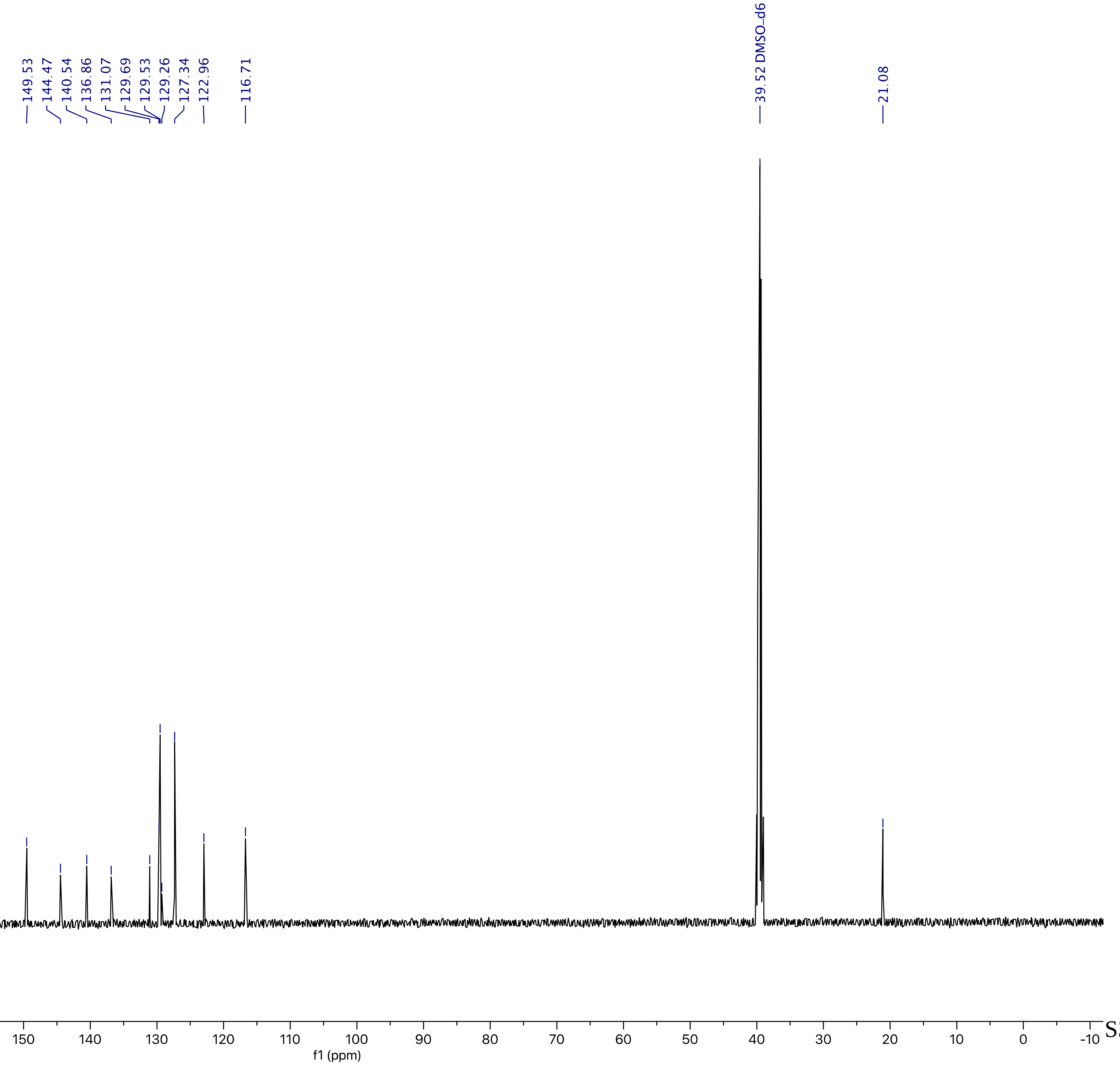

^1H NMR: 400 MHz, $CDCl_3$

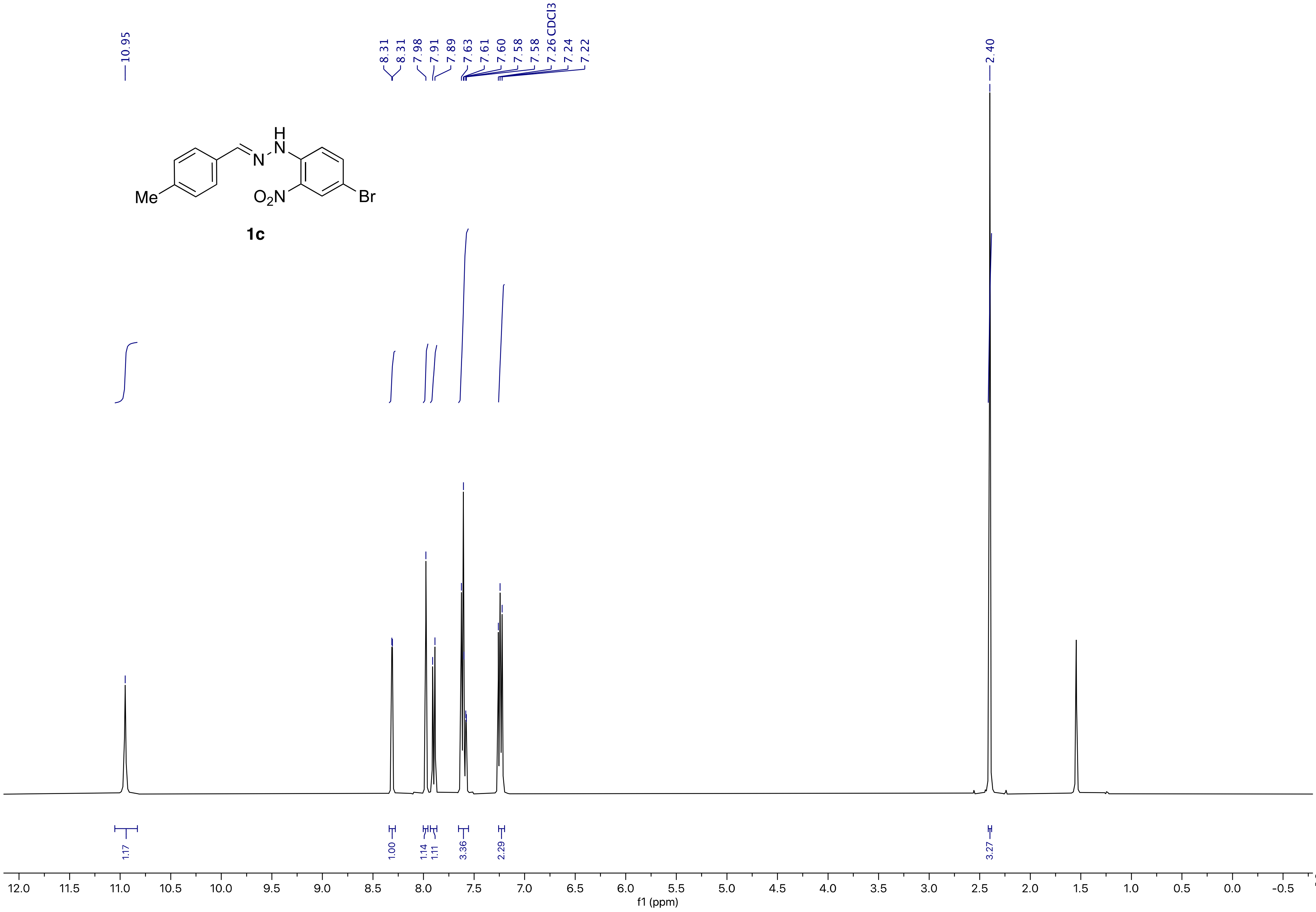

$^{13}C\{^{1}H\}$ NMR: 101 MHz, $CDCl_3$

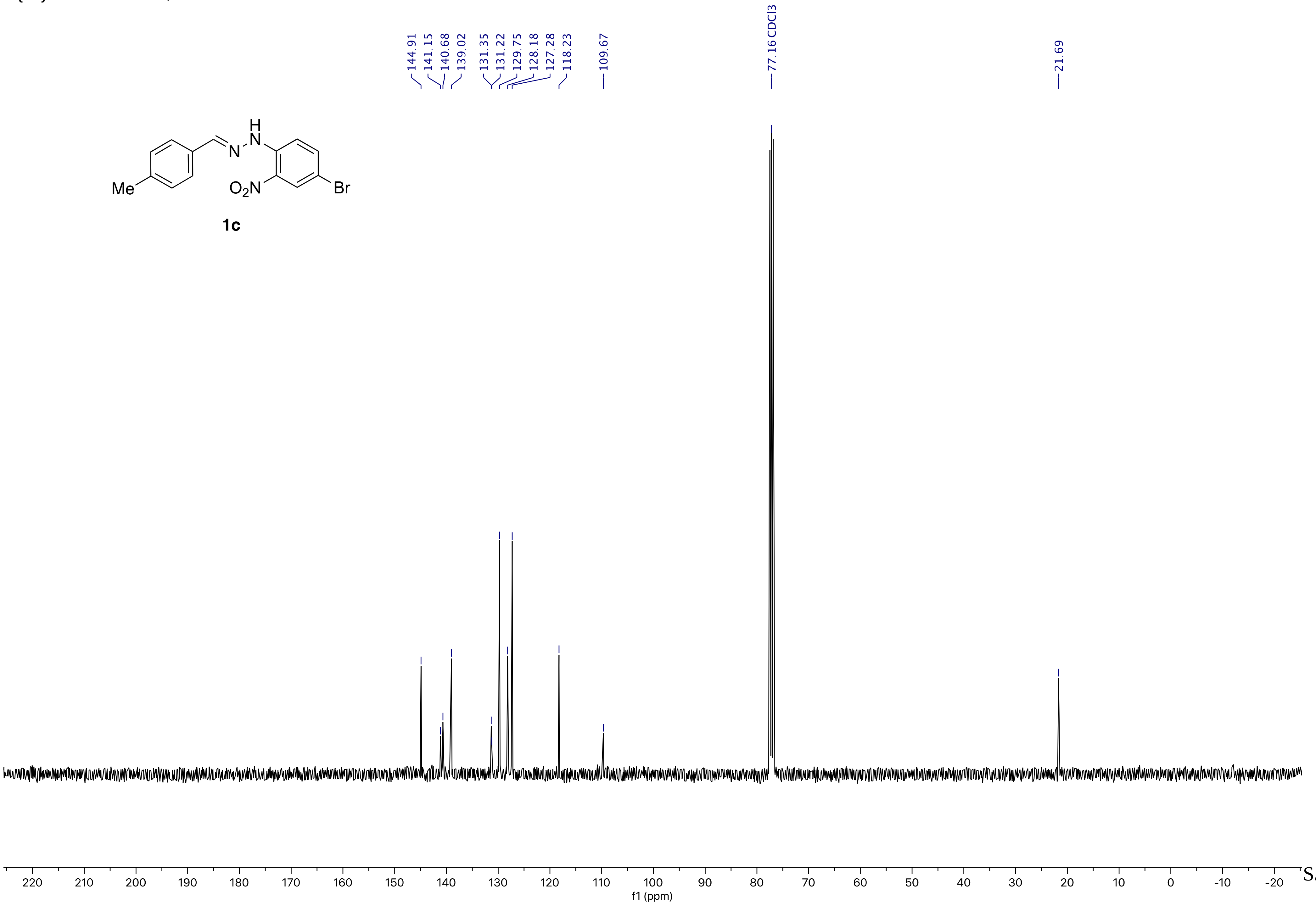

1c

^{1}H NMR: 400 MHz, DMSO-d_6

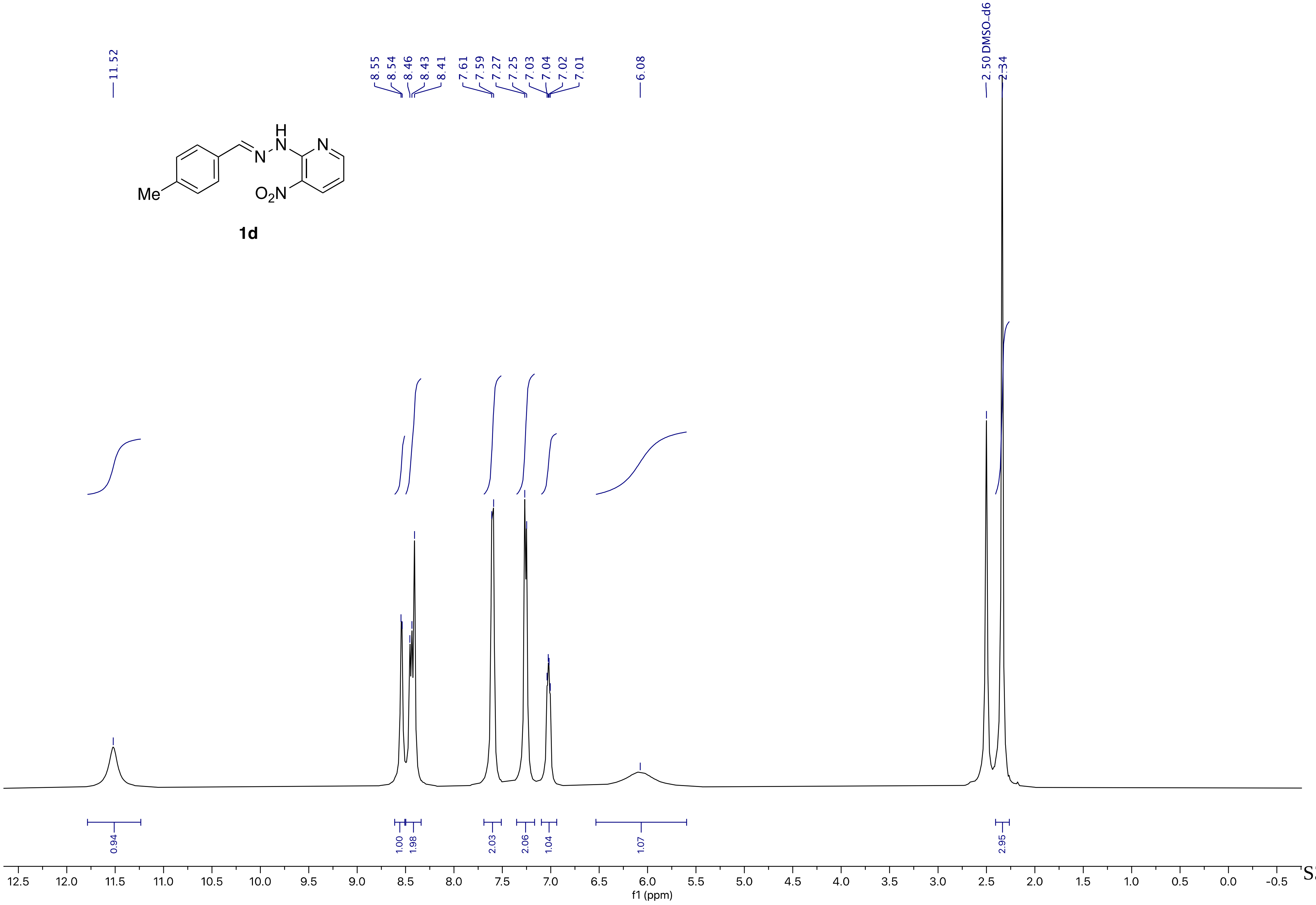

$^{13}C\{^{1}H\}$ NMR: 101 MHz, DMSO-d_6

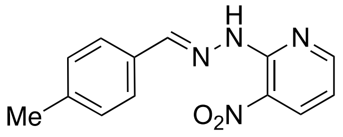

1d

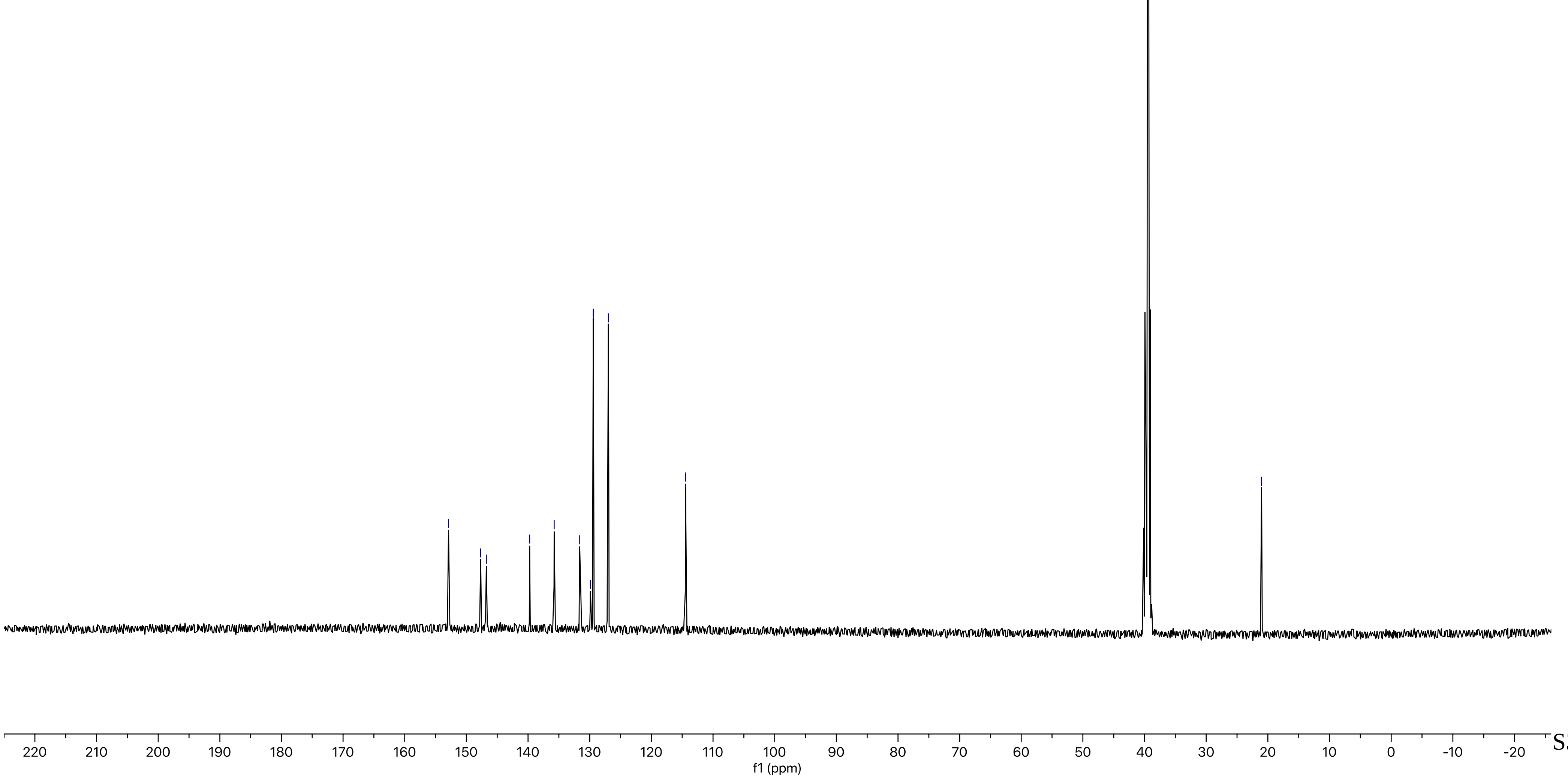

^{1}H NMR: 500 MHz, DMSO-d_6

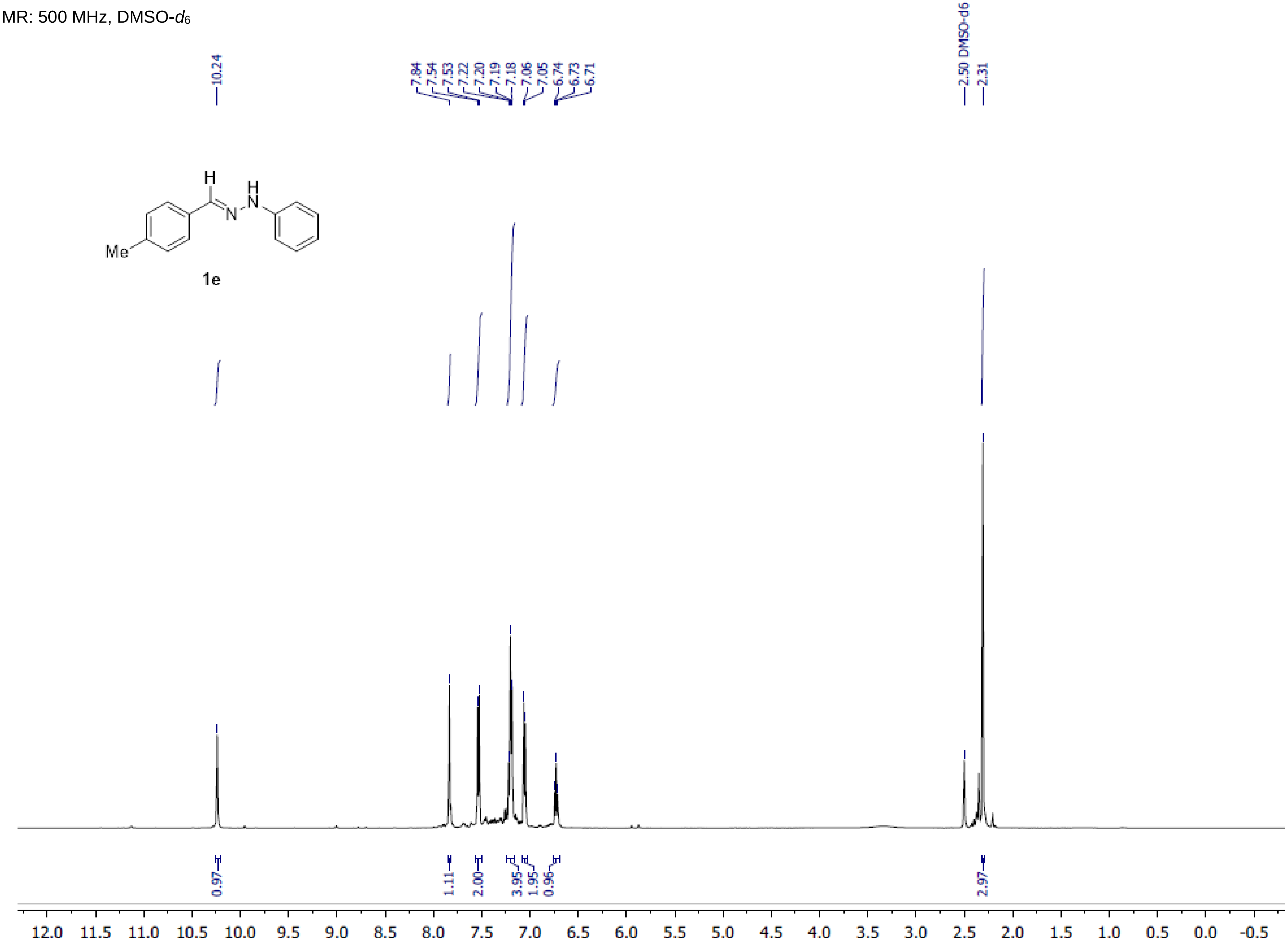

$^{13}C\{^{1}H\}$ NMR: 126 MHz, DMSO-d_6

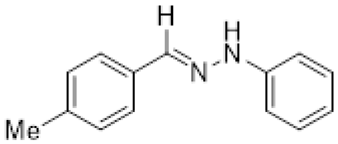

1e

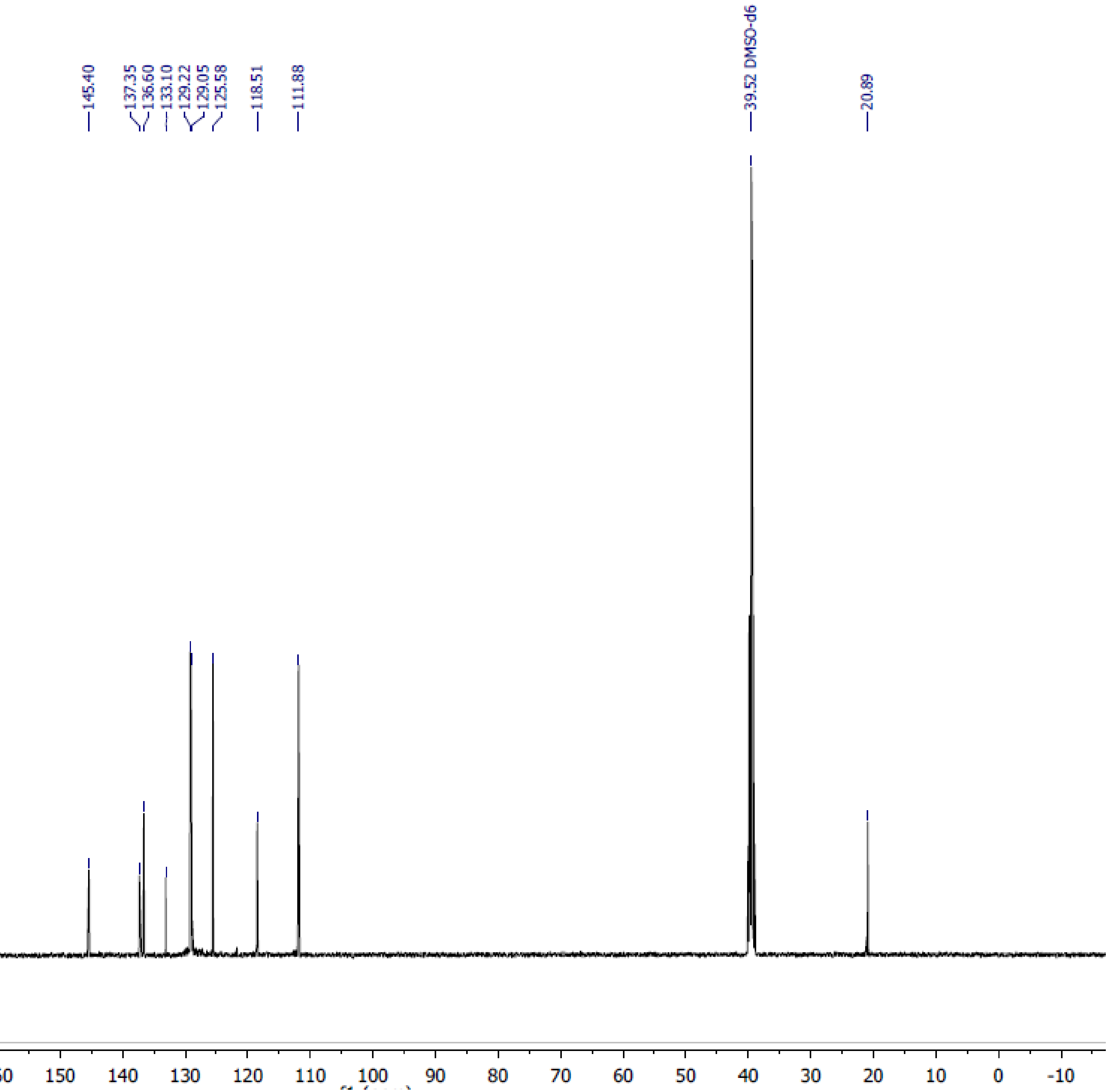

^{1}H NMR: 400 MHz, $CDCl_3$

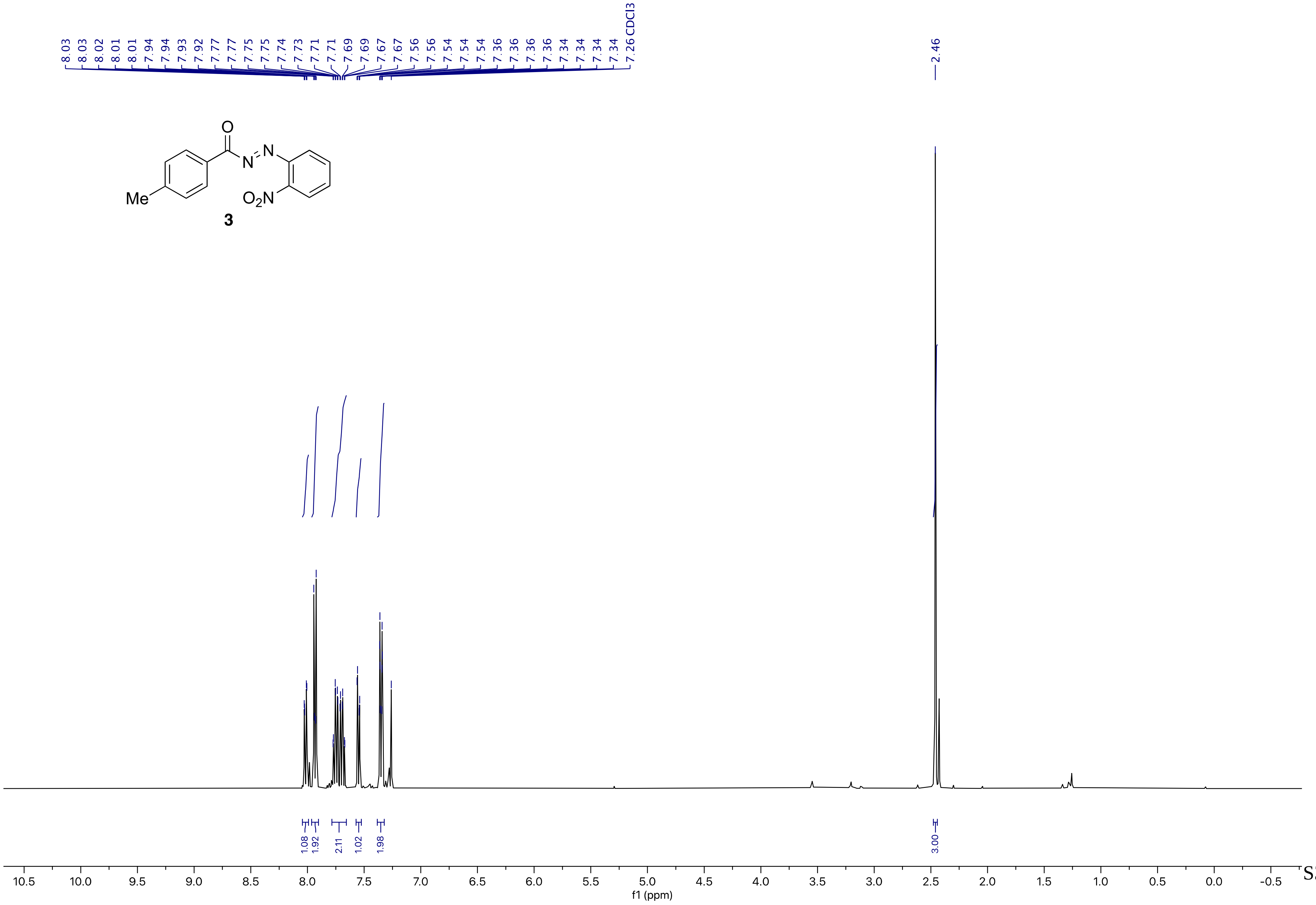

$^{13}C\{^{1}H\}$ NMR: 101 MHz, $CDCl_3$

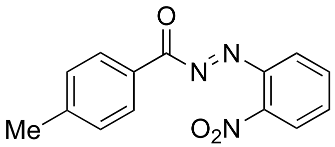

3

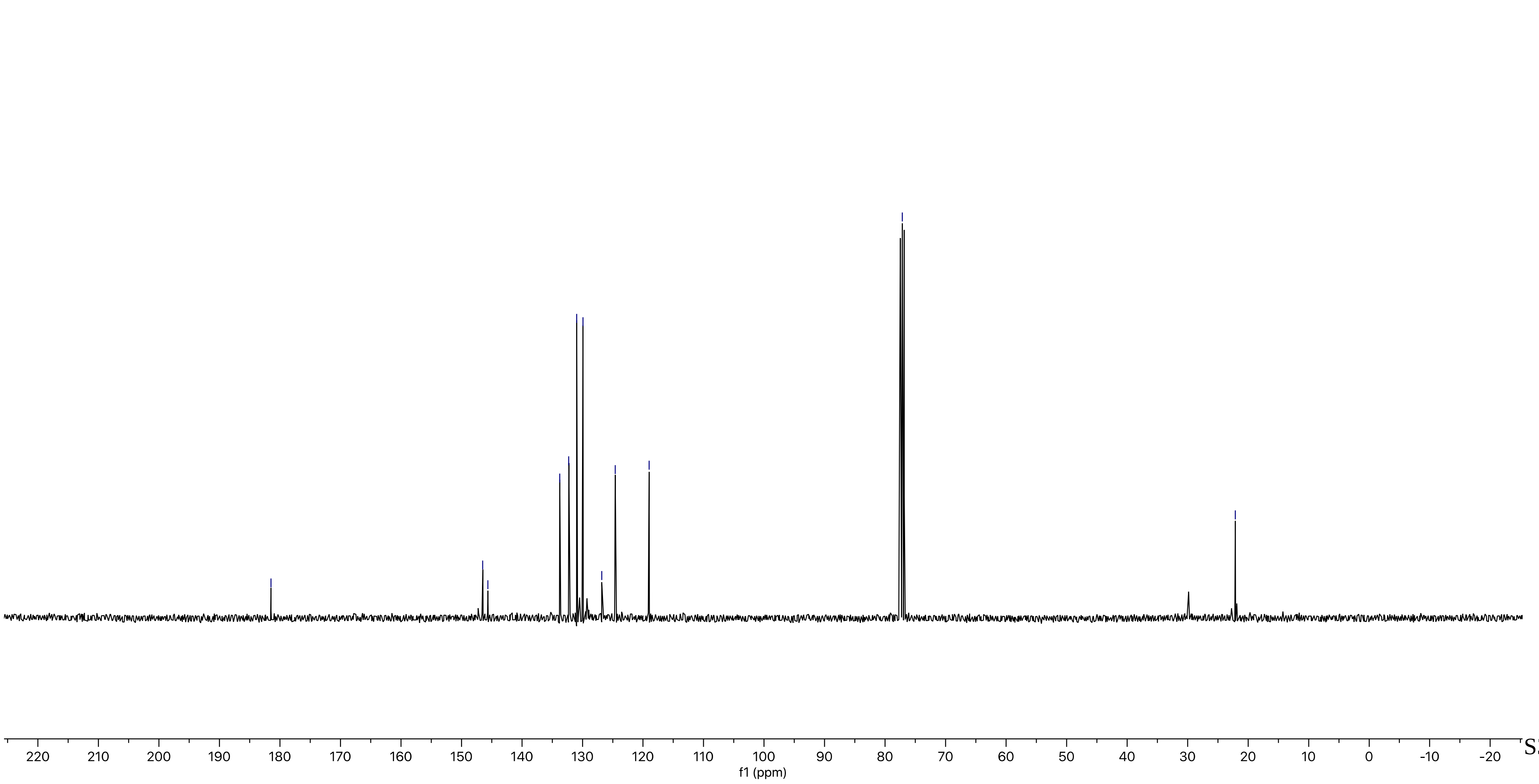

^{1}H NMR: 400 MHz, $CDCl_3$

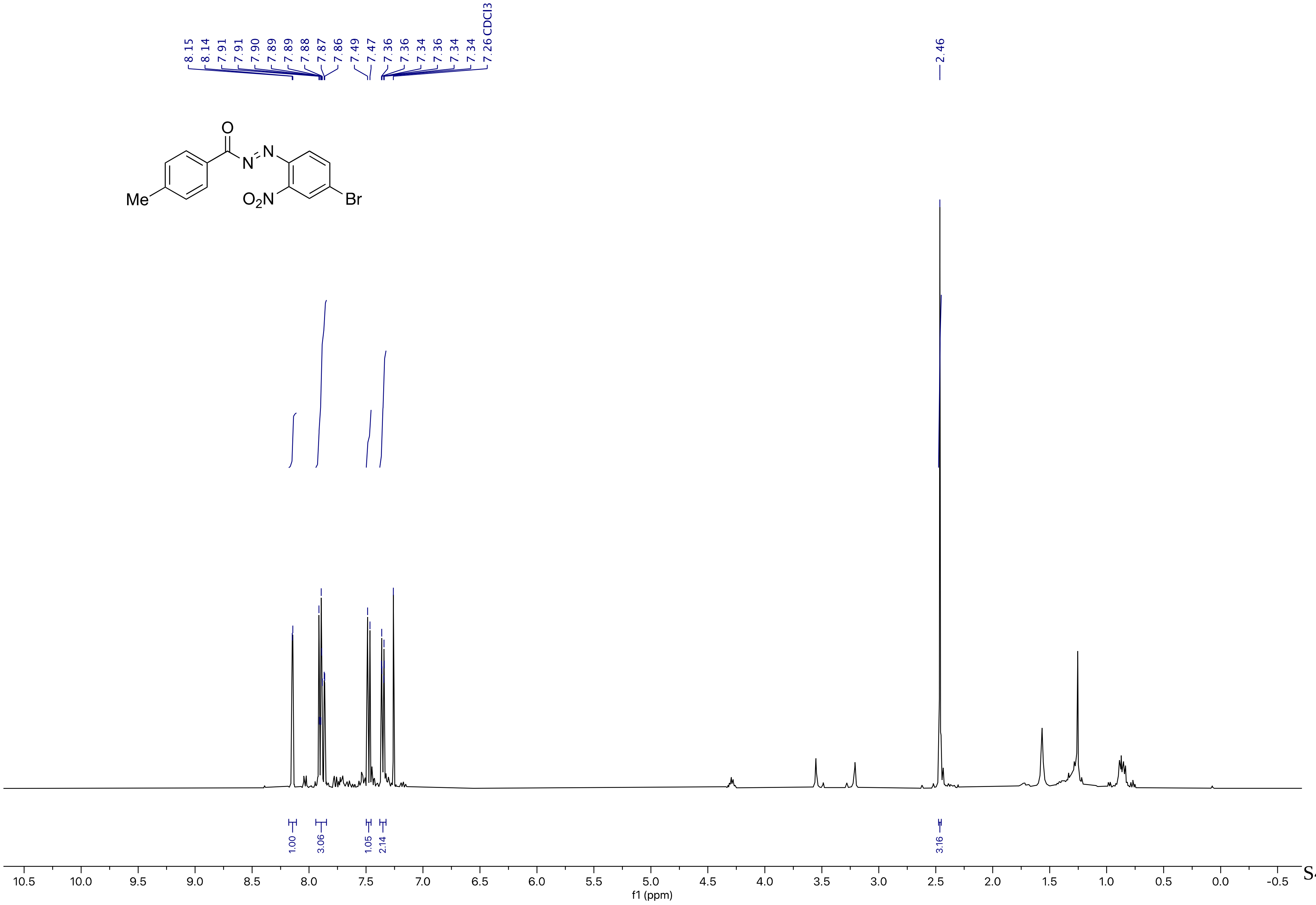

$^{13}C\{^1H\}$ NMR: 101 MHz, $CDCl_3$

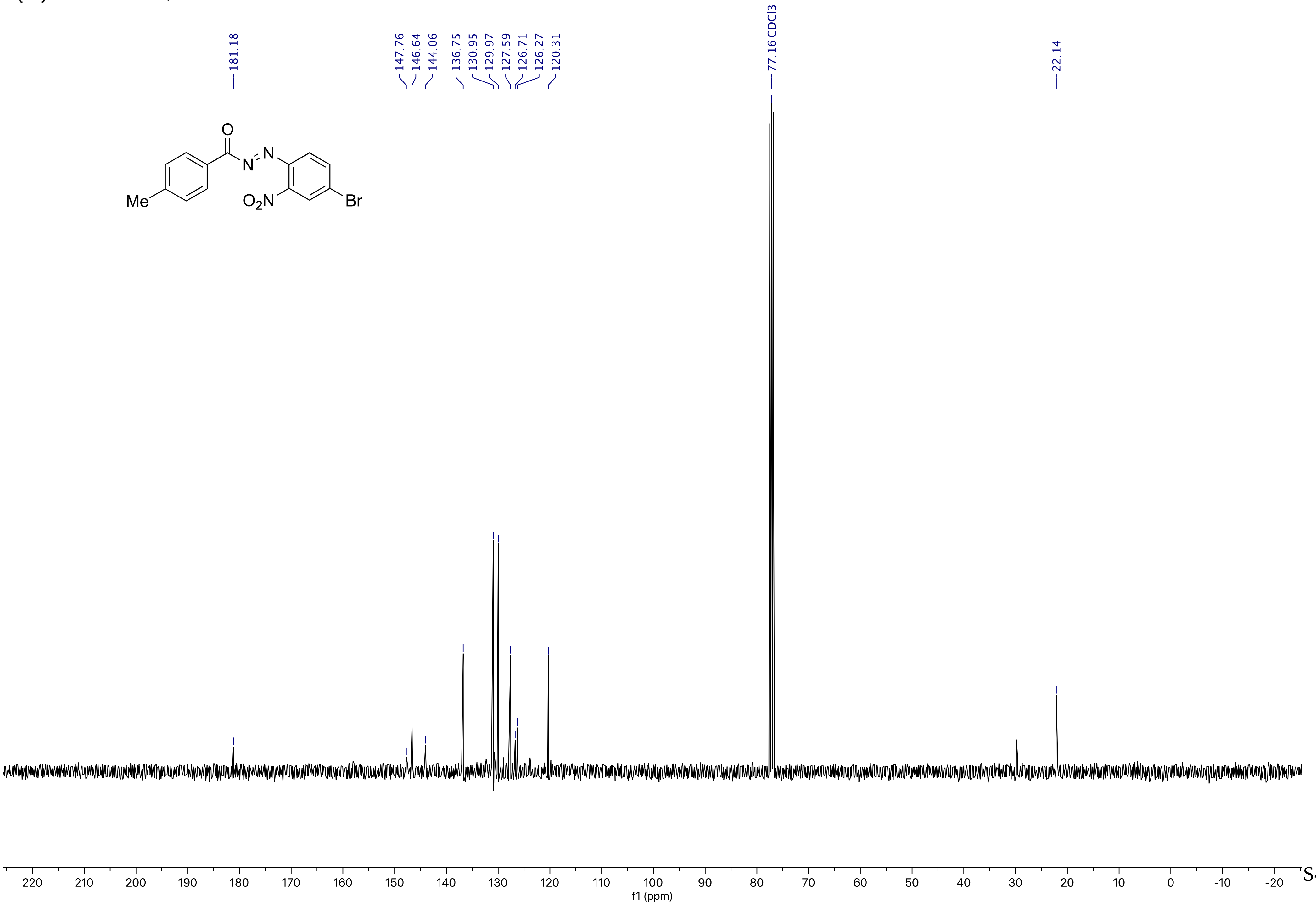

^1H NMR: 500 MHz, $CDCl_3$

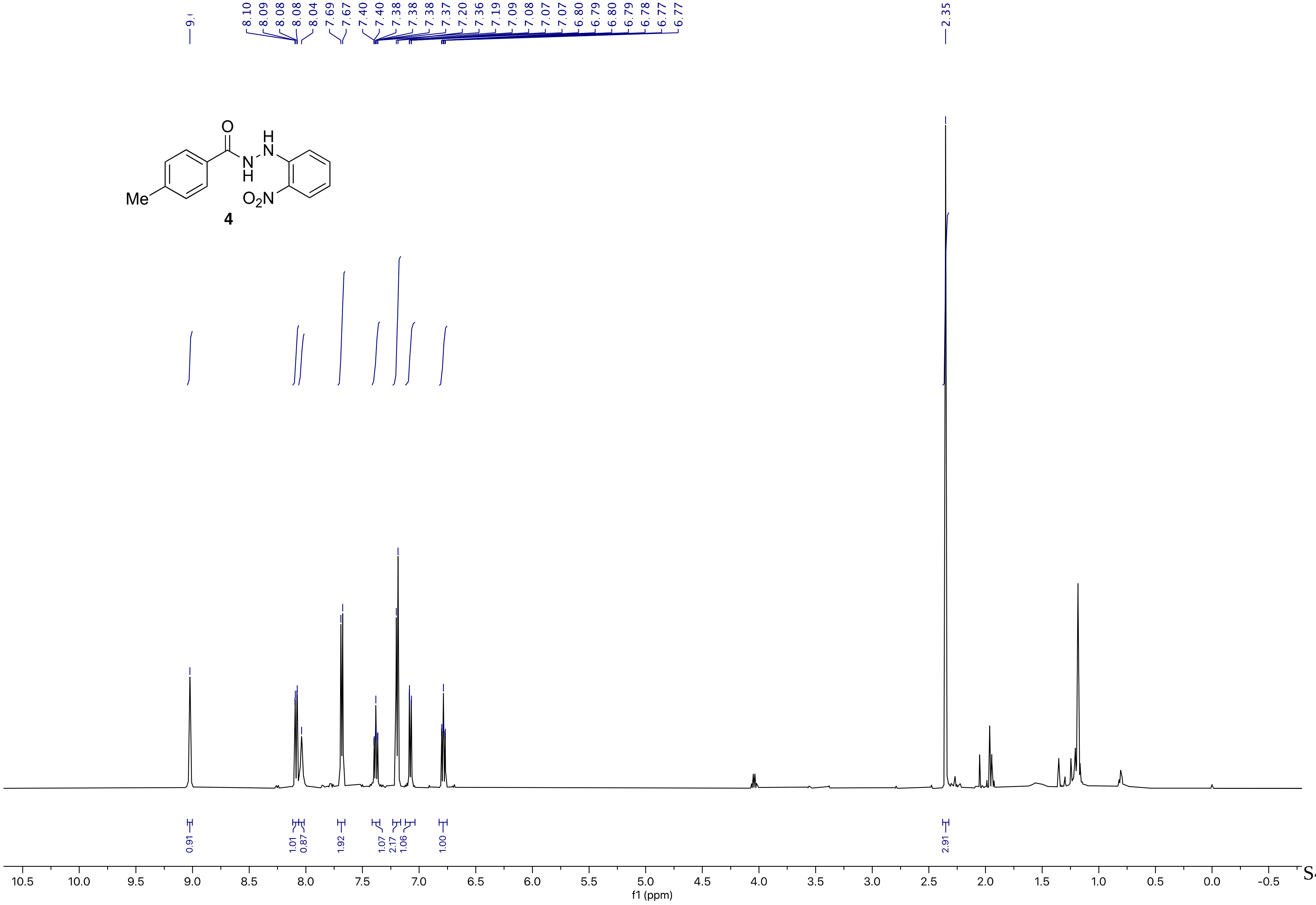

$^{13}C\{^1H\}$ NMR: 126 MHz, $CDCl_3$

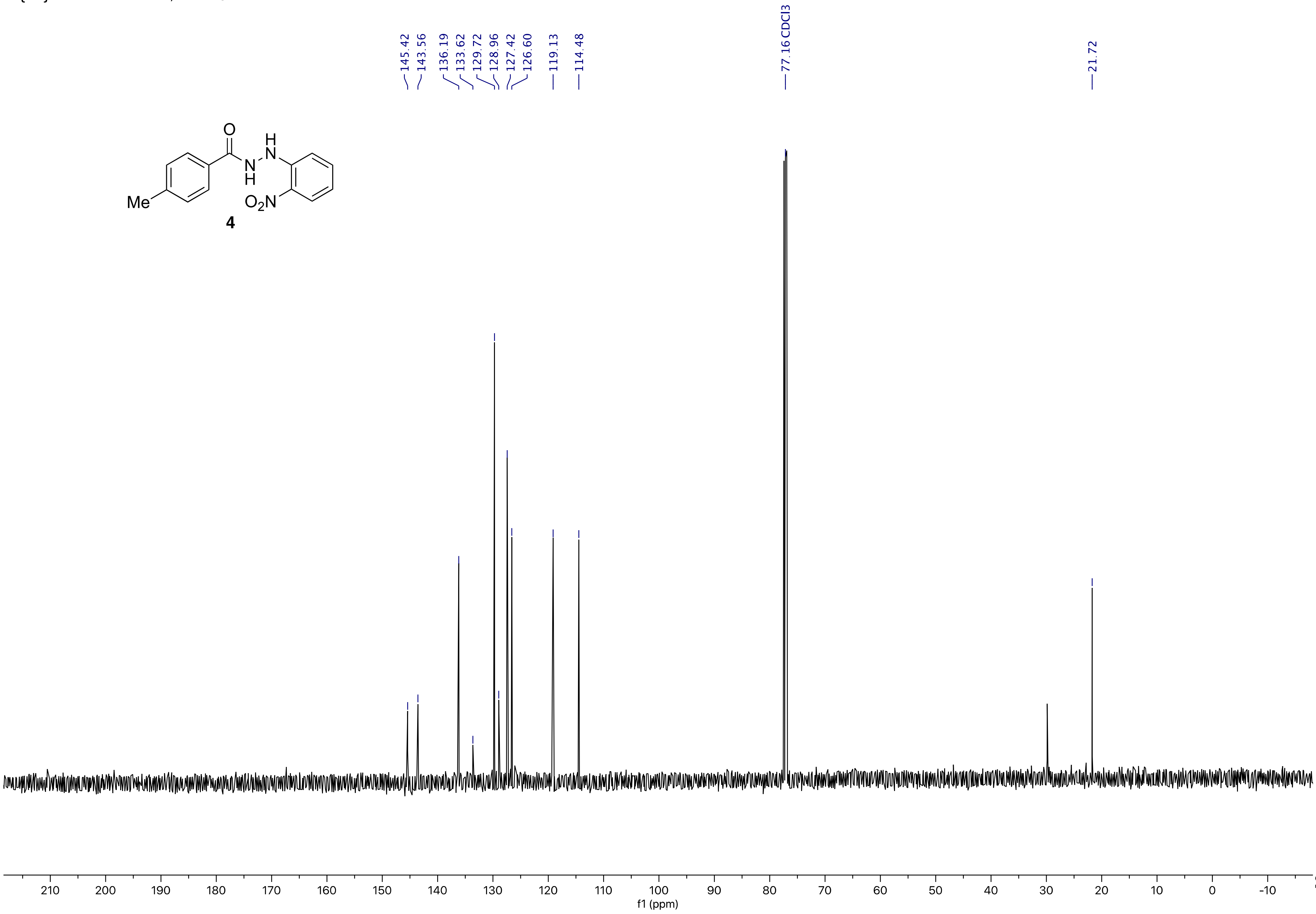

^{1}H NMR: 400 MHz, $CDCl_3$

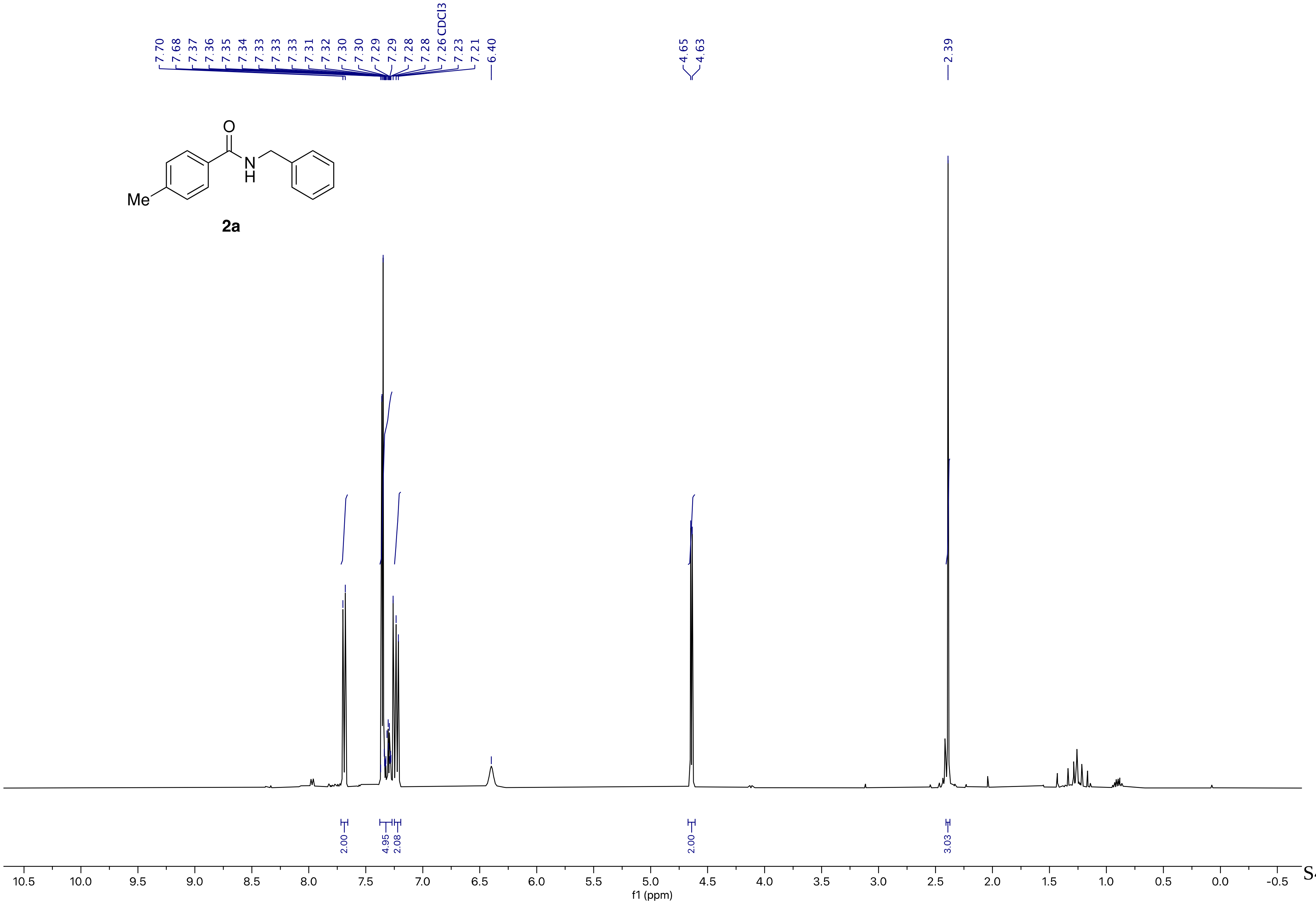

2a

$^{13}C\{^{1}H\}$ NMR: 101 MHz, $CDCl_3$

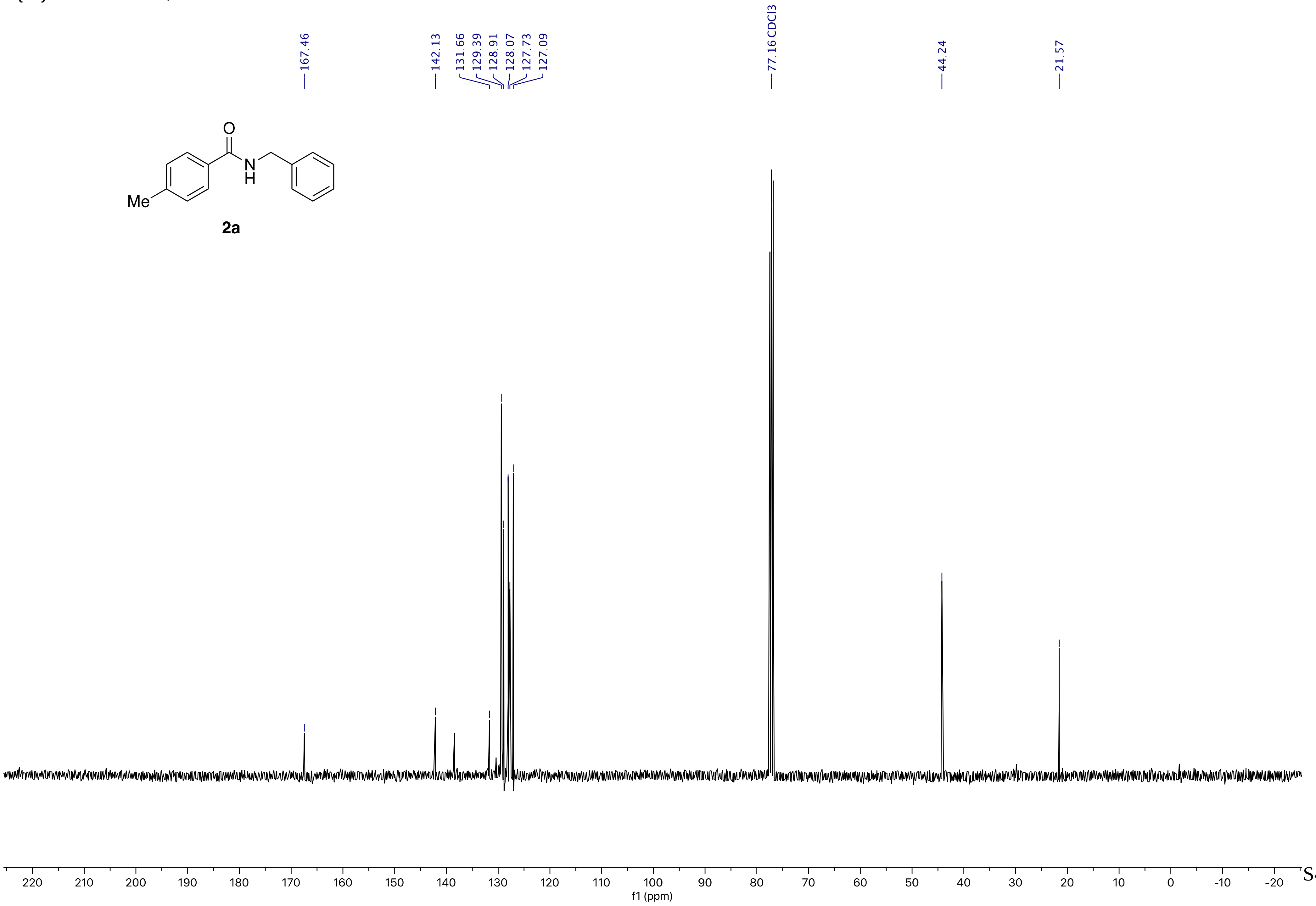

^1H NMR: 500 MHz, $CDCl_3$

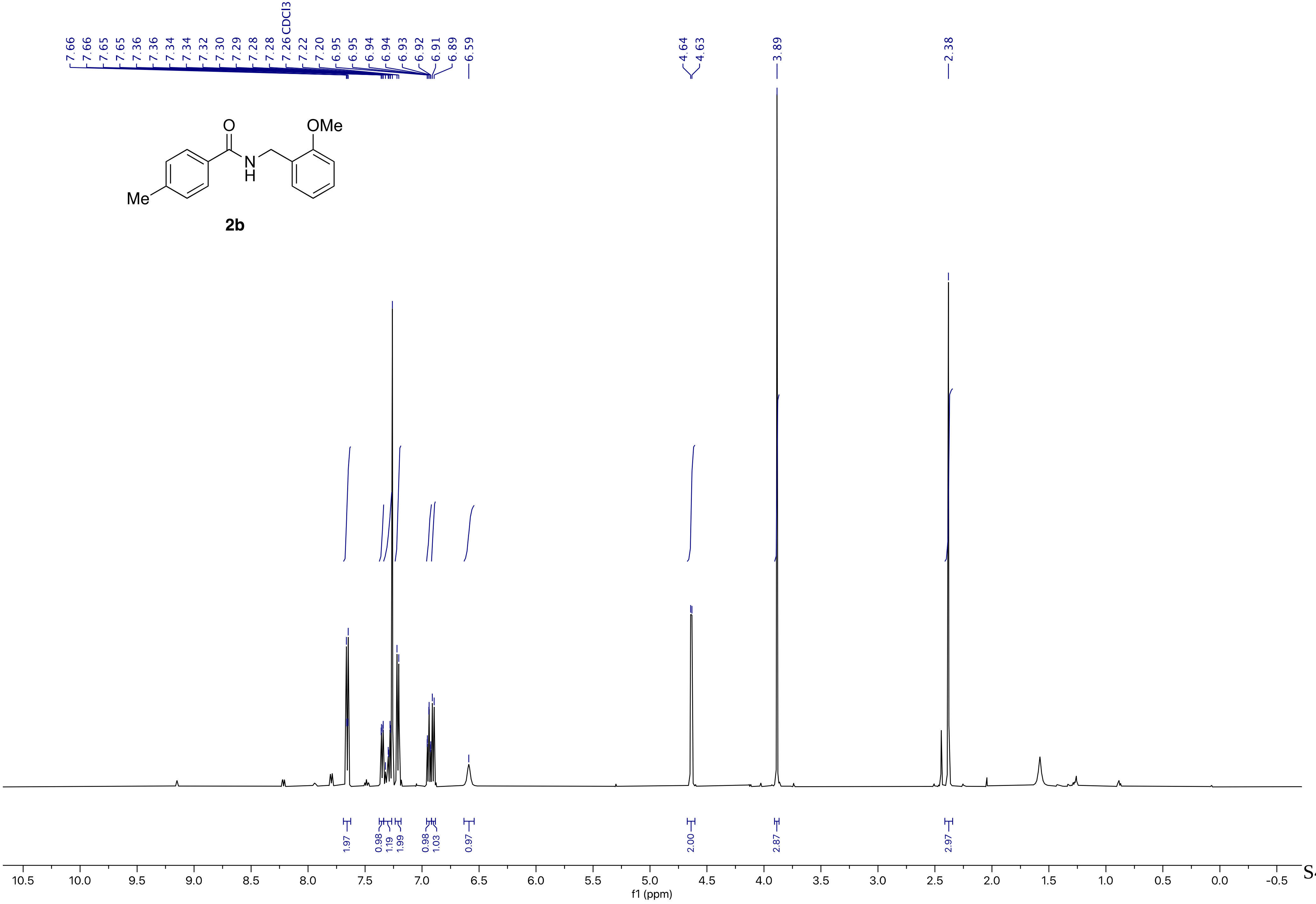

$^{13}C\{^1H\}$ NMR: 126 MHz, $CDCl_3$

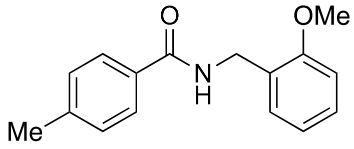

2b

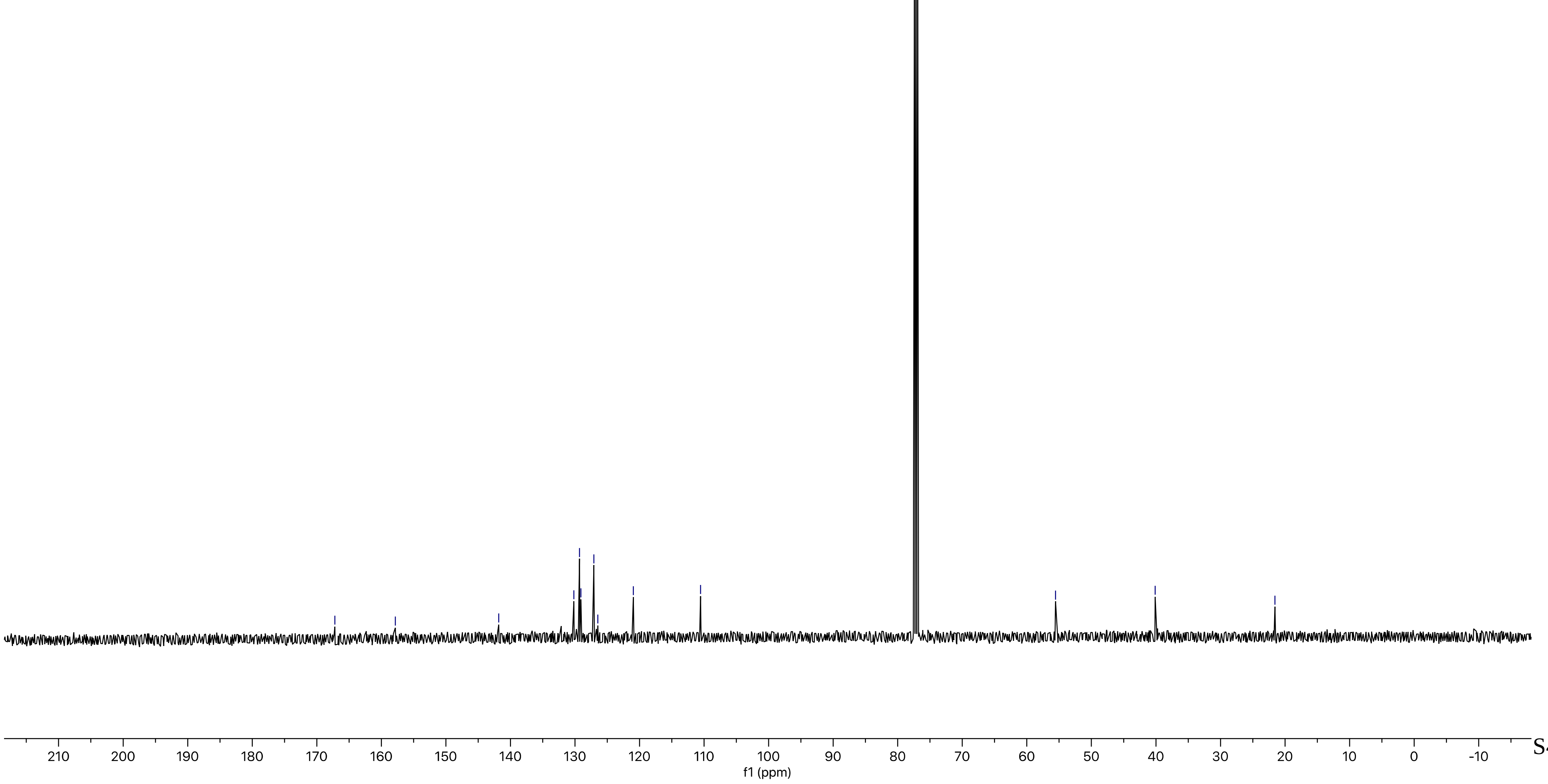

^1H NMR: 500 MHz, $CDCl_3$

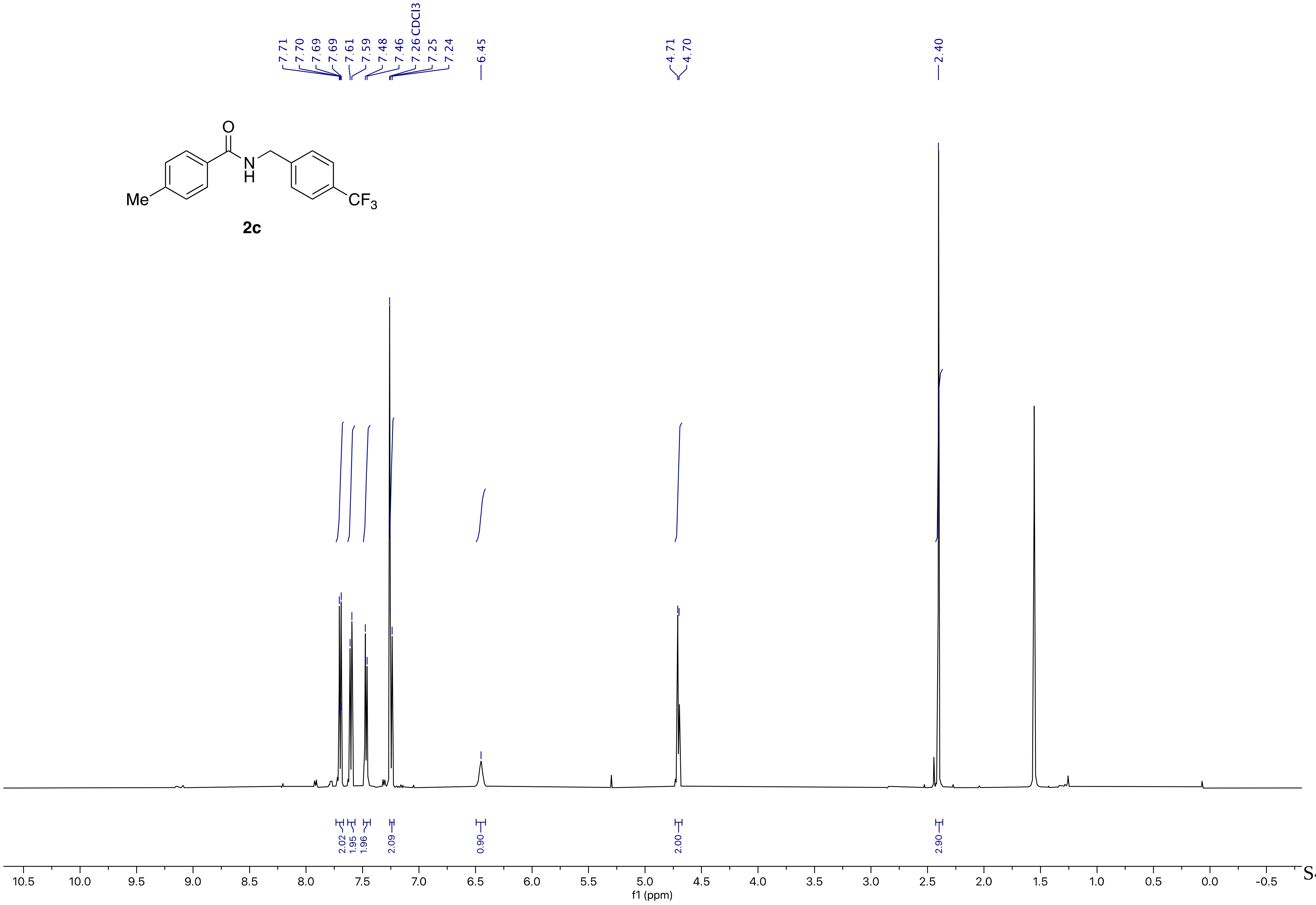

$^{13}C\{^{1}H\}$ NMR: 126 MHz, $CDCl_3$

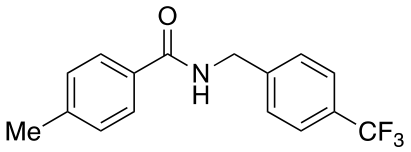

2c

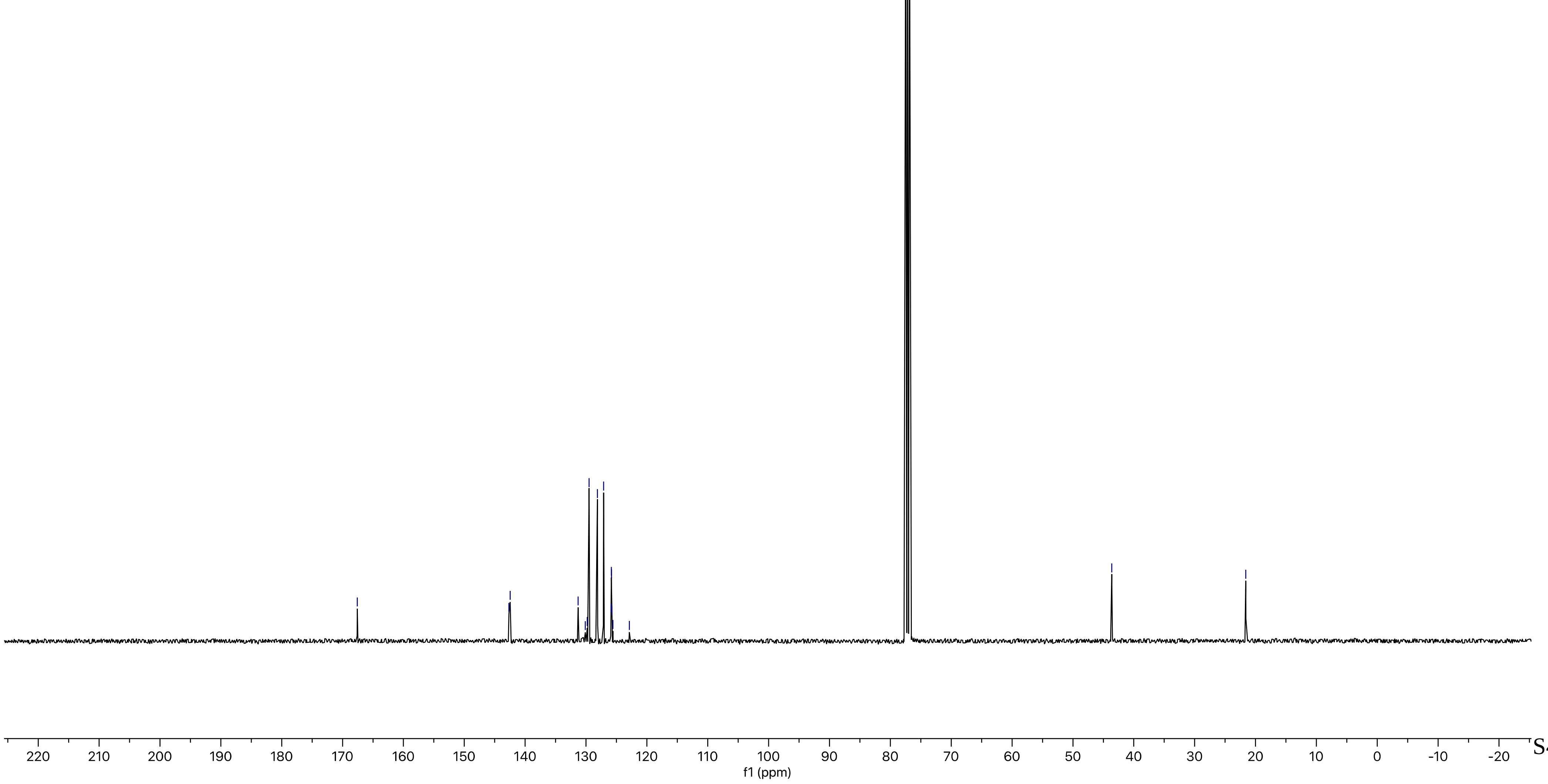

^{19}F NMR: 471 MHz, $CDCl_3$

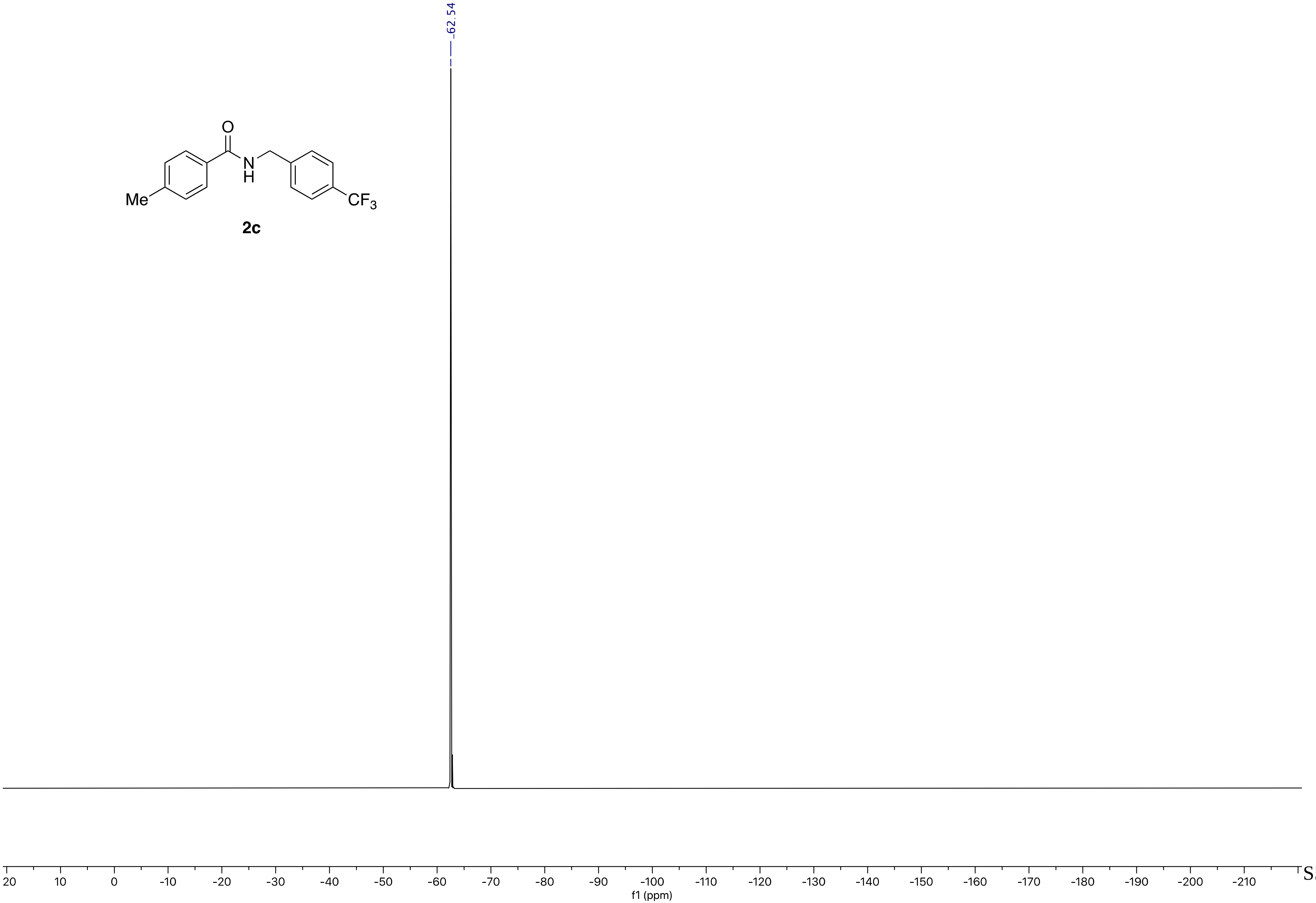

^{1}H NMR: 500 MHz, DMSO-d_6

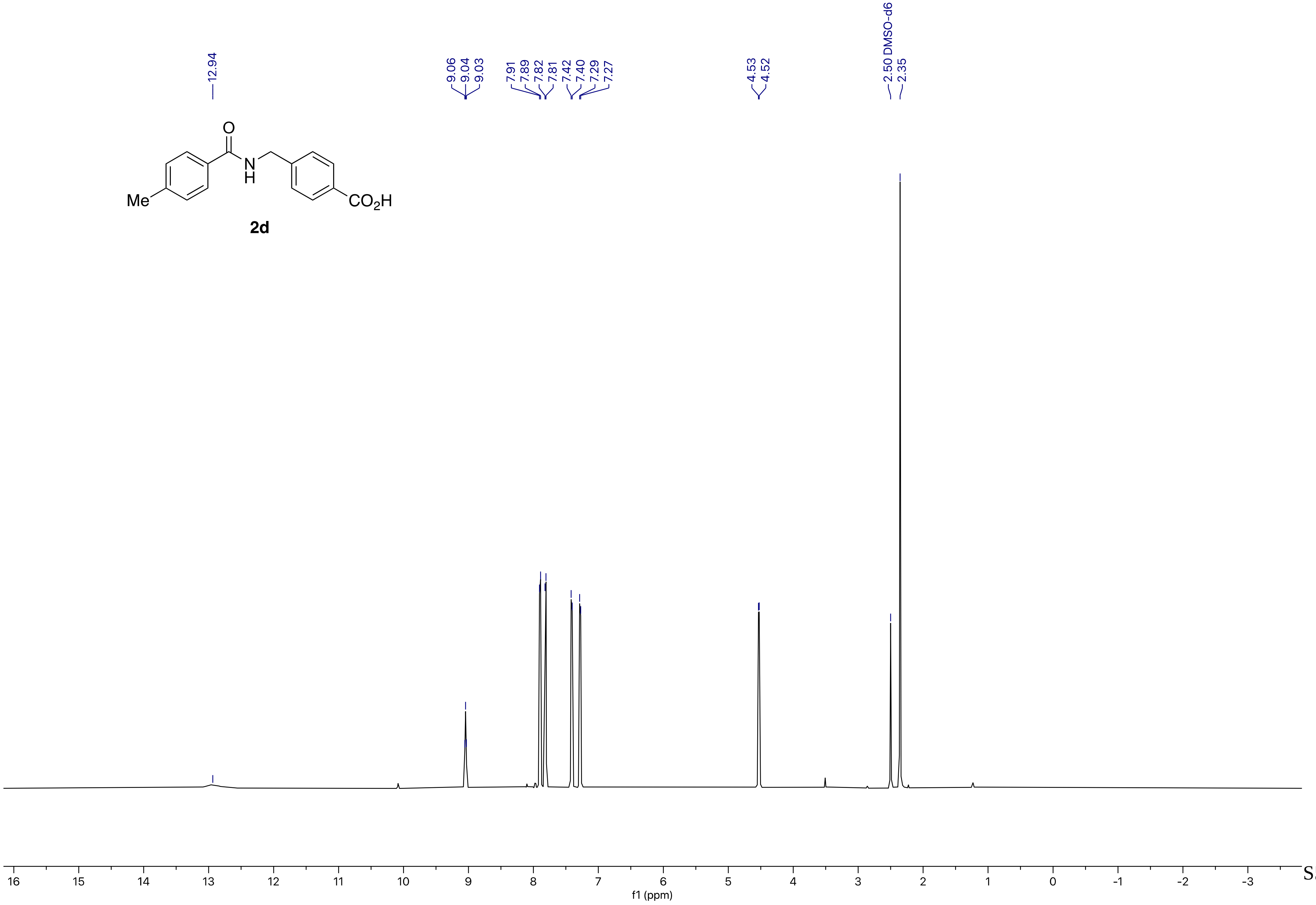

$^{13}C\{^1H\}$ NMR: 126 MHz, DMSO-d_6

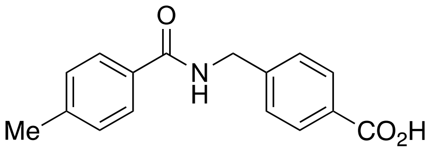

2d

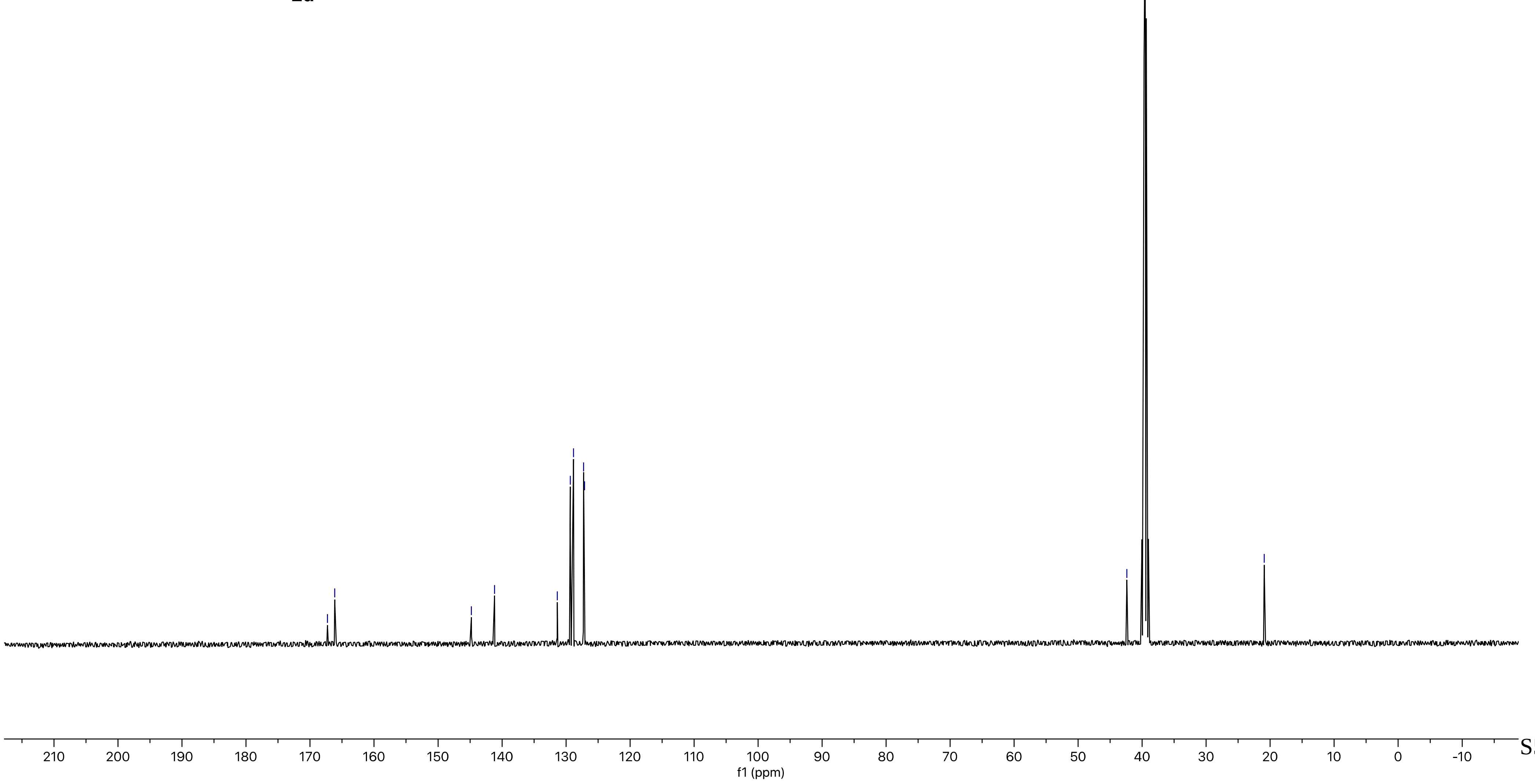

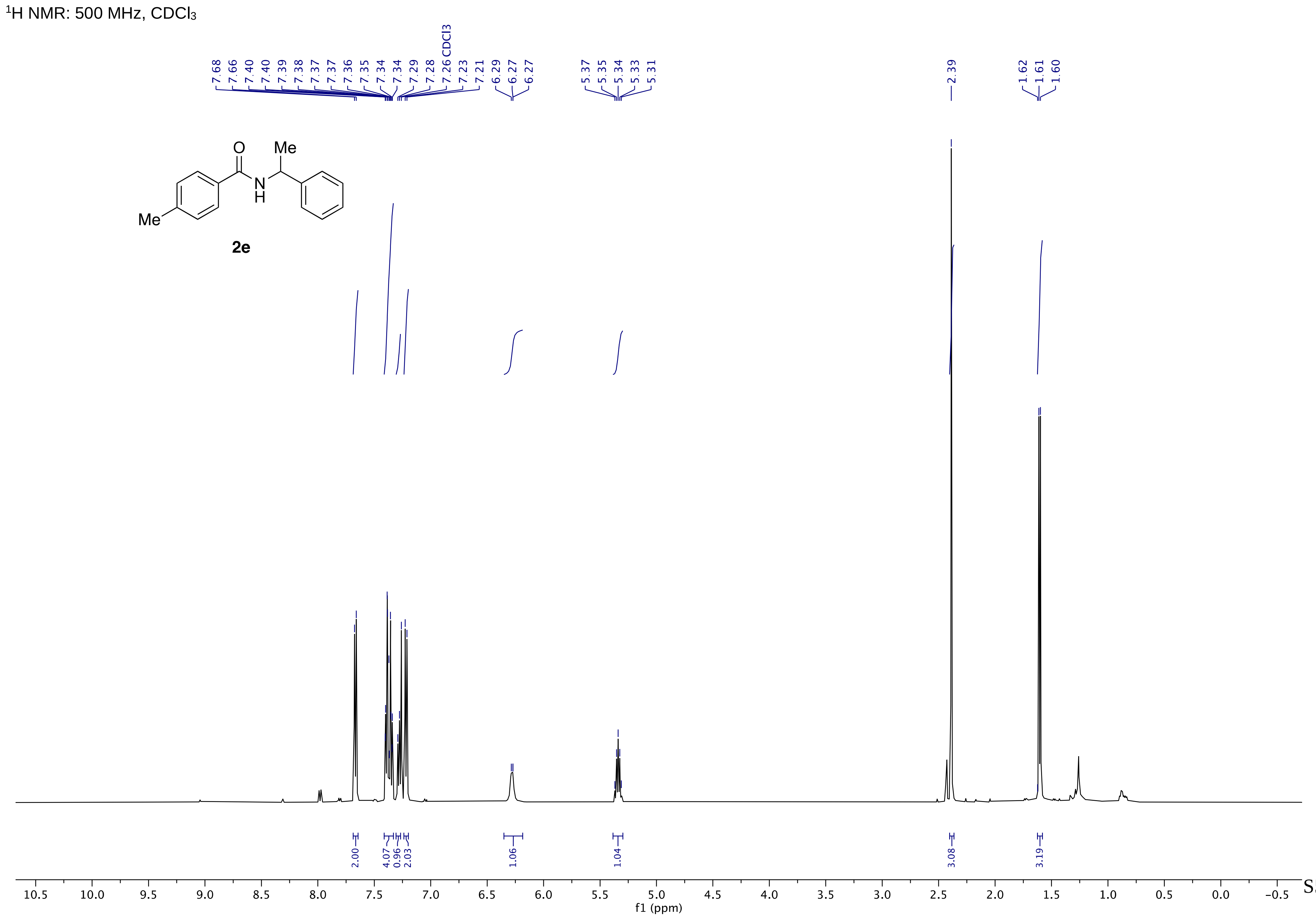

¹H NMR: 500 MHz, CDCl₃
7.68
7.66
7.40
7.40
7.39
7.38
7.37
7.37
7.36
7.35
7.34
7.34
7.29
7.28
7.26 CDCl3
7.23
7.21
6.29
6.27
6.27
5.37
5.35
5.34
5.33
5.31
2.39
1.62
1.61
1.60
O
Me
N
H
Me
2e
2.00
4.07
0.96
2.03
1.06
1.04
3.08
3.19
10.5 10.0 9.5 9.0 8.5 8.0 7.5 7.0 6.5 6.0 5.5 5.0 4.5 4.0 3.5 3.0 2.5 2.0 1.5 1.0 0.5 0.0 −0.5
f1 (ppm)

$^{13}C\{^{1}H\}$ NMR: 126 MHz, $CDCl_3$

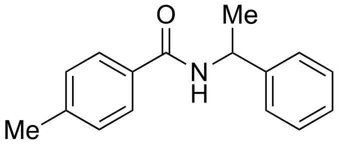

2e

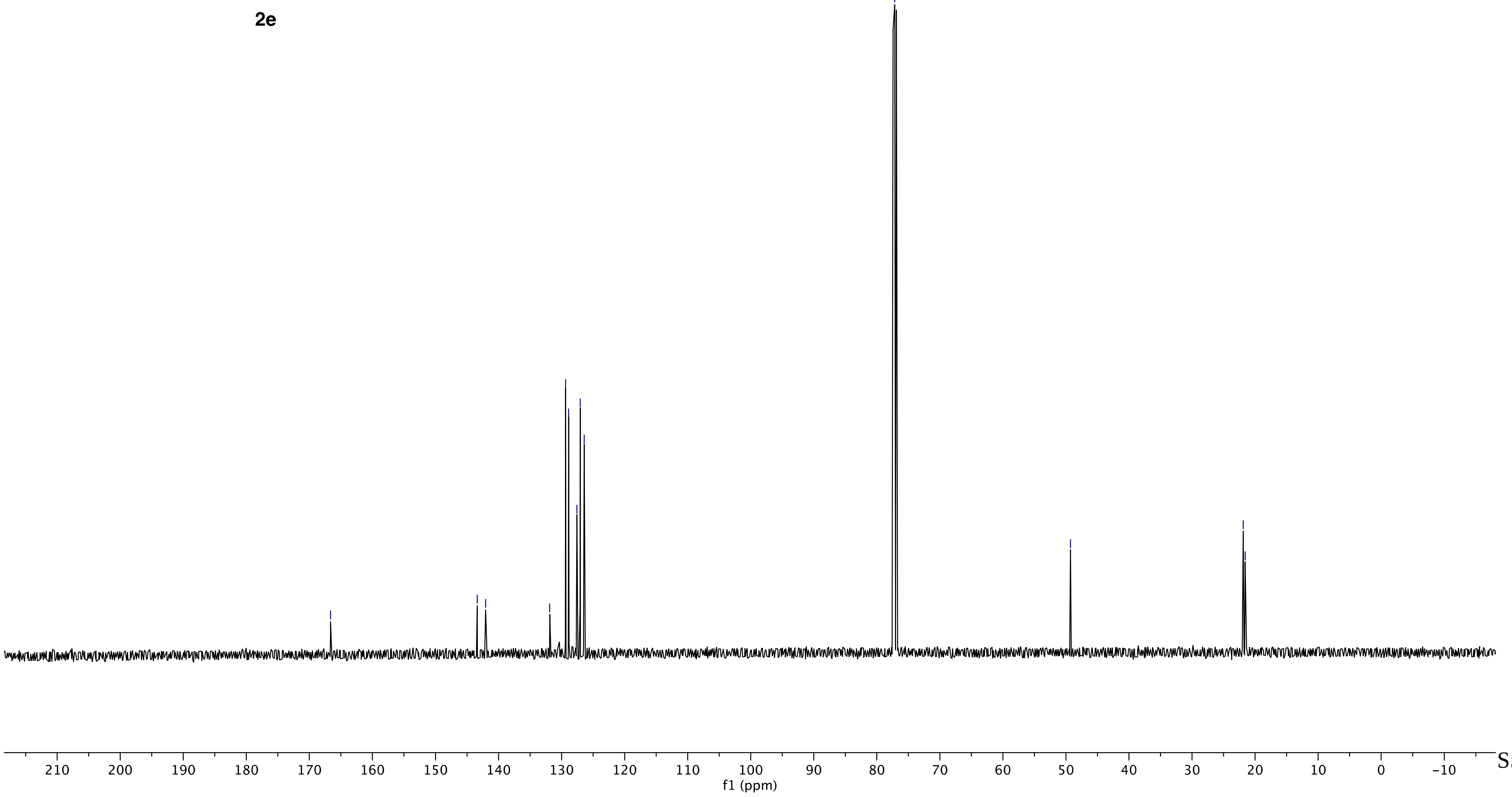

^1H NMR: 500 MHz, $CDCl_3$

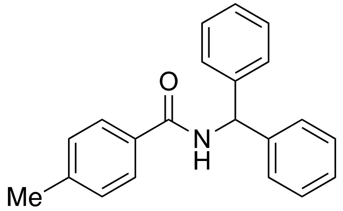

2f

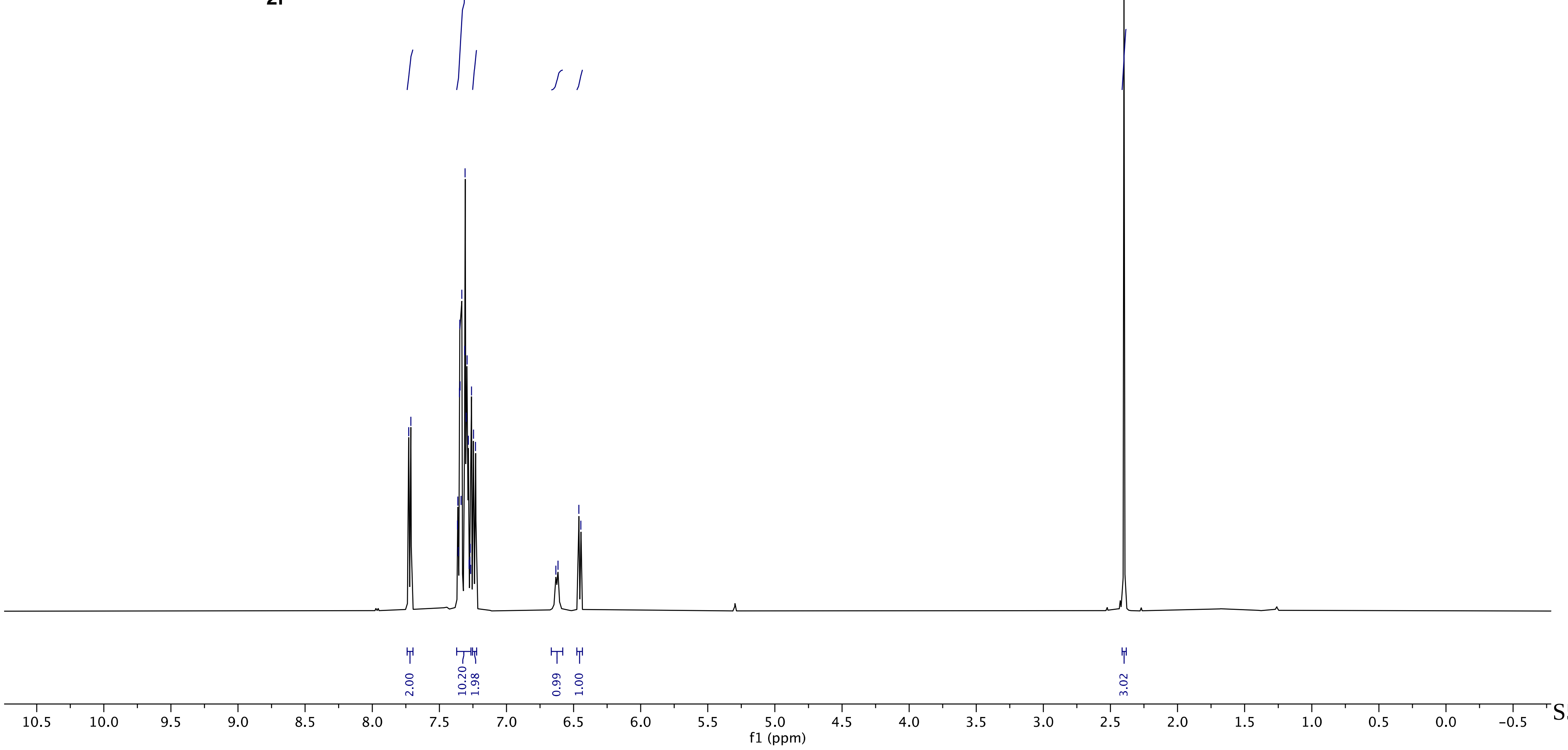

$^{13}C\{^1H\}$ NMR: 126 MHz, $CDCl_3$

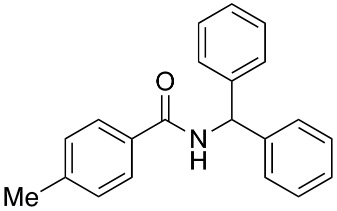

2f

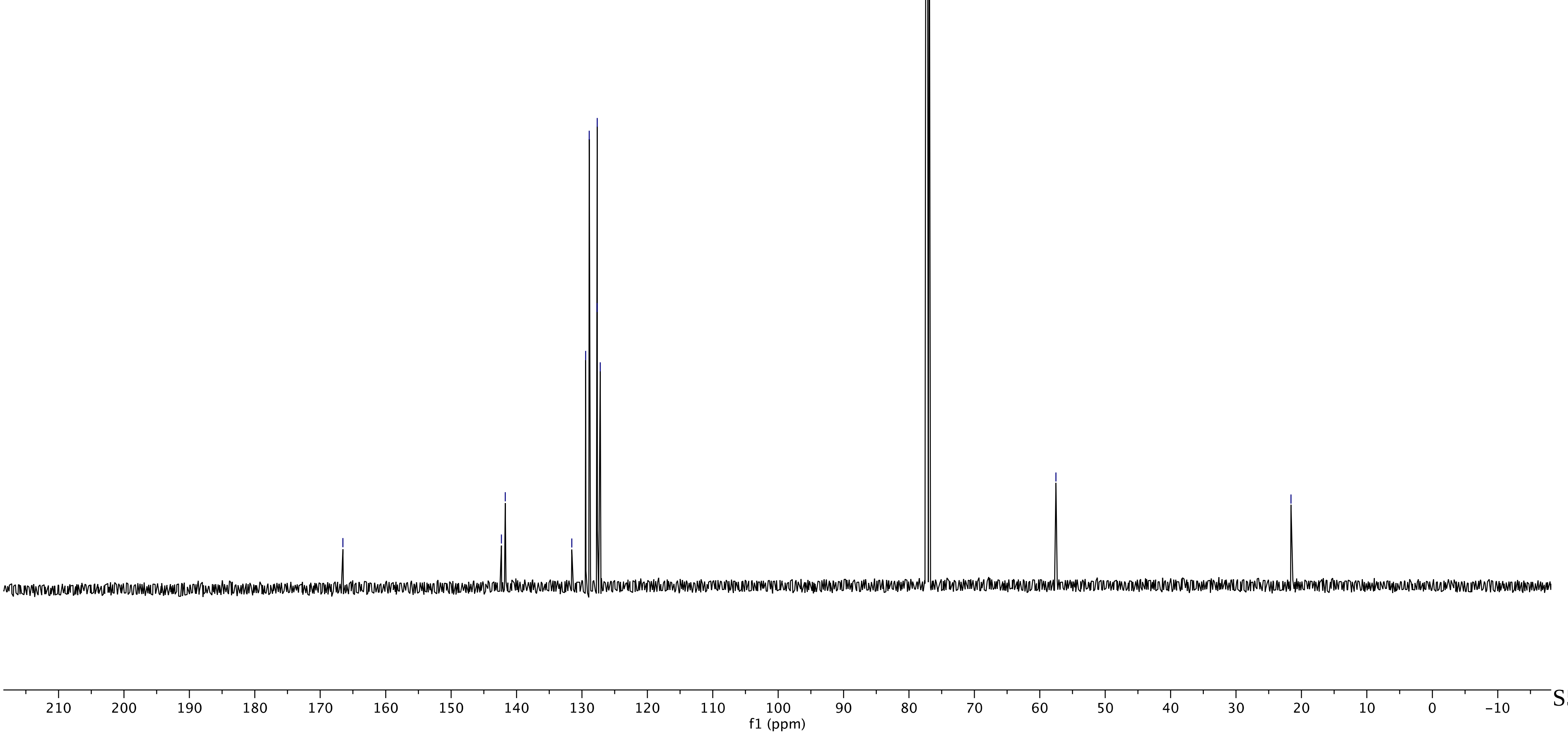

^{1}H NMR: 500 MHz, $CDCl_3$

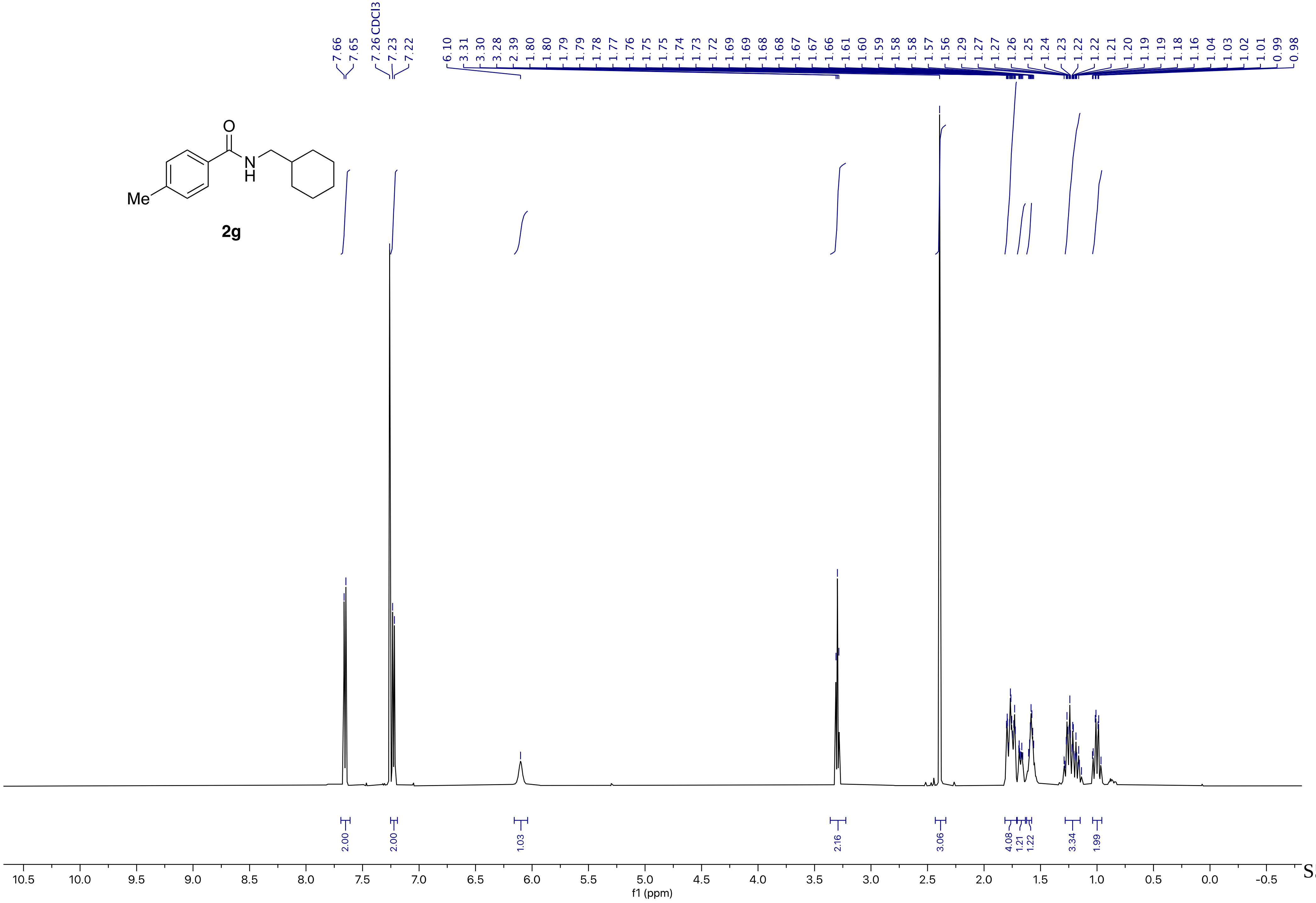

$^{13}C\{^1H\}$ NMR: 126 MHz, $CDCl_3$

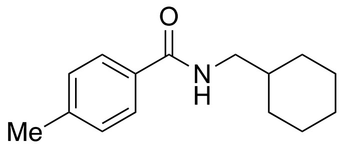

2g

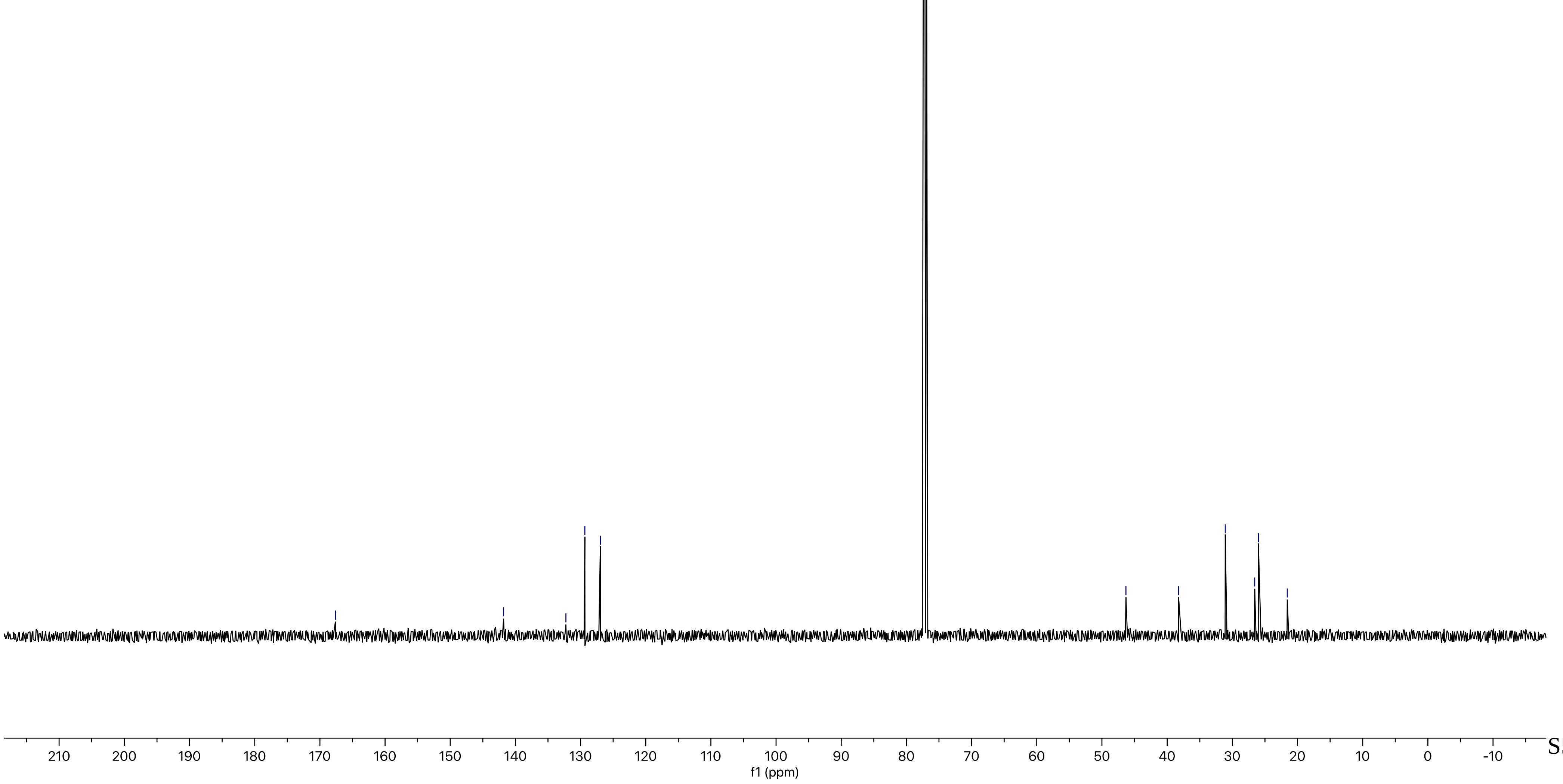

^{1}H NMR: 500 MHz, $CDCl_3$

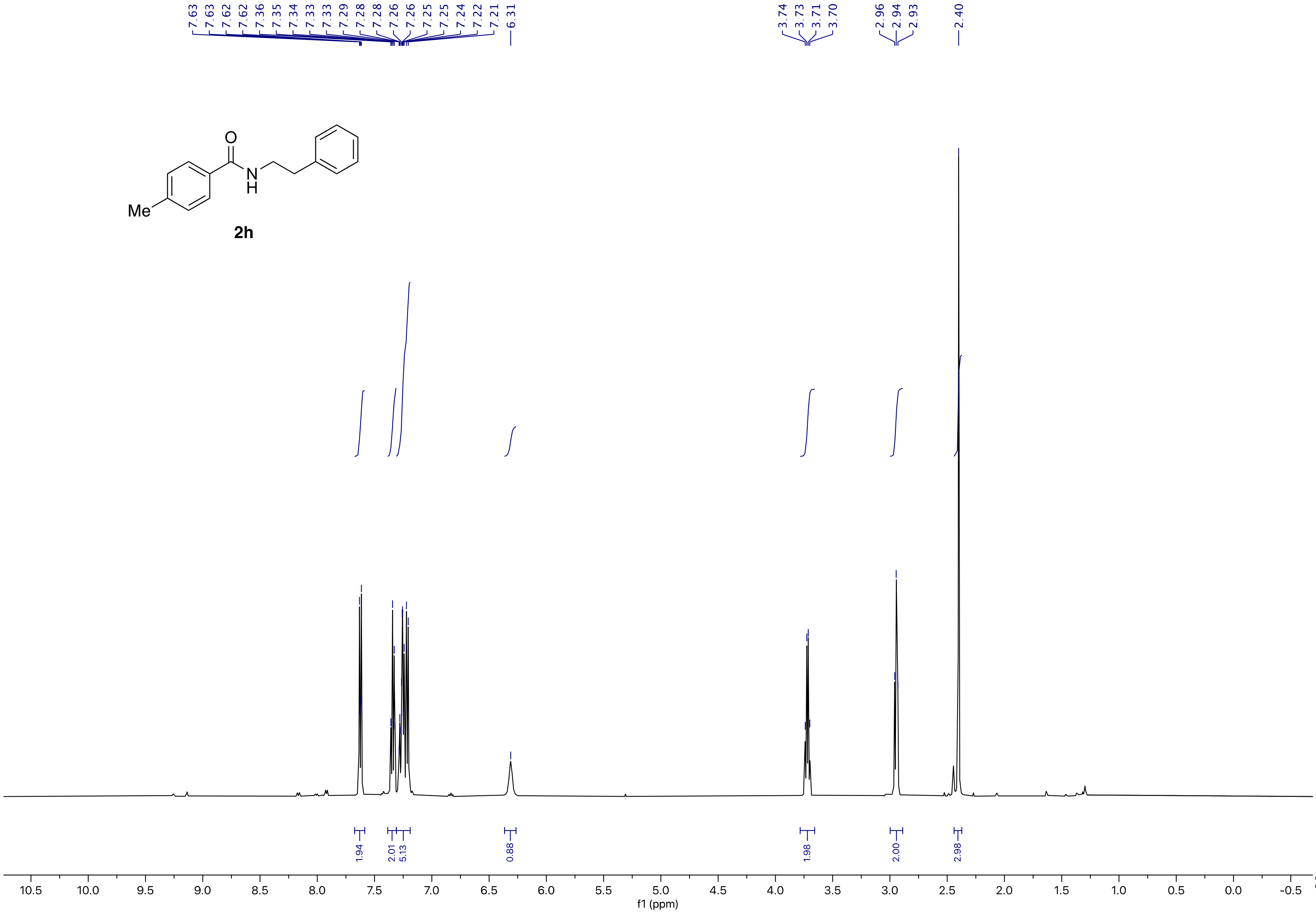

$^{13}C\{^1H\}$ NMR: 126 MHz, $CDCl_3$

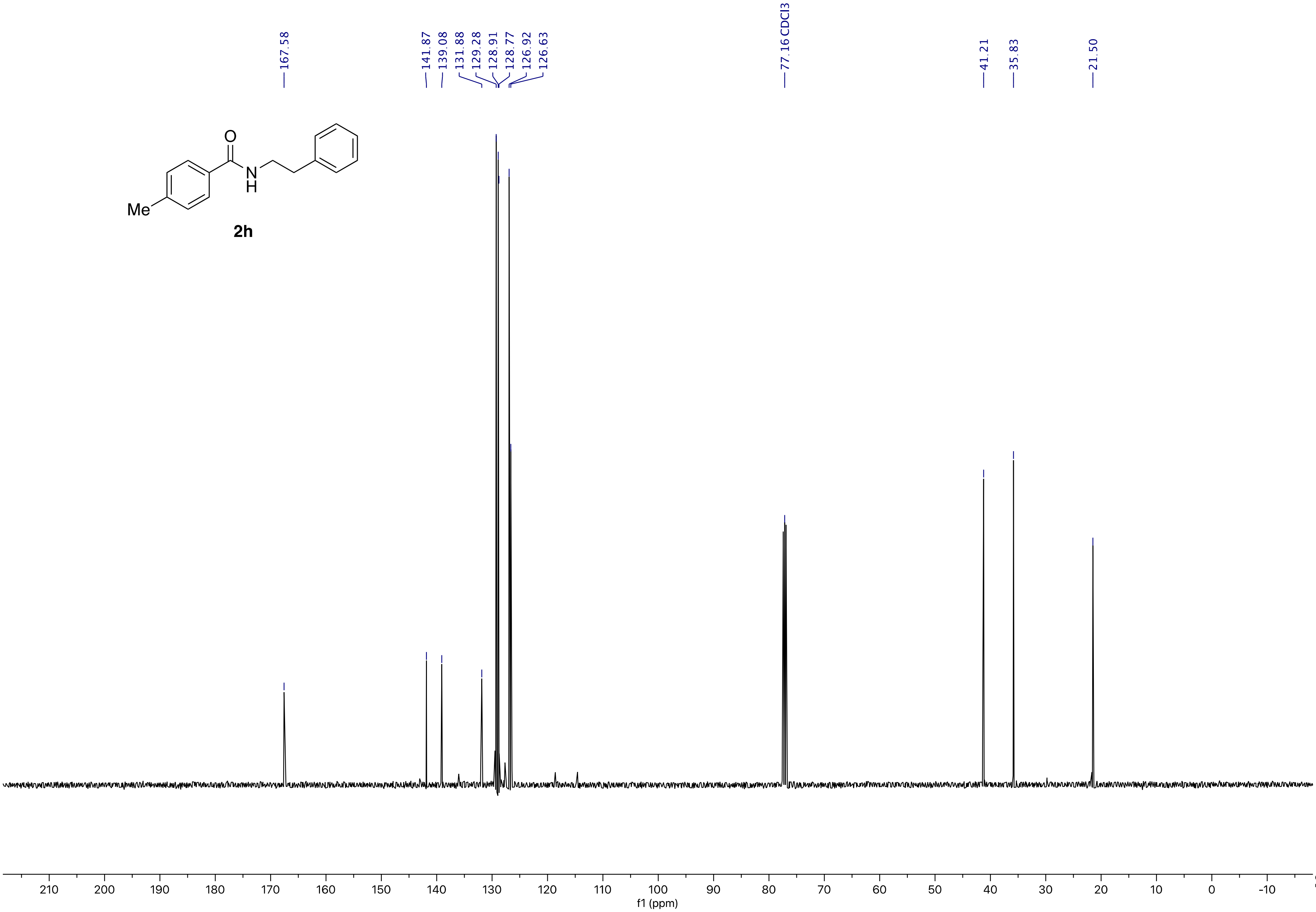

2h

^{1}H NMR: 500 MHz, $CDCl_3$

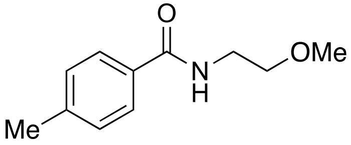

2i

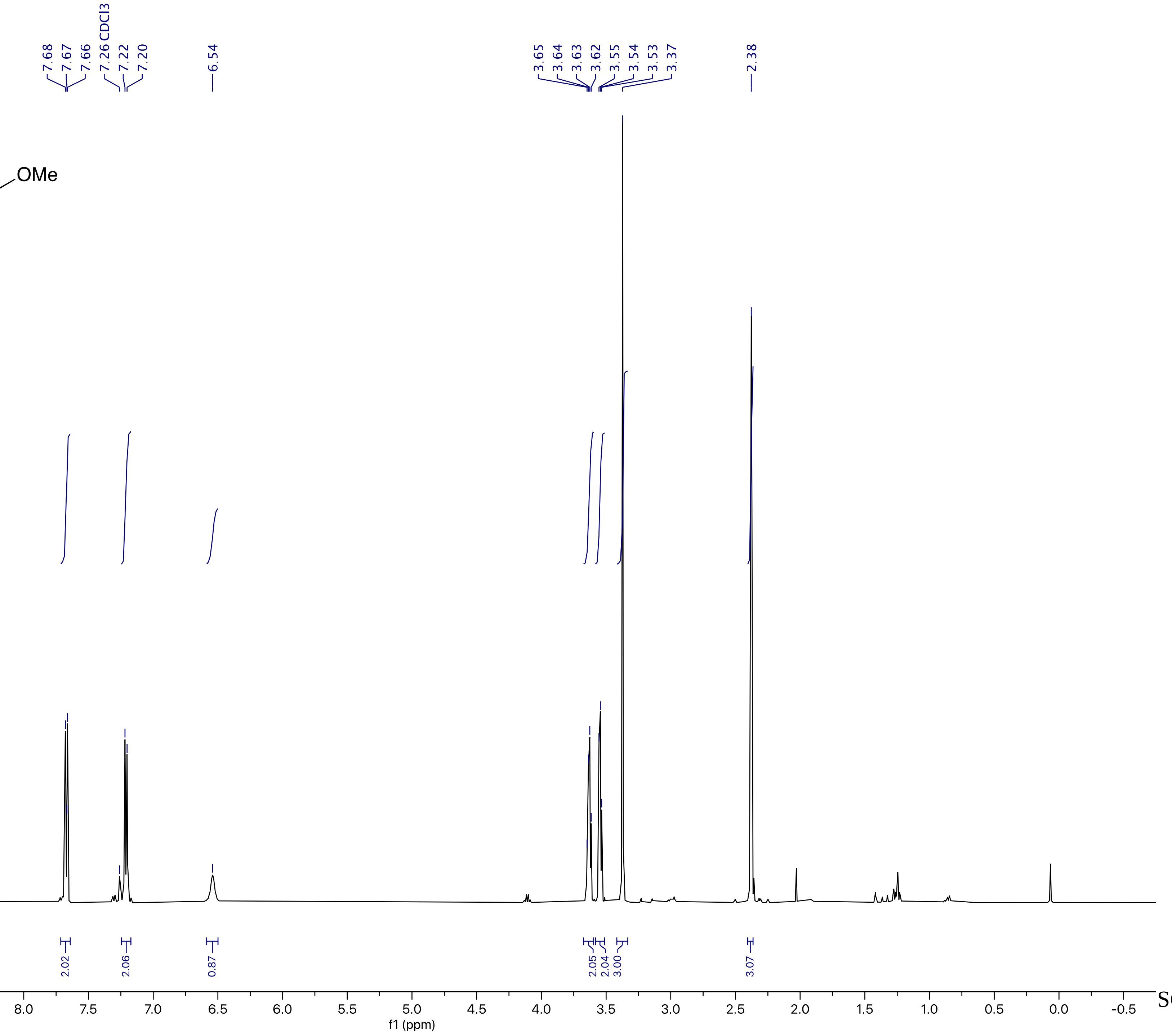

$^{13}C\{^1H\}$ NMR: 126 MHz, $CDCl_3$

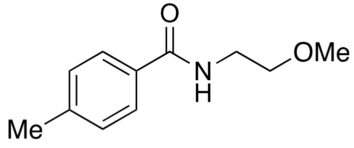

2i

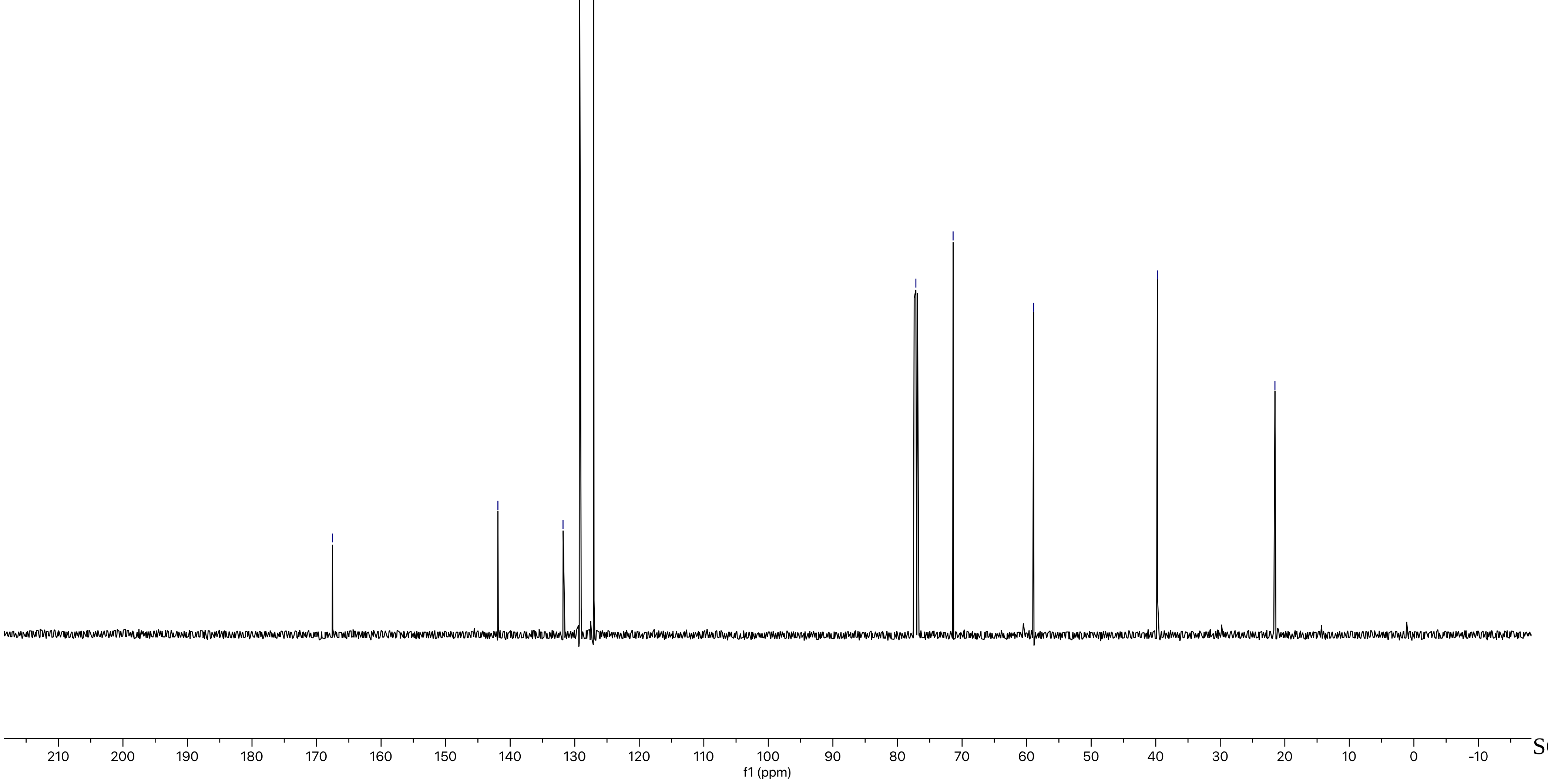

^{1}H NMR: 400 MHz, $CDCl_3$

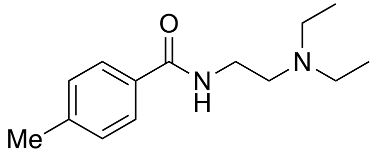

2j

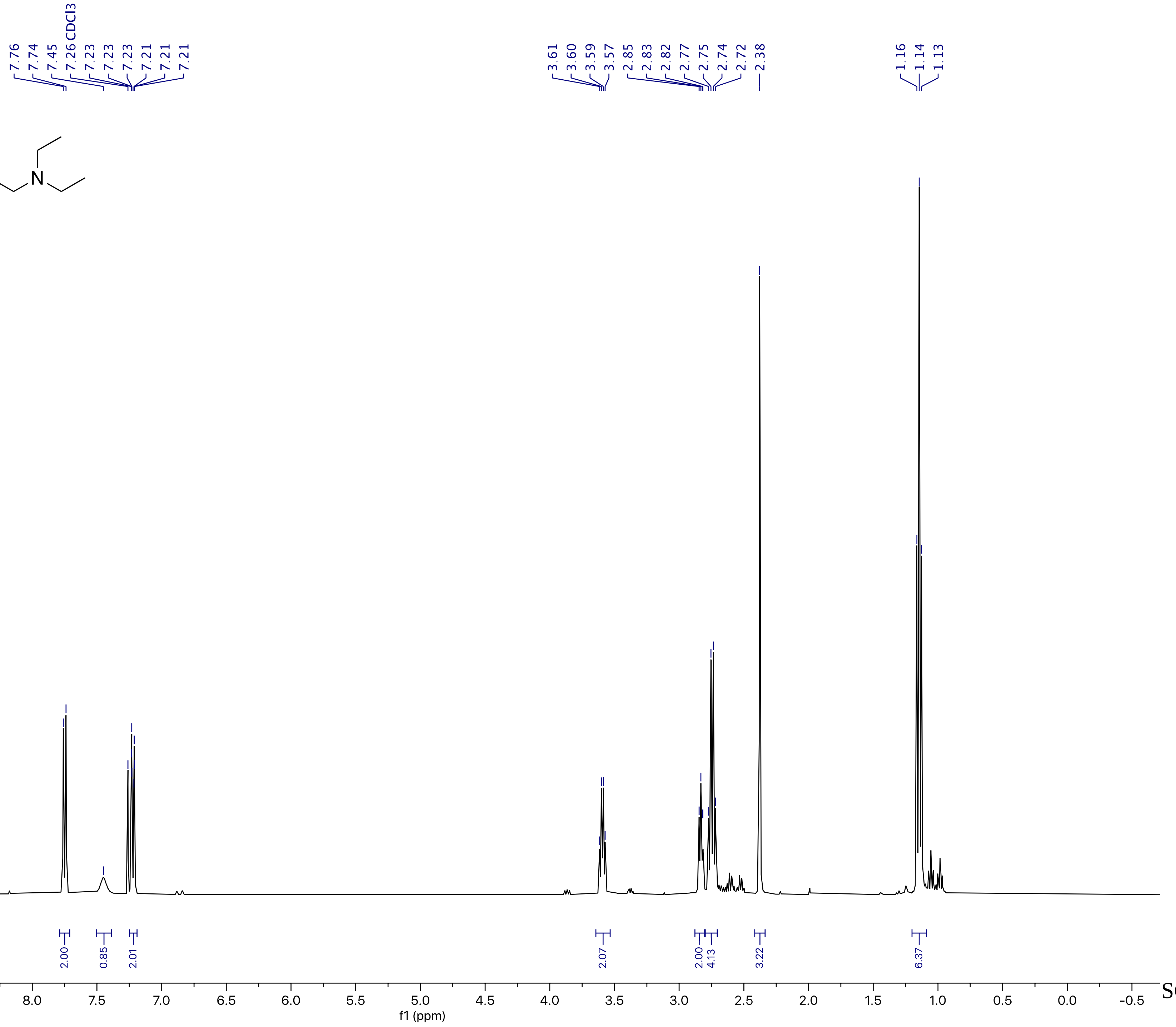

$^{13}C\{^1H\}$ NMR: 101 MHz, $CDCl_3$

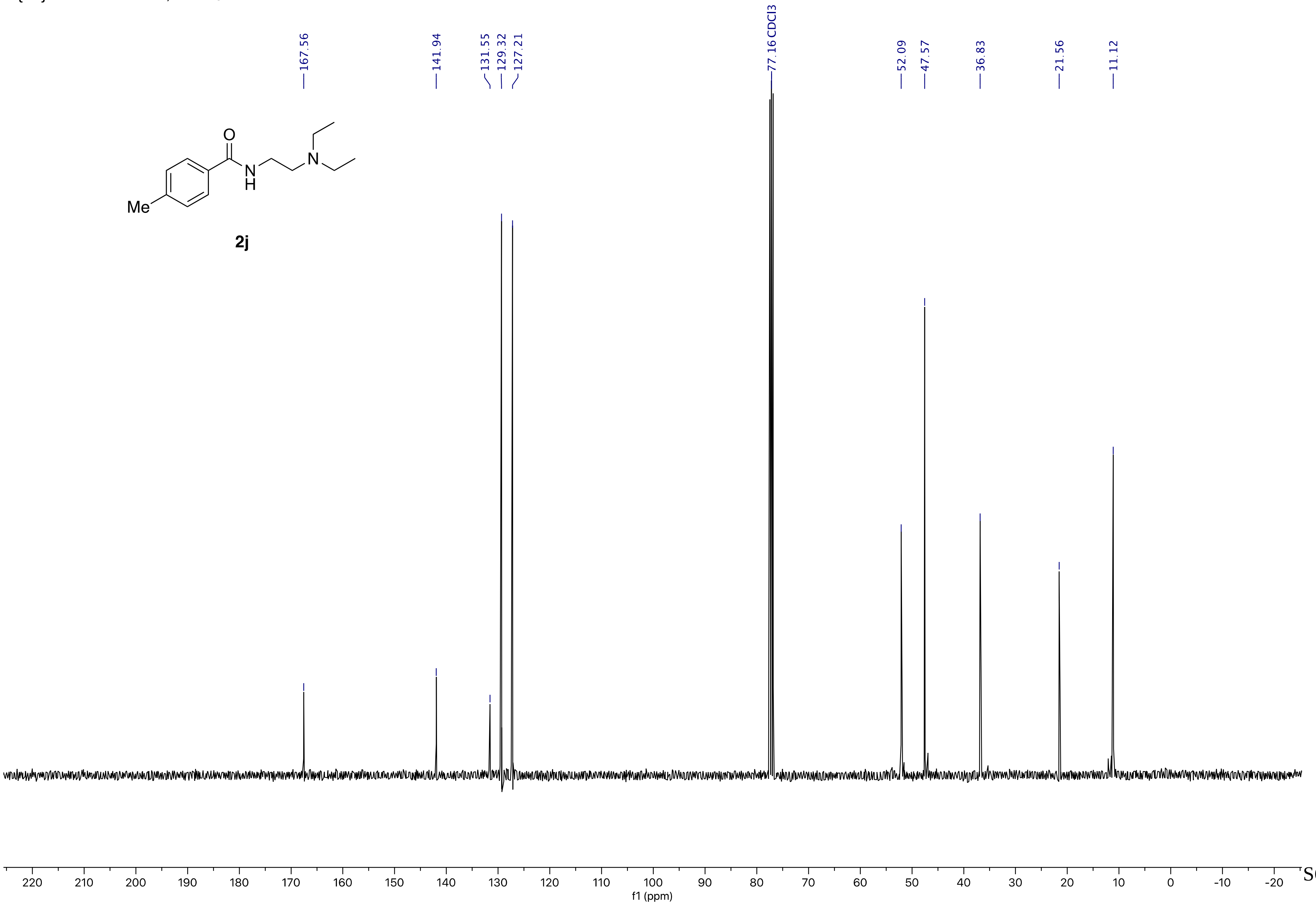

^{1}H NMR: 400 MHz, $CDCl_3$

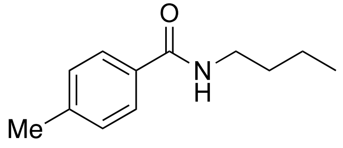

2k

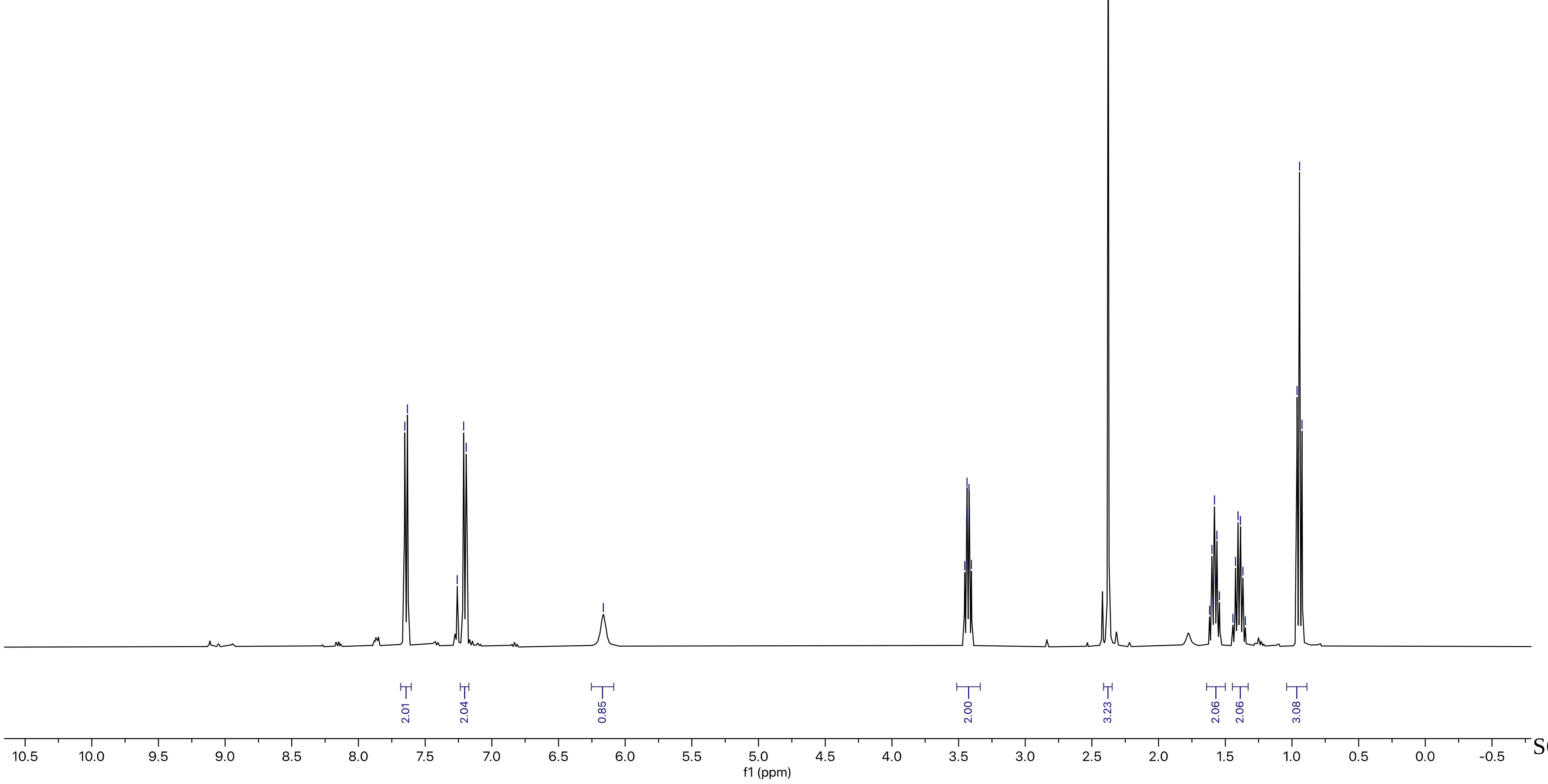

$^{13}C\{^{1}H\}$ NMR: 101 MHz, $CDCl_3$

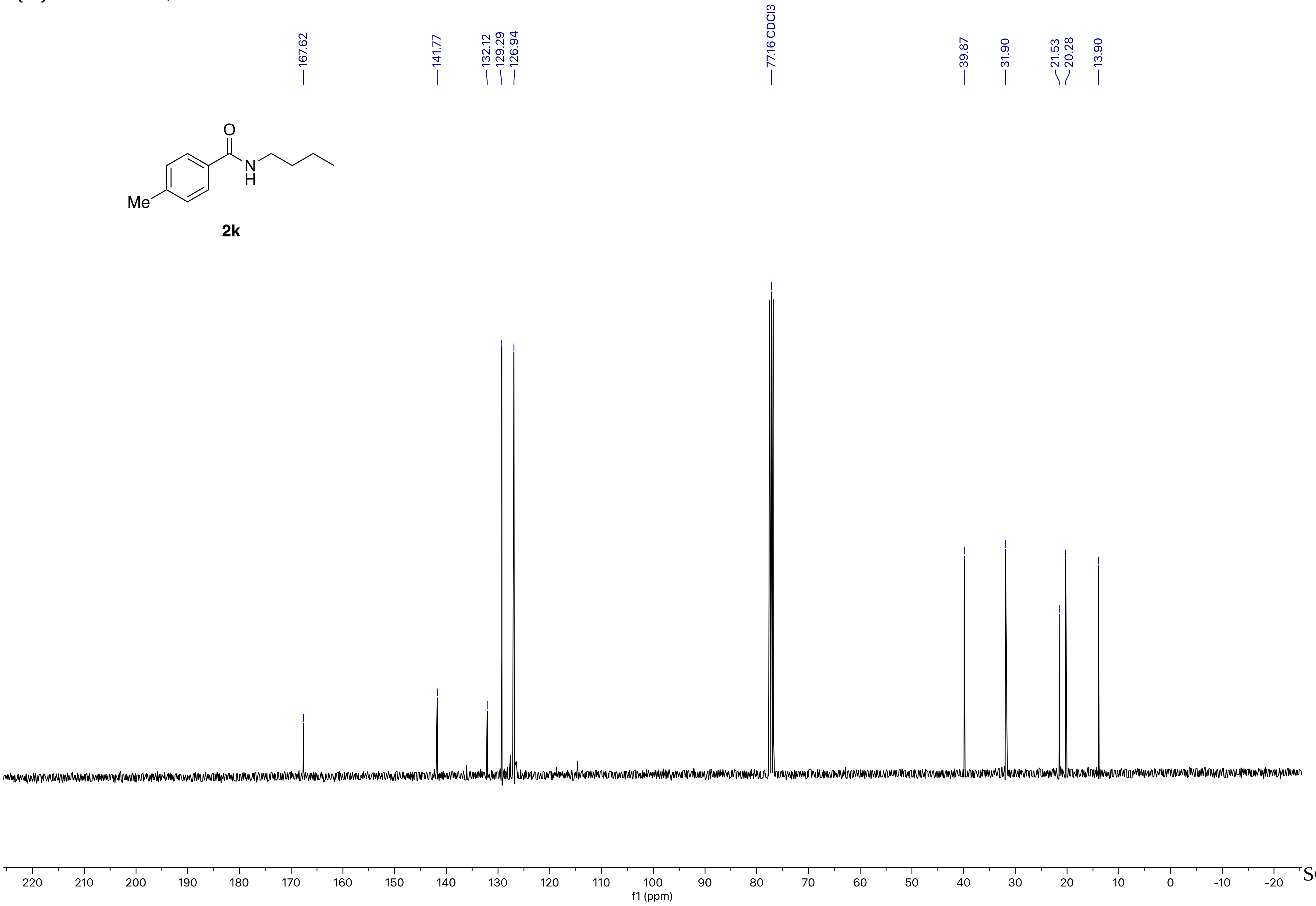

^{1}H NMR: 400 MHz, $CDCl_3$

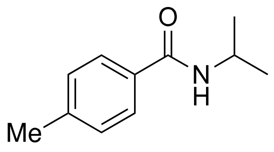

2l

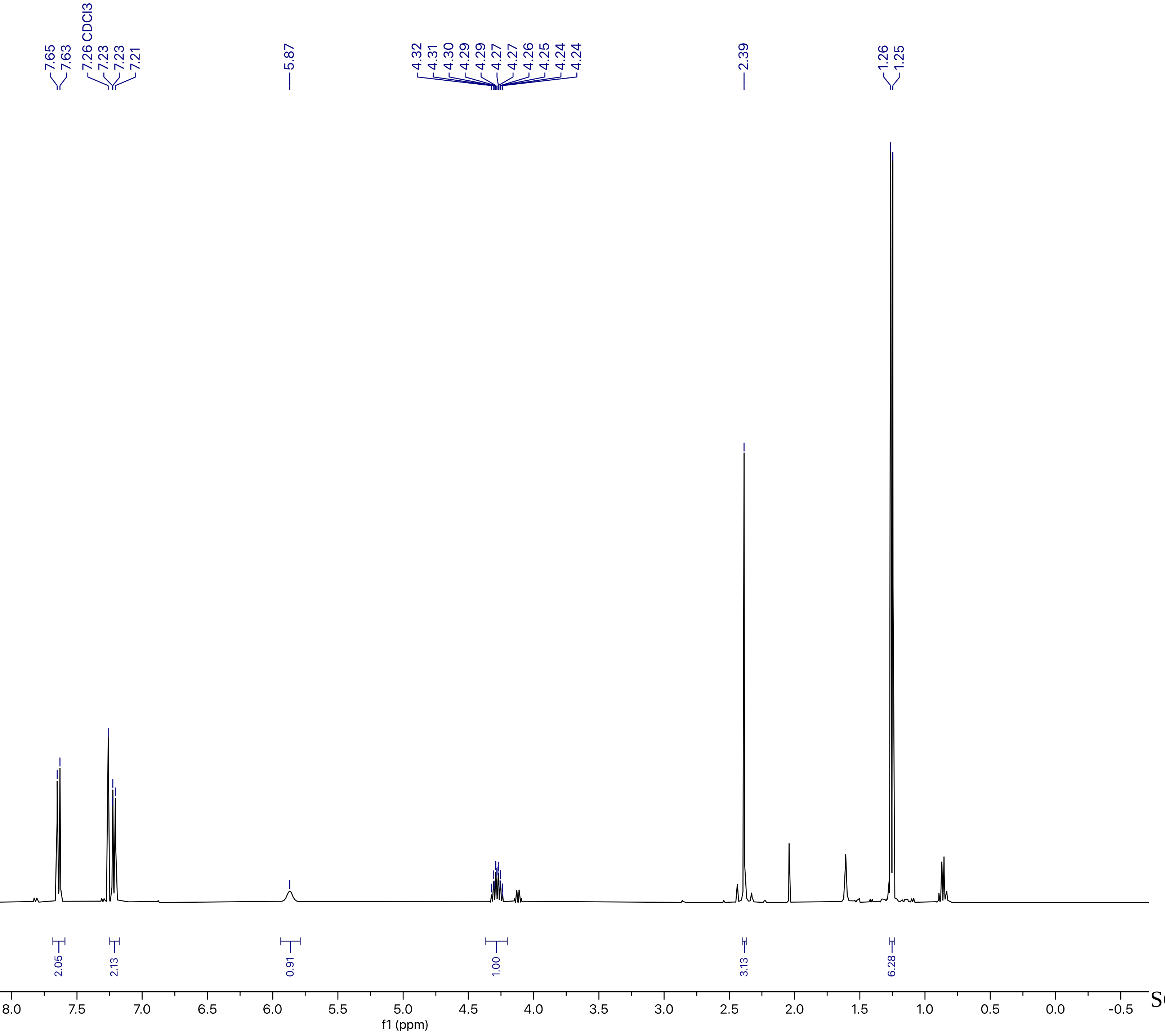

$^{13}C\{^1H\}$ NMR: 101 MHz, $CDCl_3$

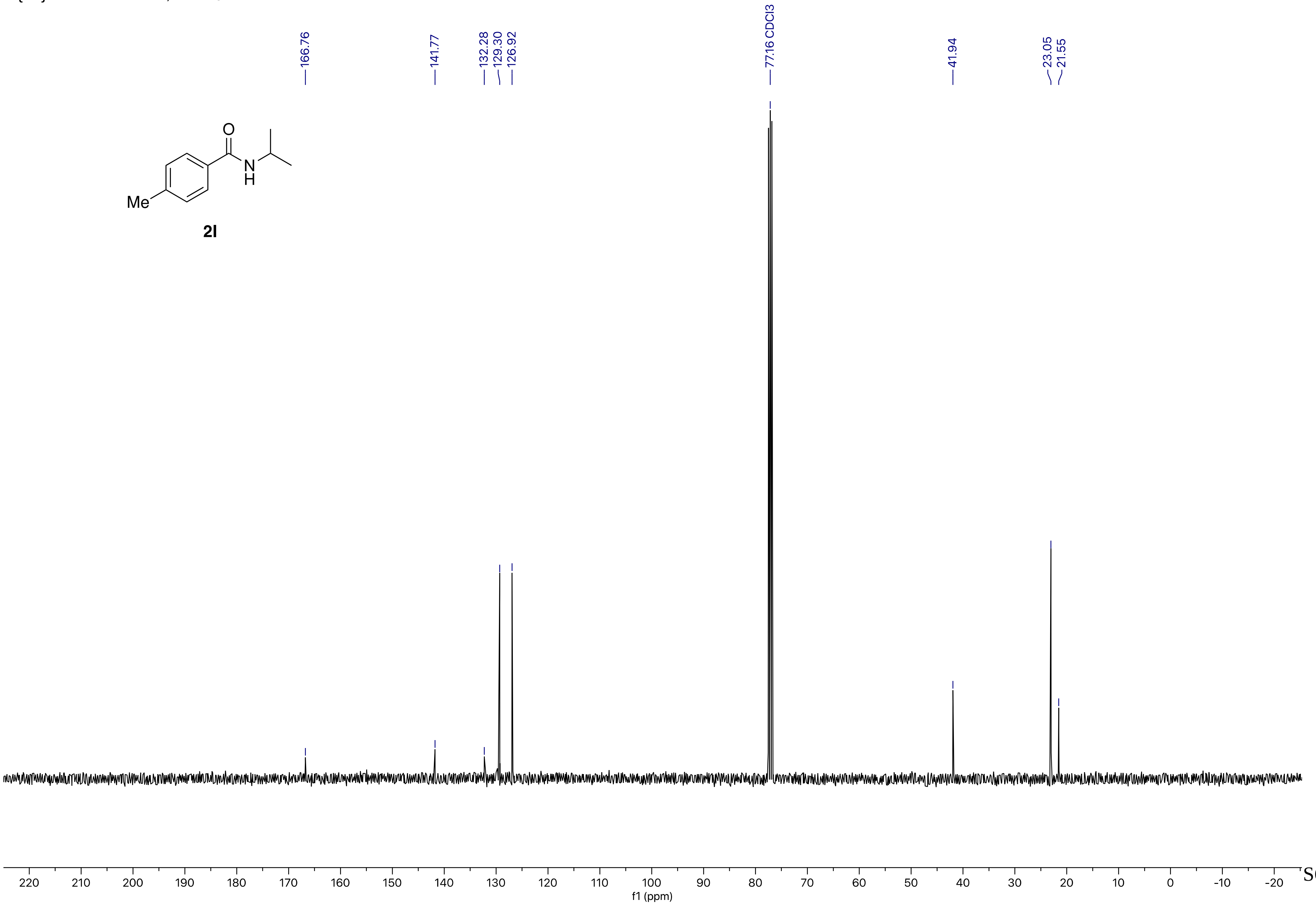

^{1}H NMR: 500 MHz, $CDCl_3$

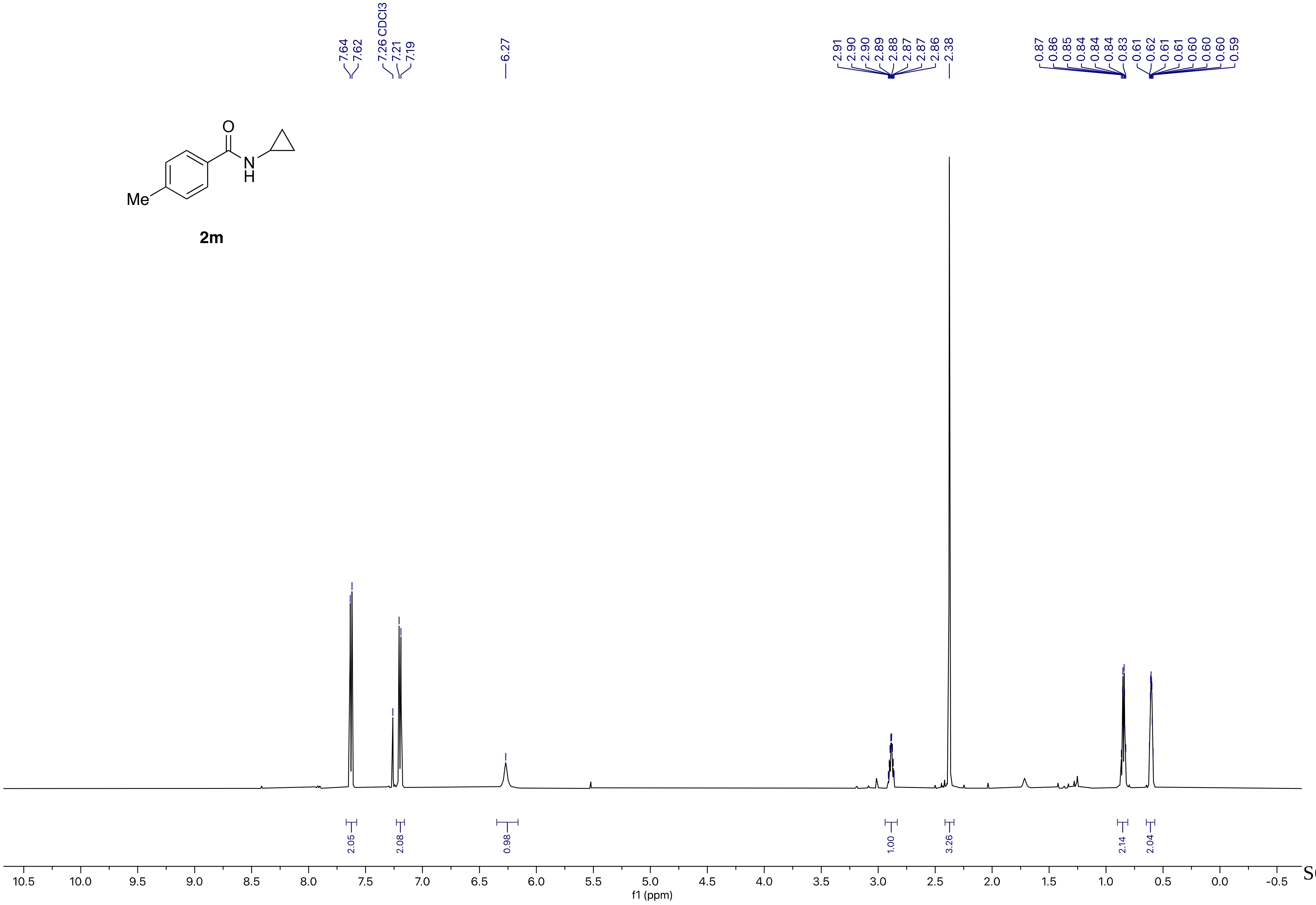

$^{13}C\{^1H\}$ NMR: 126 MHz, $CDCl_3$

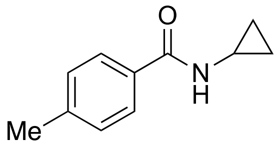

2m

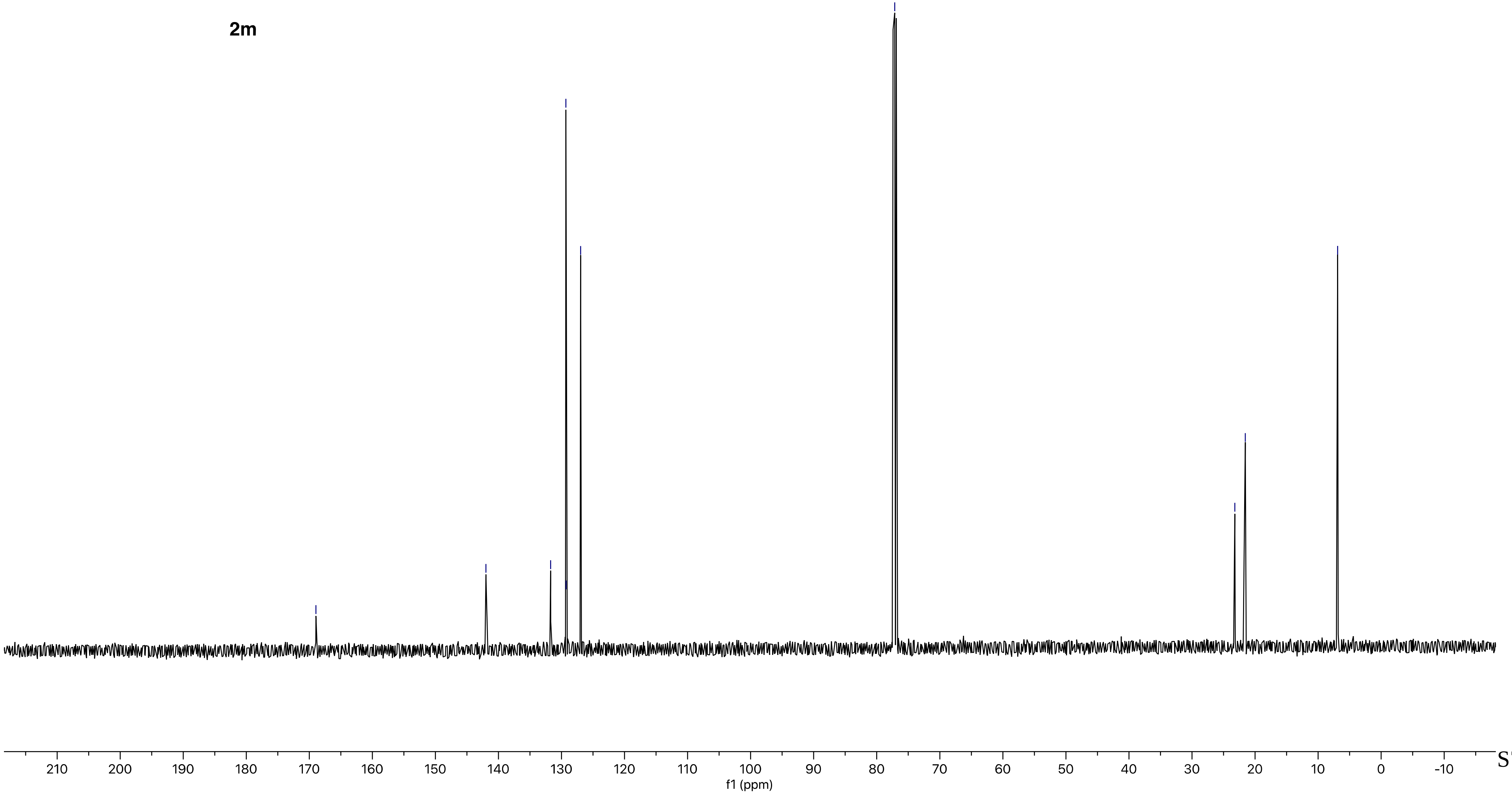

^1H NMR: 500 MHz, $CDCl_3$

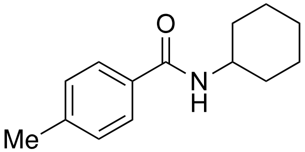

2n

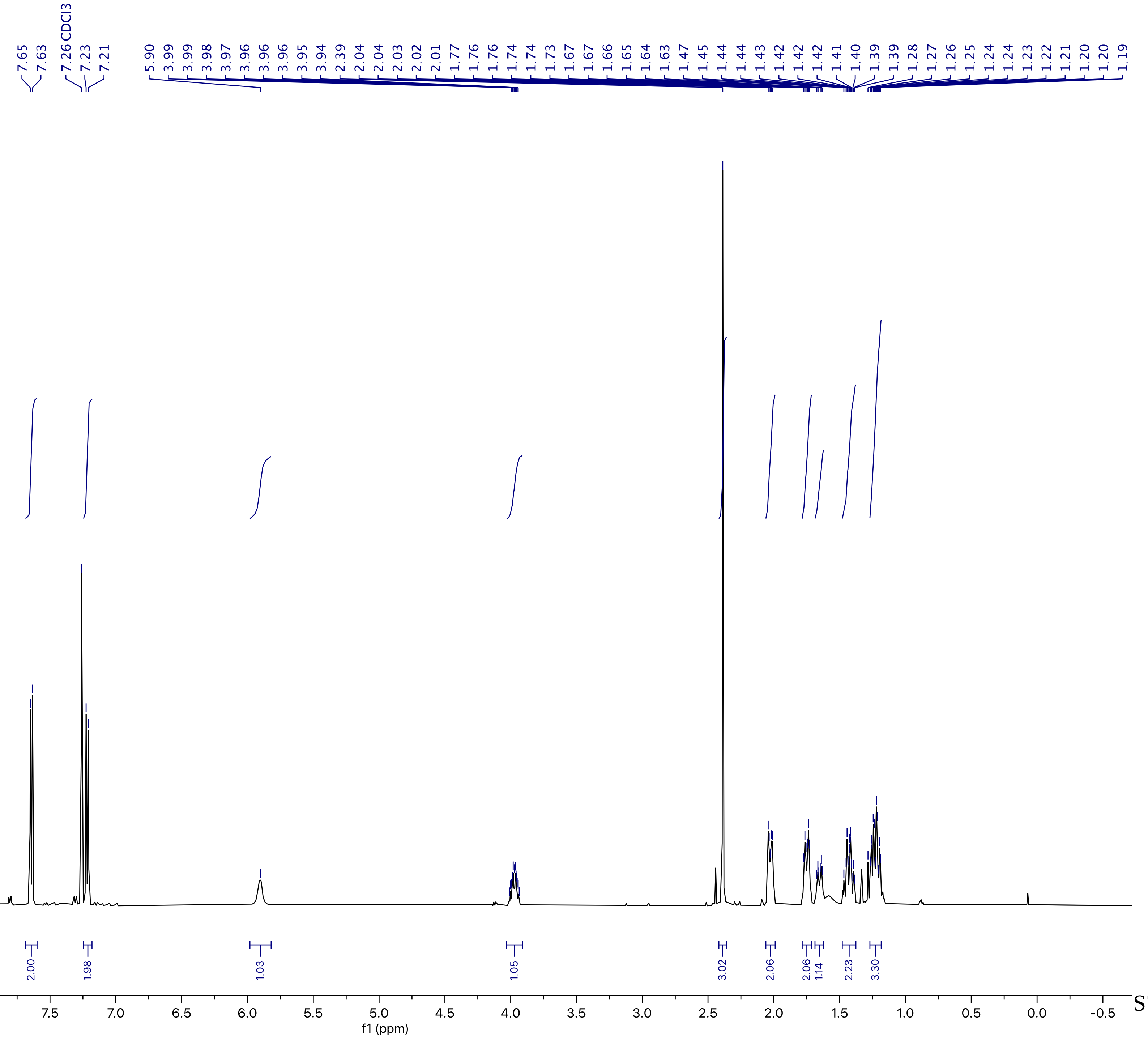

$^{13}C\{^{1}H\}$ NMR: 126 MHz, $CDCl_3$

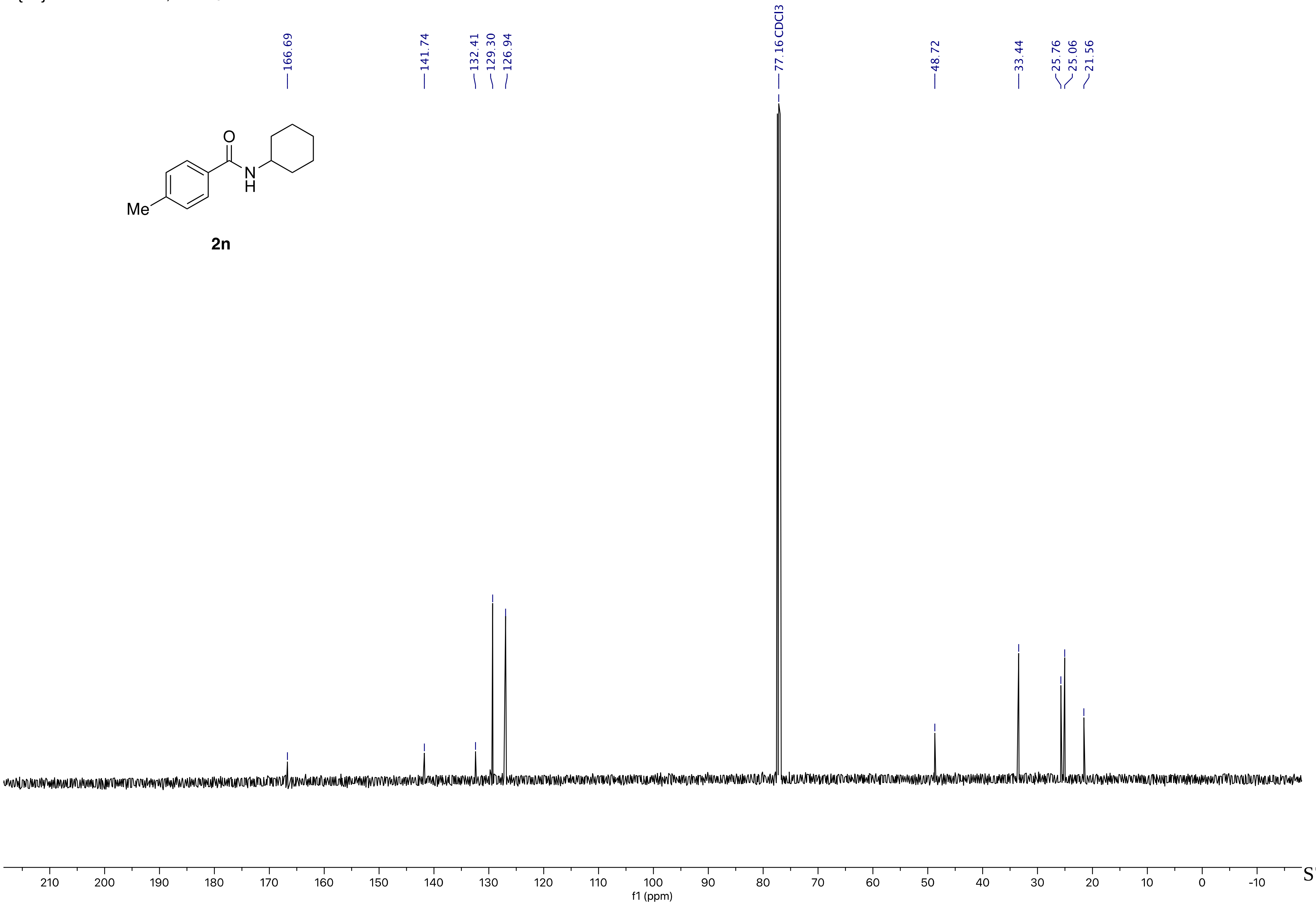

2n

^{1}H NMR: 400 MHz, $CDCl_3$

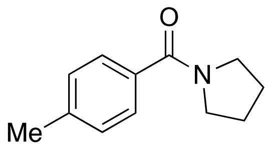

2o

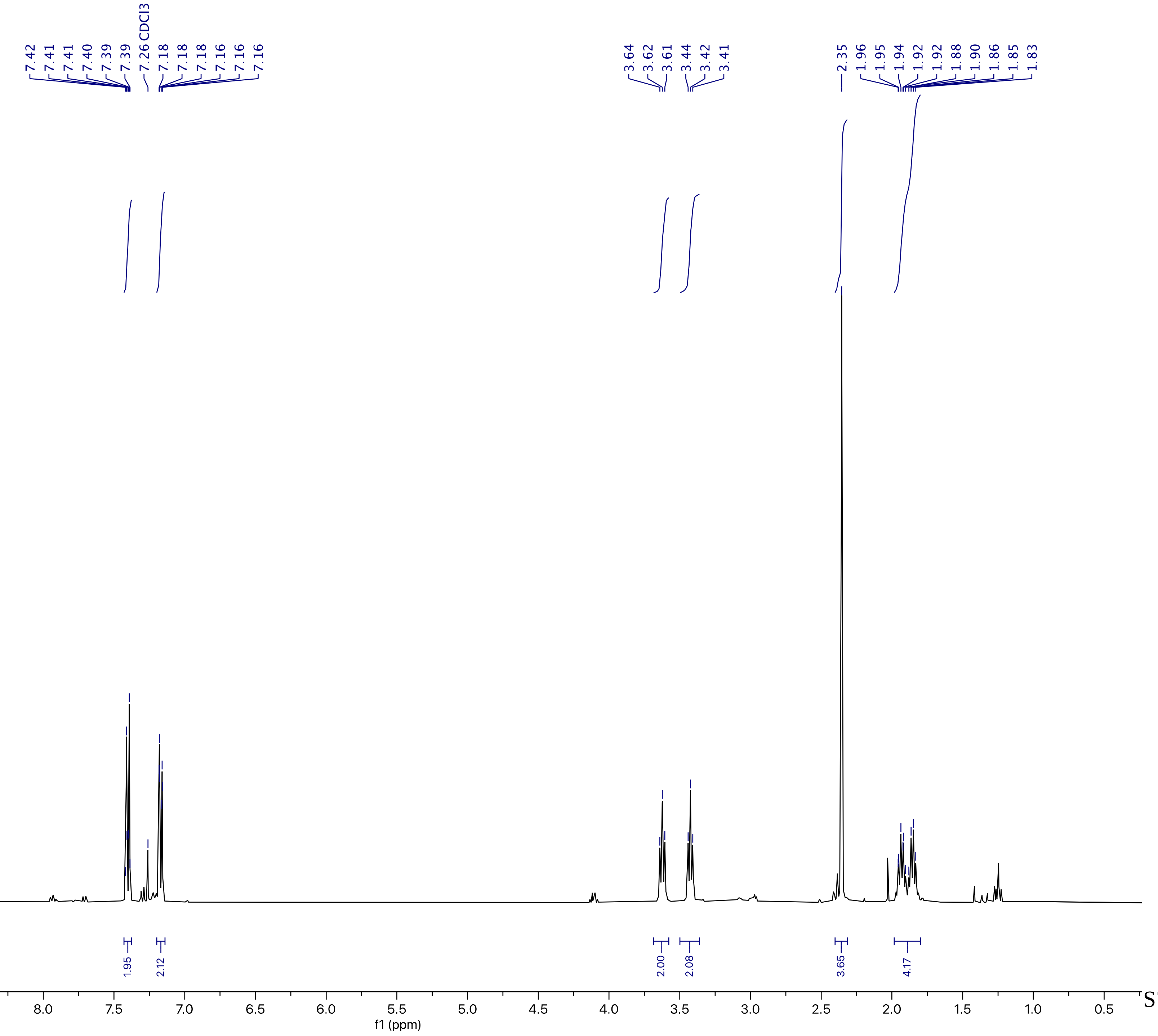

$^{13}C\{^{1}H\}$ NMR: 126 MHz, $CDCl_3$

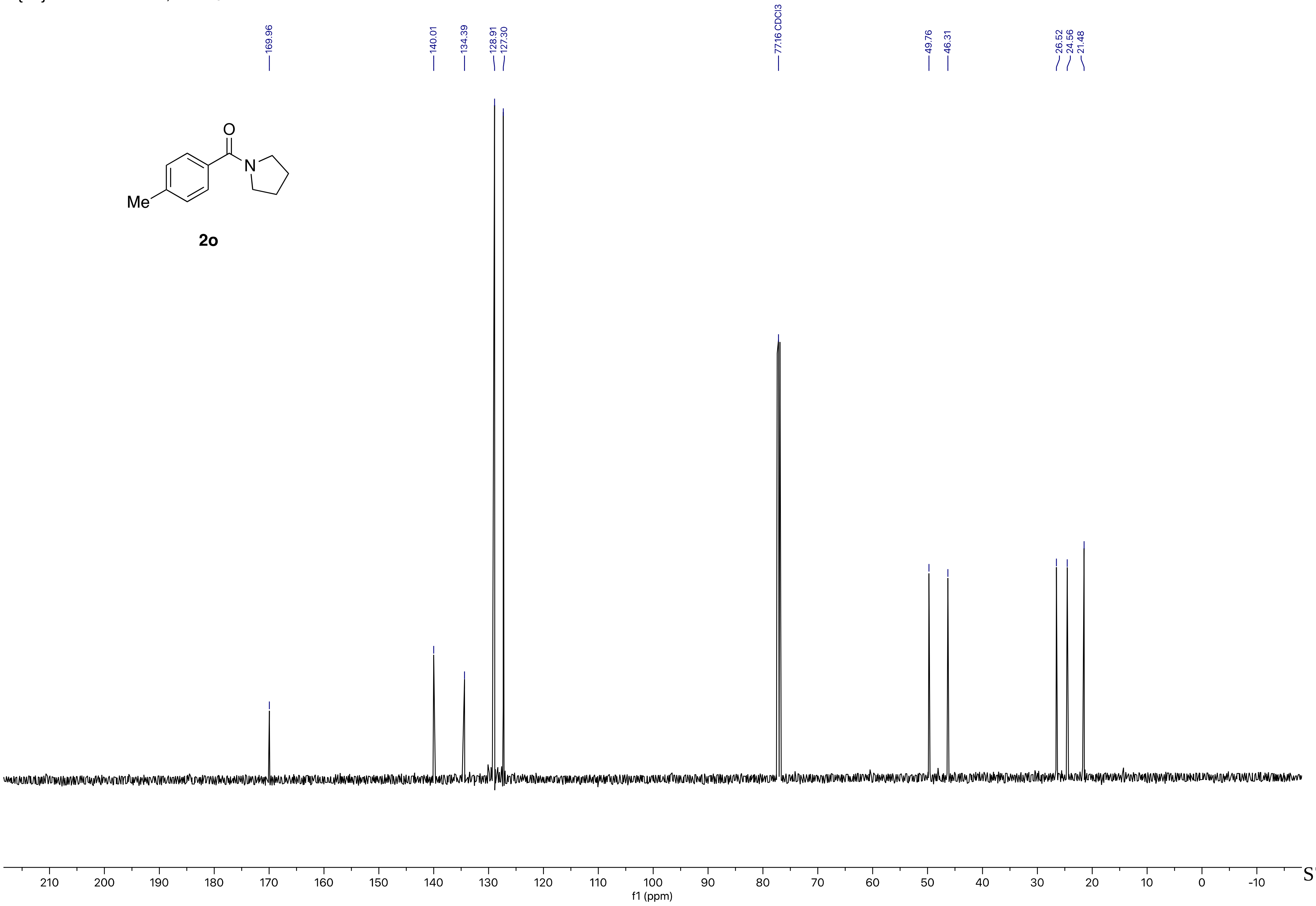

^{1}H NMR: 500 MHz, $CDCl_3$

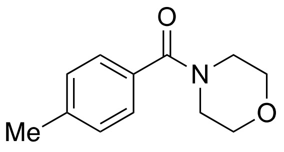

2p

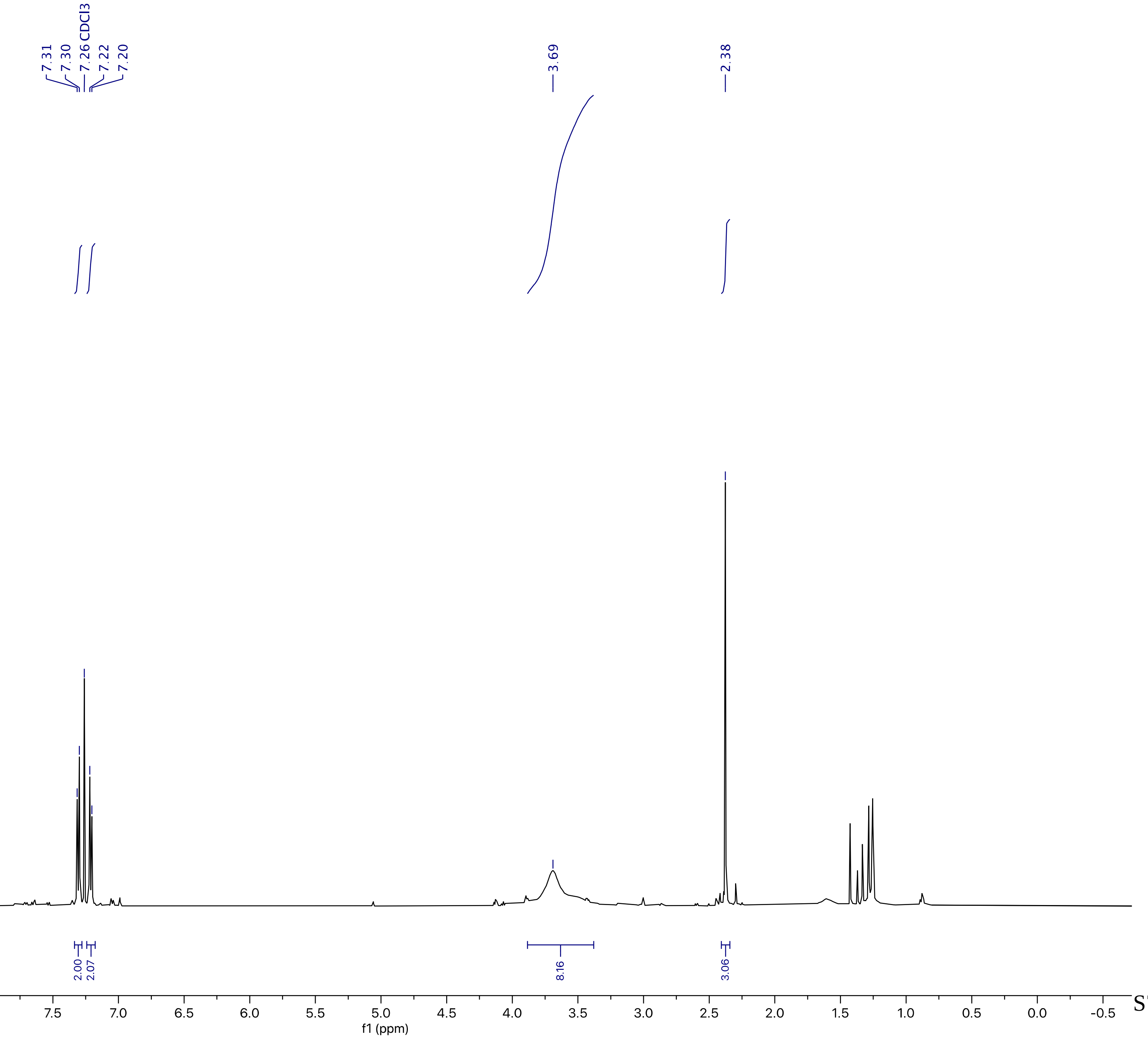

$^{13}C\{^1H\}$ NMR: 126 MHz, $CDCl_3$

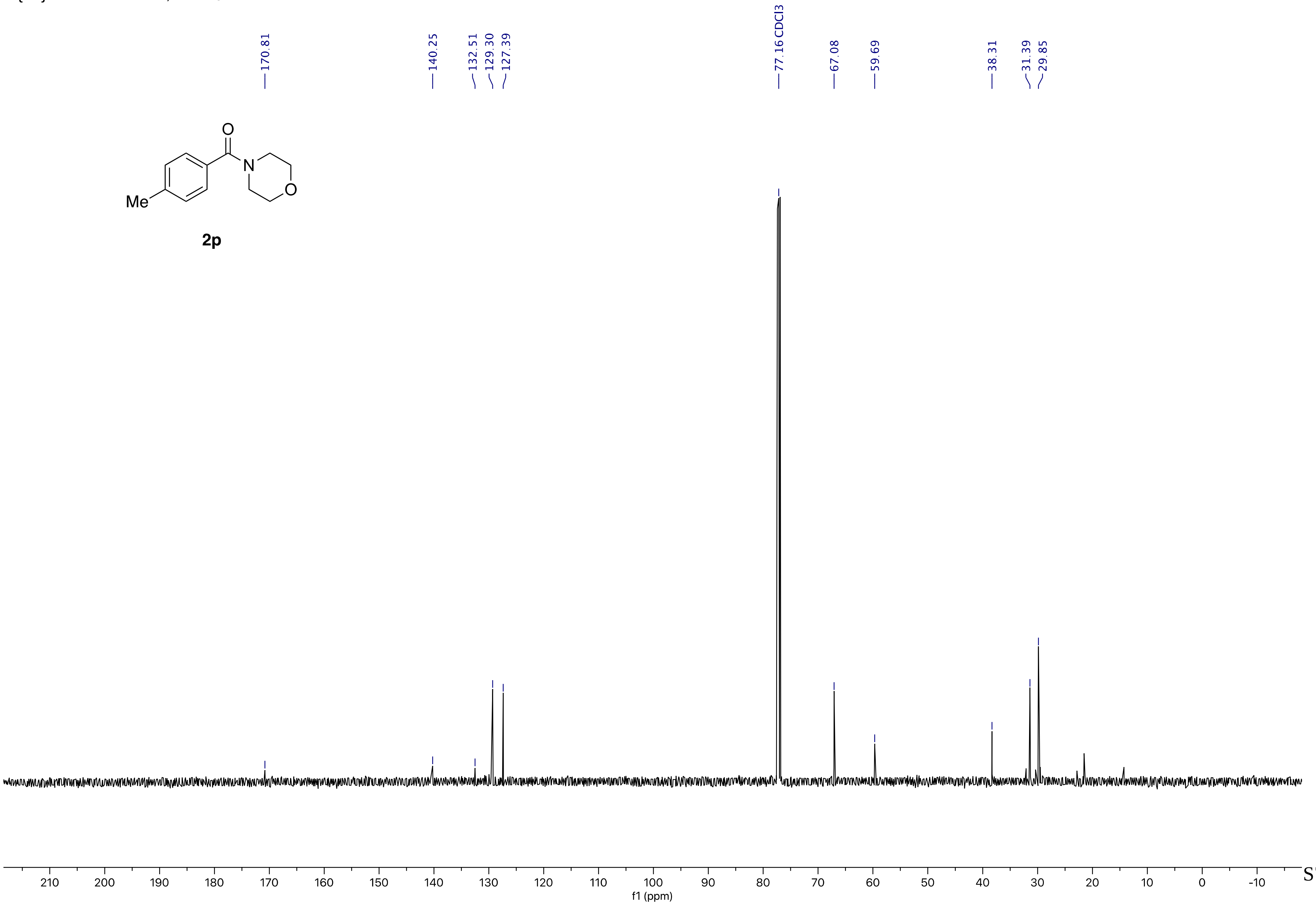

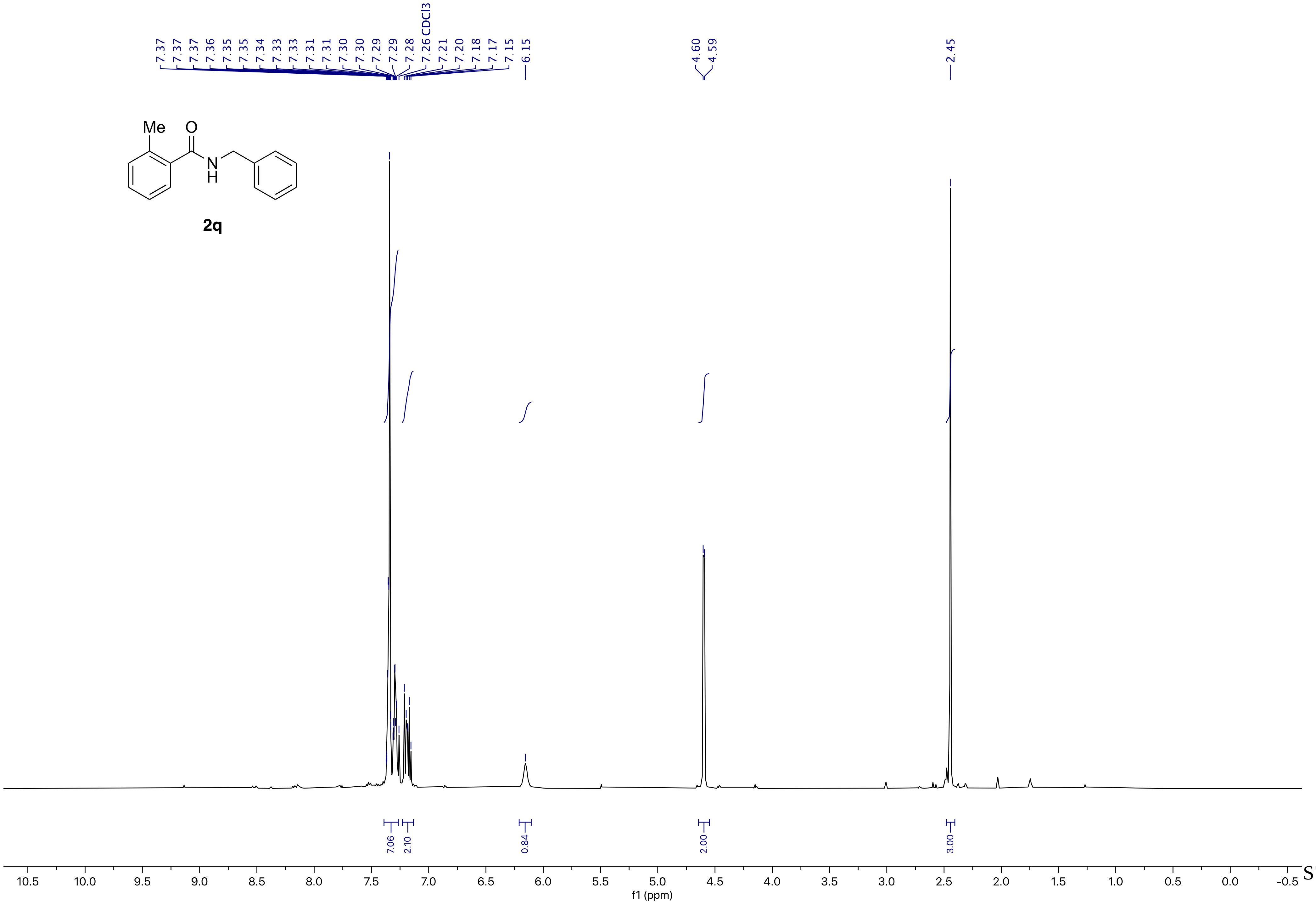
7.37
7.37
7.37
7.36
7.35
7.35
7.34
7.33
7.33
7.31
7.31
7.30
7.30
7.29
7.29
7.28
7.26 CDCl3
7.21
7.20
7.18
7.17
7.15
6.15
4.60
4.59
2.45
Me
O
N
H
2q
7.06
2.10
0.84
2.00
3.00
10.5
10.0
9.5
9.0
8.5
8.0
7.5
7.0
6.5
6.0
5.5
5.0
4.5
4.0
3.5
3.0
2.5
2.0
1.5
1.0
0.5
0.0
-0.5
f1 (ppm)

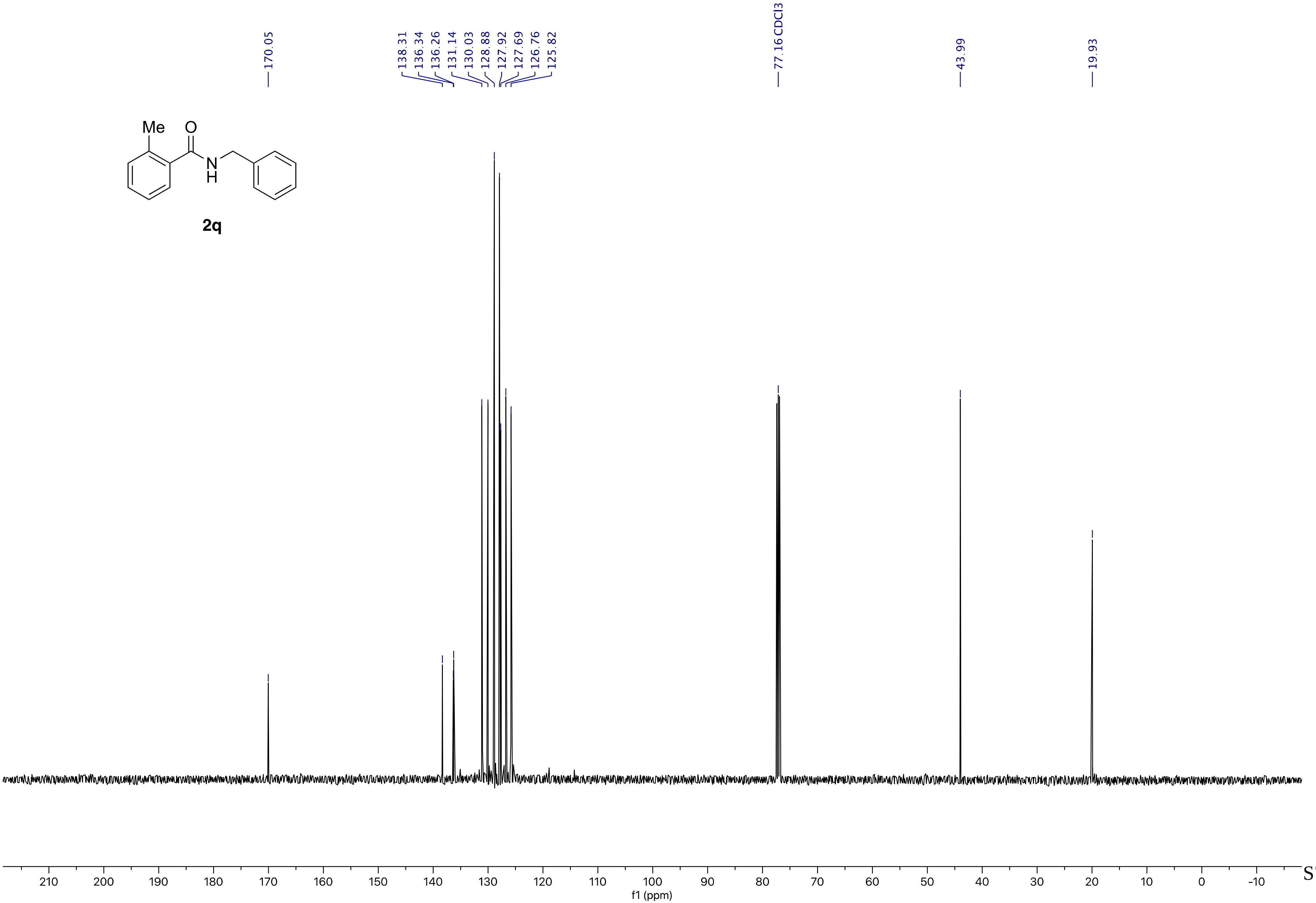
170.05
138.31
136.34
136.26
131.14
130.03
128.88
127.92
127.69
126.76
125.82
77.16 CDCl3
43.99
19.93
210
200
190
180
170
160
150
140
130
120
110
100
90
80
70
60
50
40
30
20
10
0
-10
f1 (ppm)

2q

^1H NMR: 400 MHz, $CDCl_3$

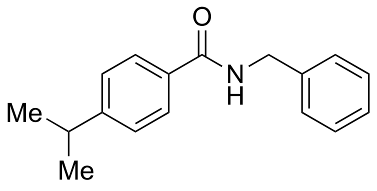

2r

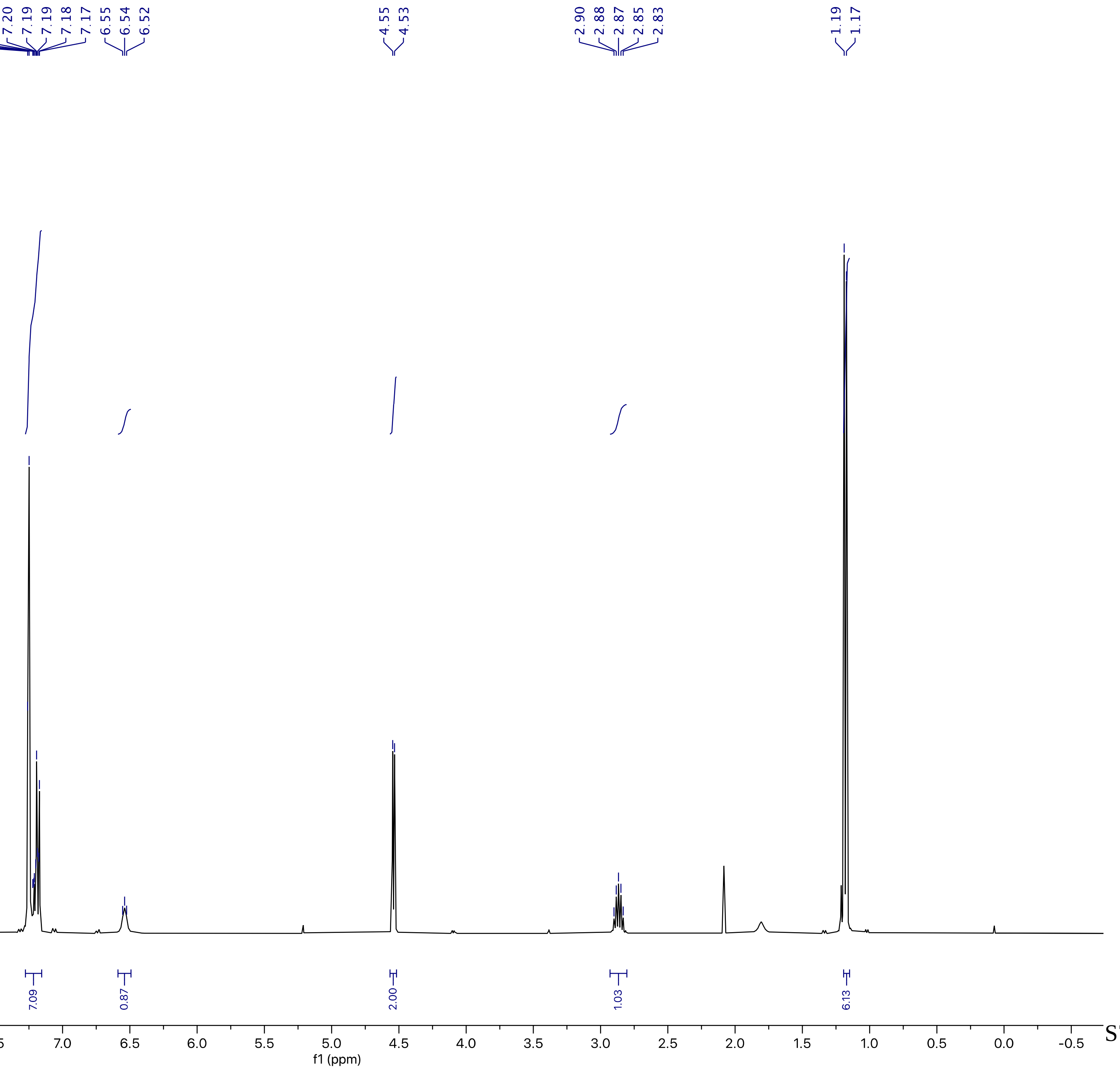

$^{13}C\{^1H\}$ NMR: 101 MHz, $CDCl_3$

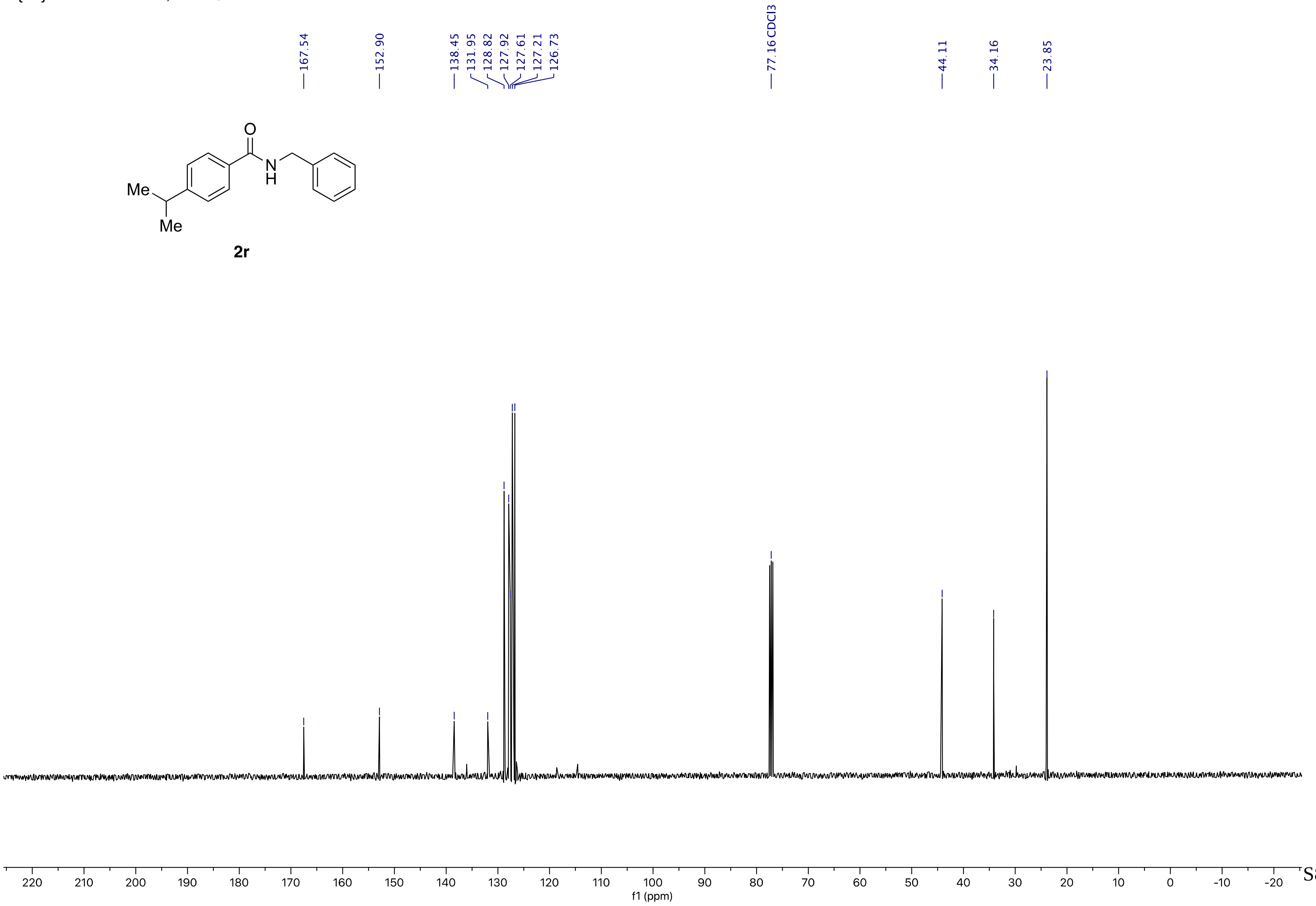

^{1}H NMR: 400 MHz, $CDCl_3$

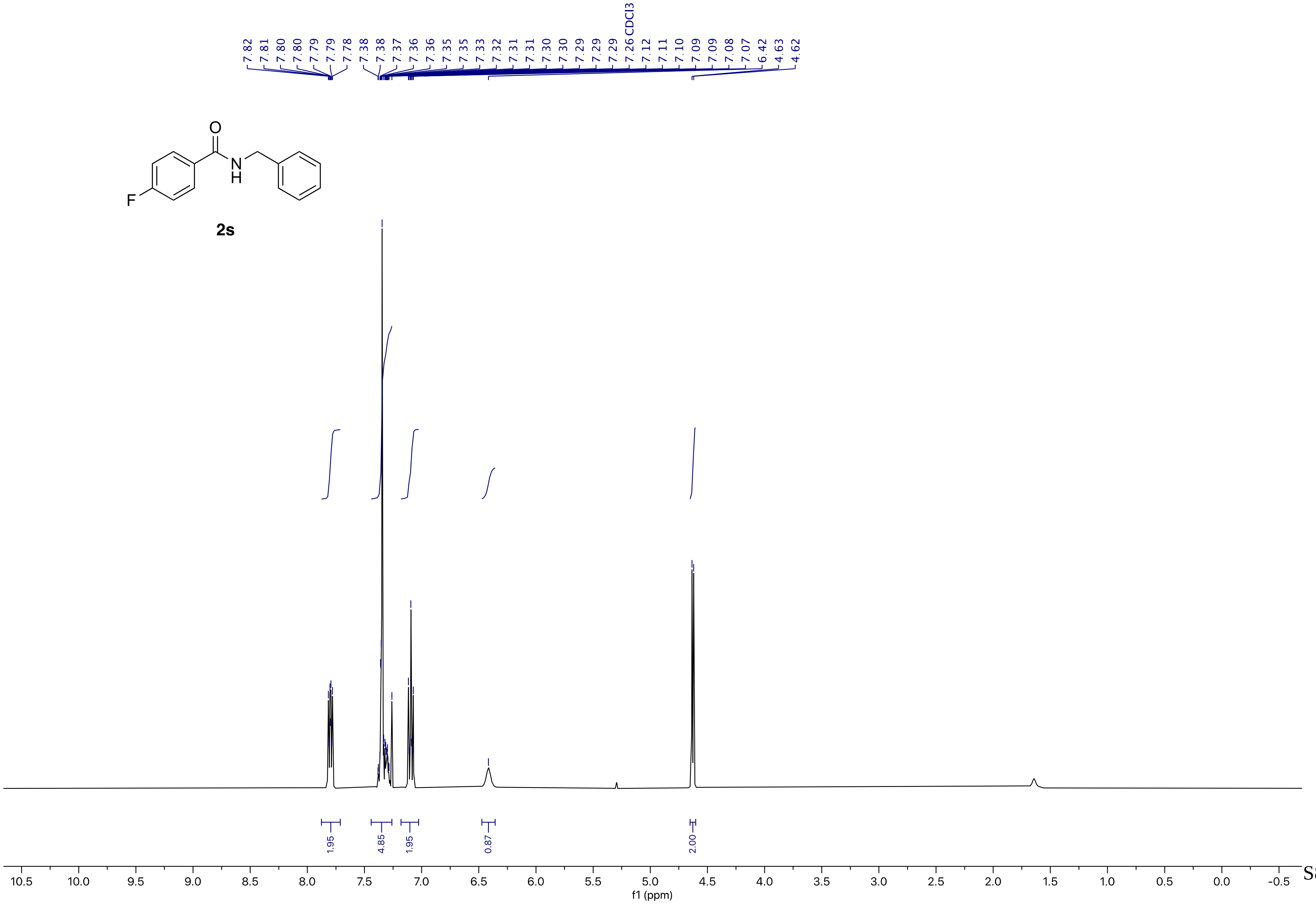

$^{13}C\{^1H\}$ NMR: 101 MHz, $CDCl_3$

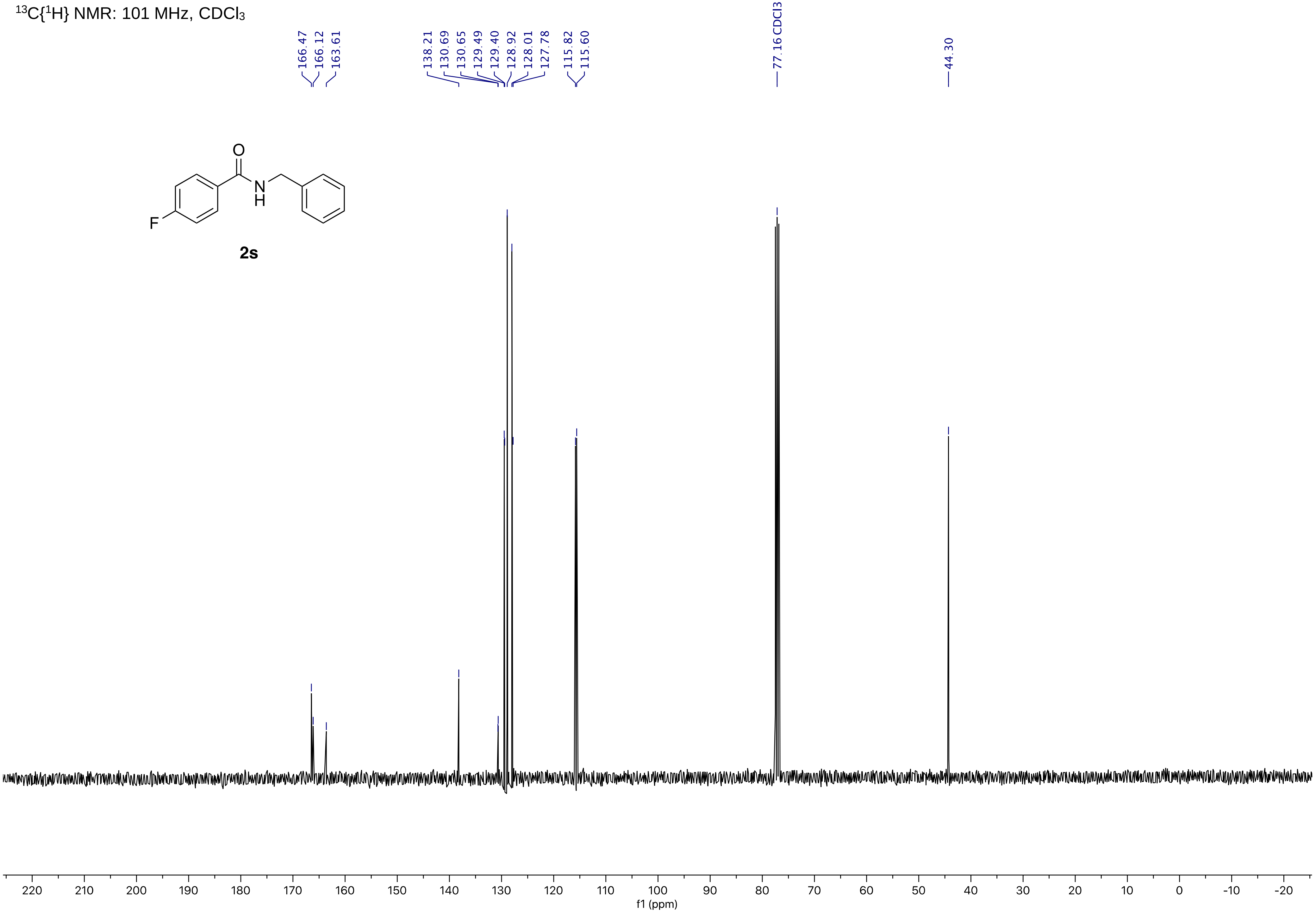

2s

^{19}F NMR: 376 MHz, $CDCl_3$

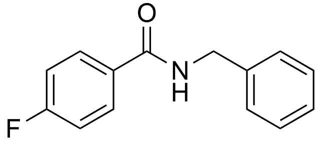

2s

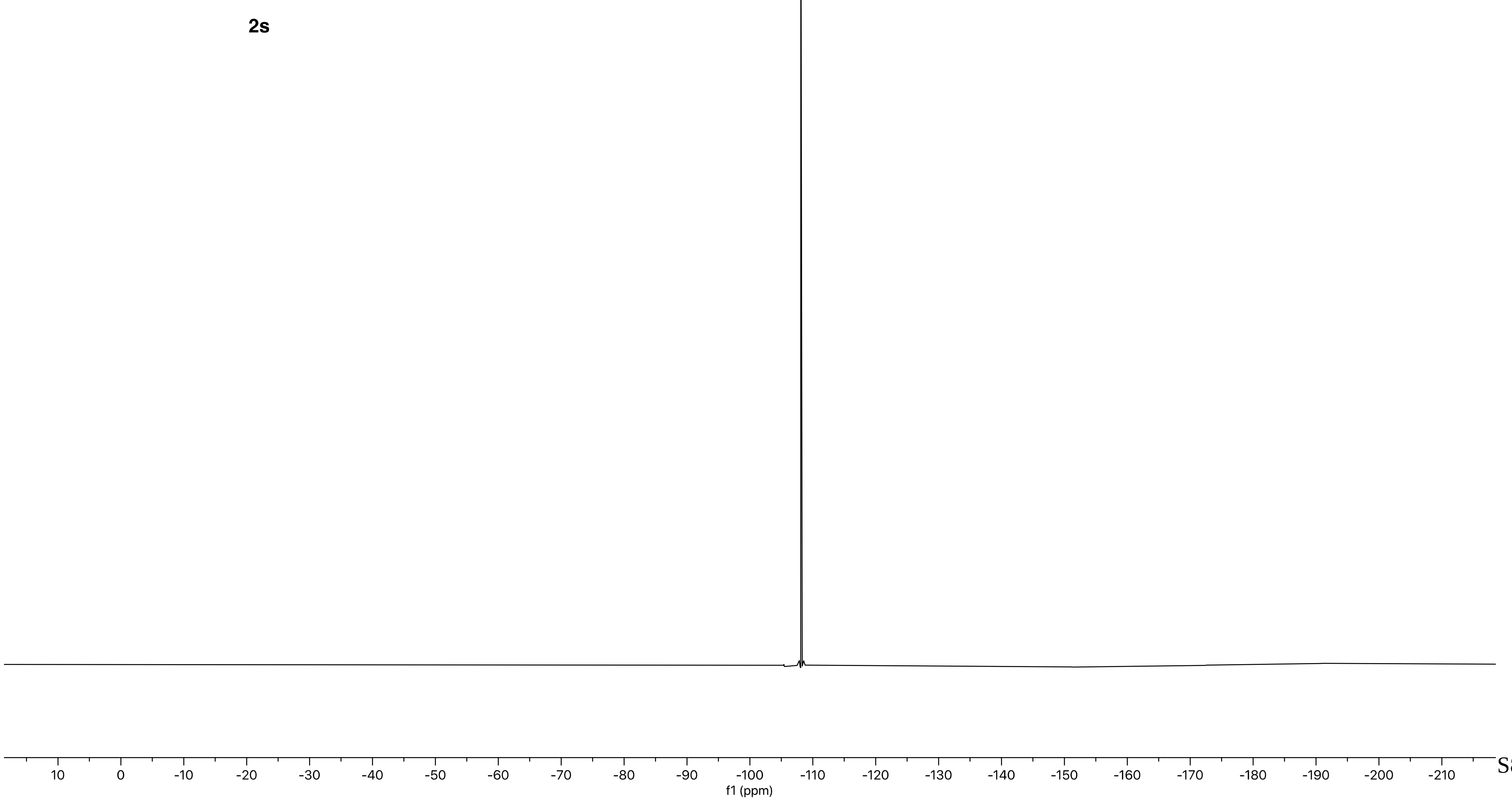

^1H NMR: 500 MHz, $CDCl_3$

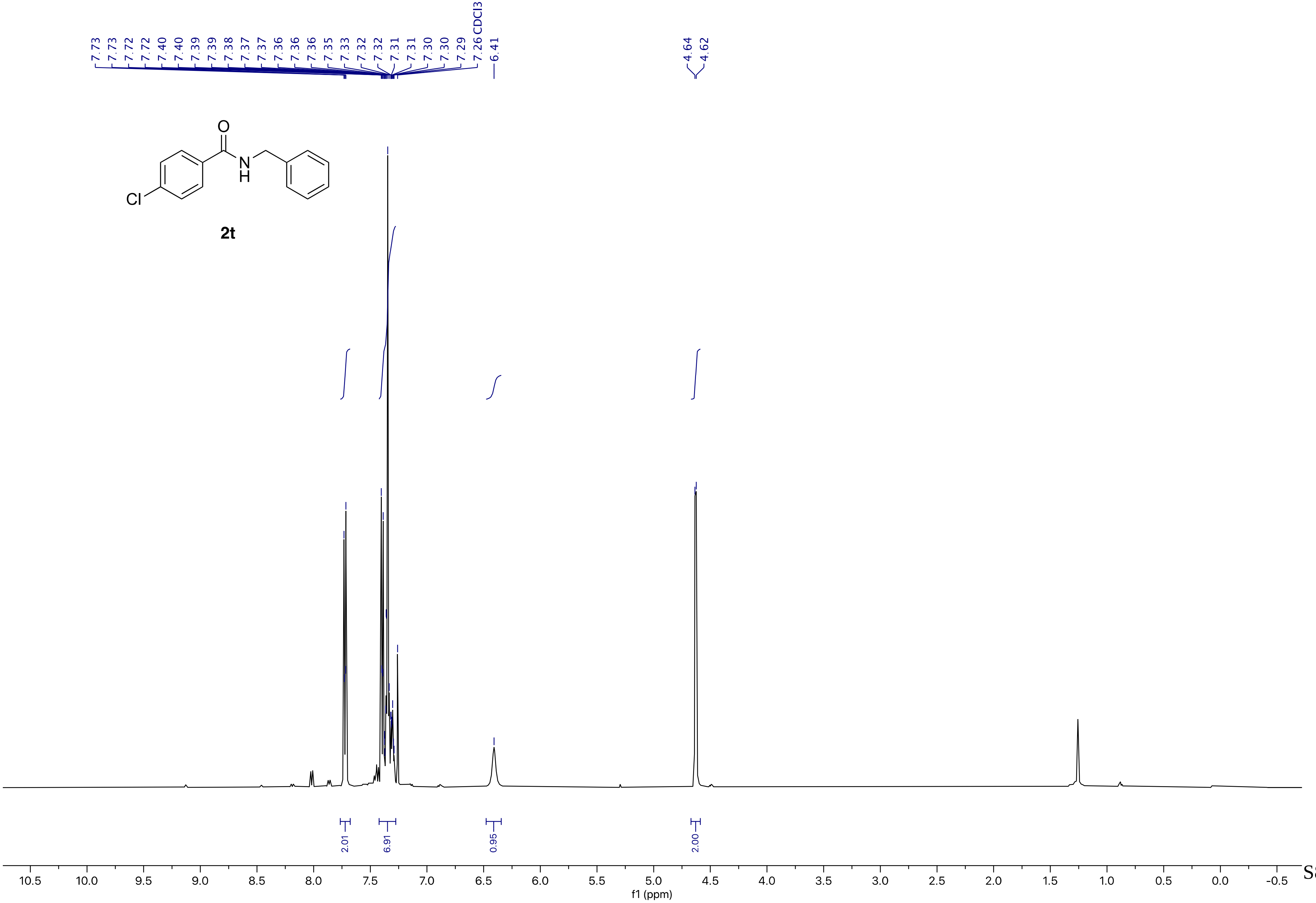

2t

$^{13}C\{^{1}H\}$ NMR: 126 MHz, $CDCl_3$

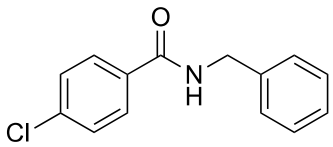

2t

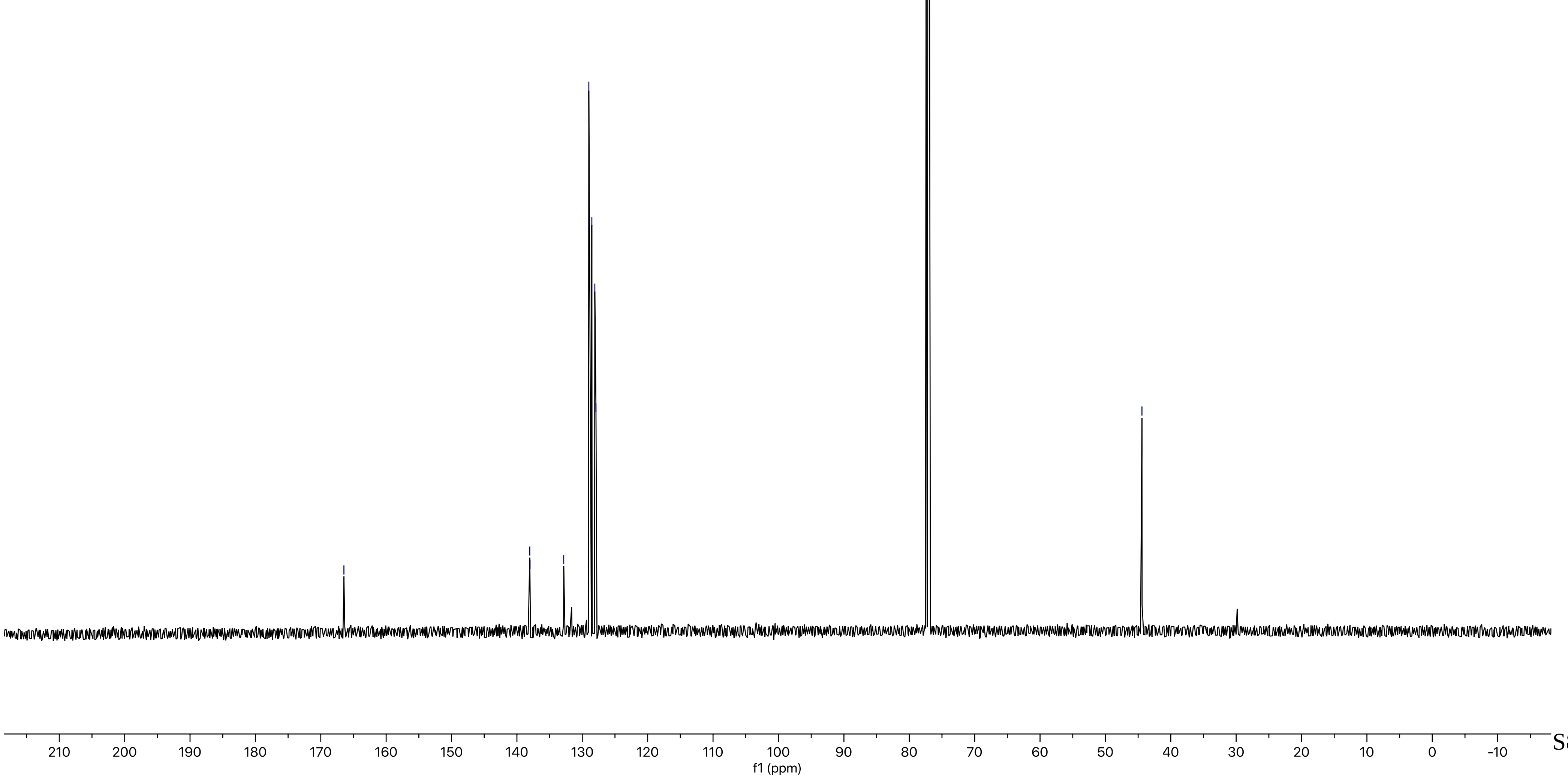

^{1}H NMR: 400 MHz, $CDCl_3$

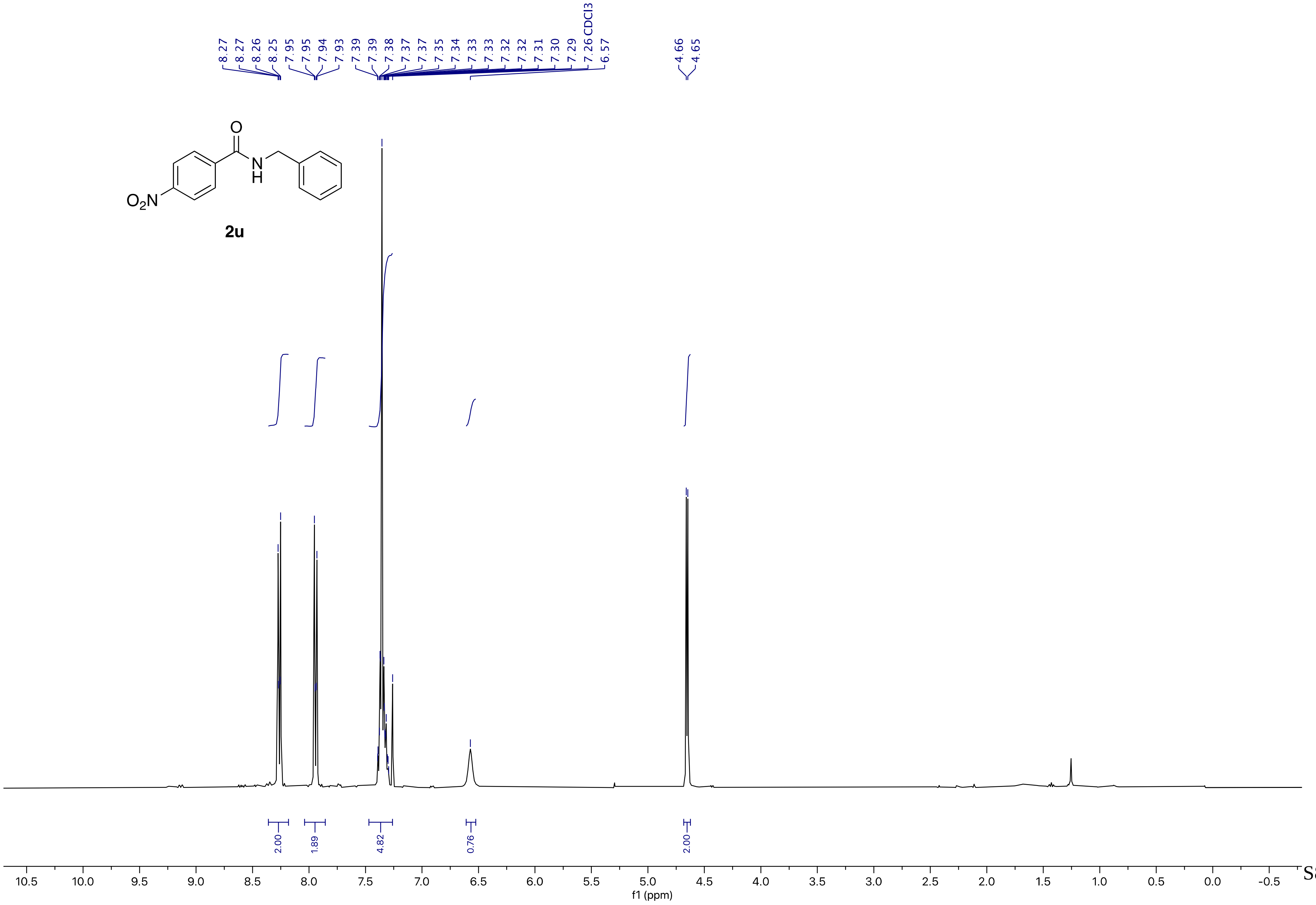

2u

$^{13}C\{^1H\}$ NMR: 101 MHz, $CDCl_3$

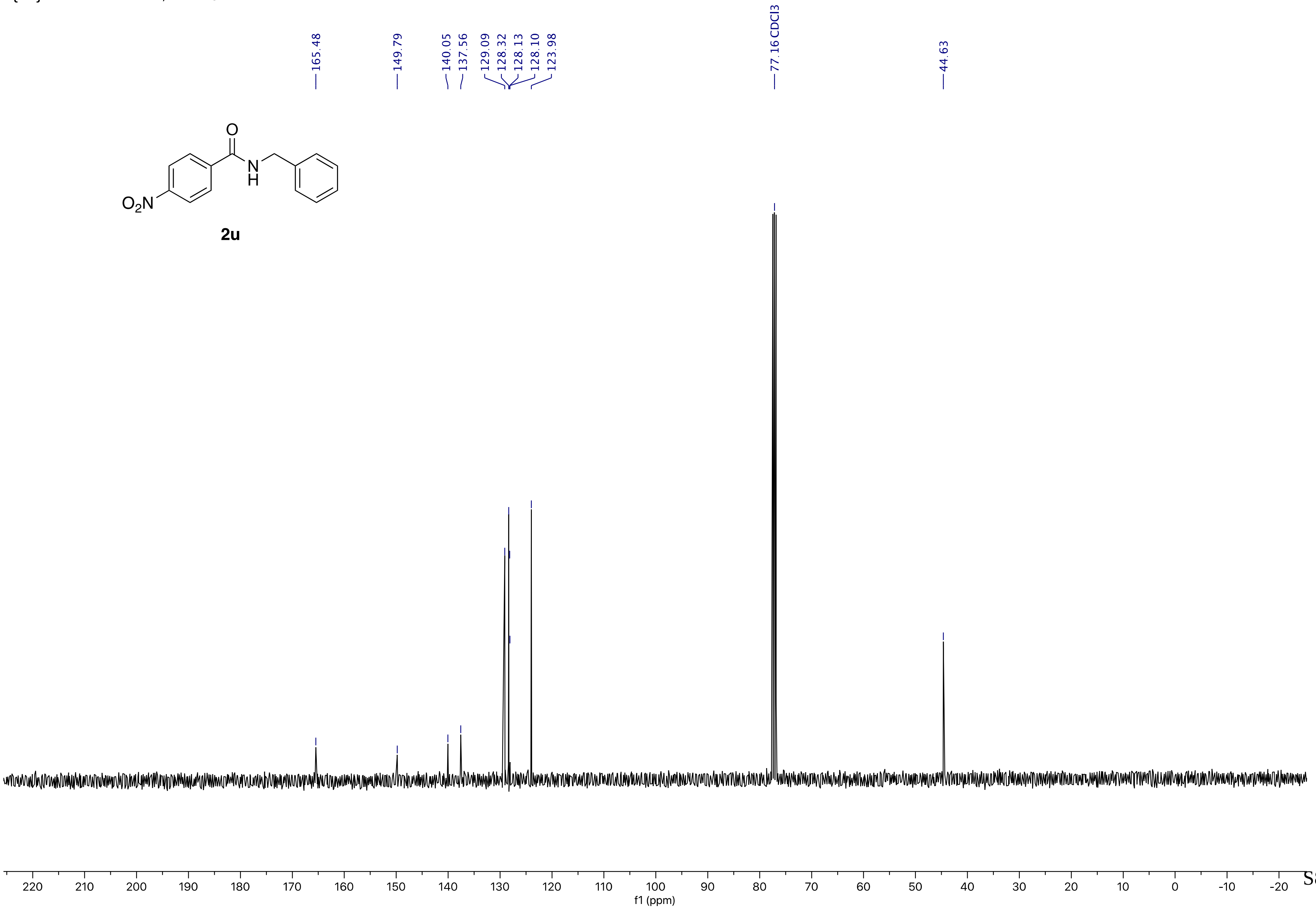

^{1}H NMR: 500 MHz, CDCl$_3$

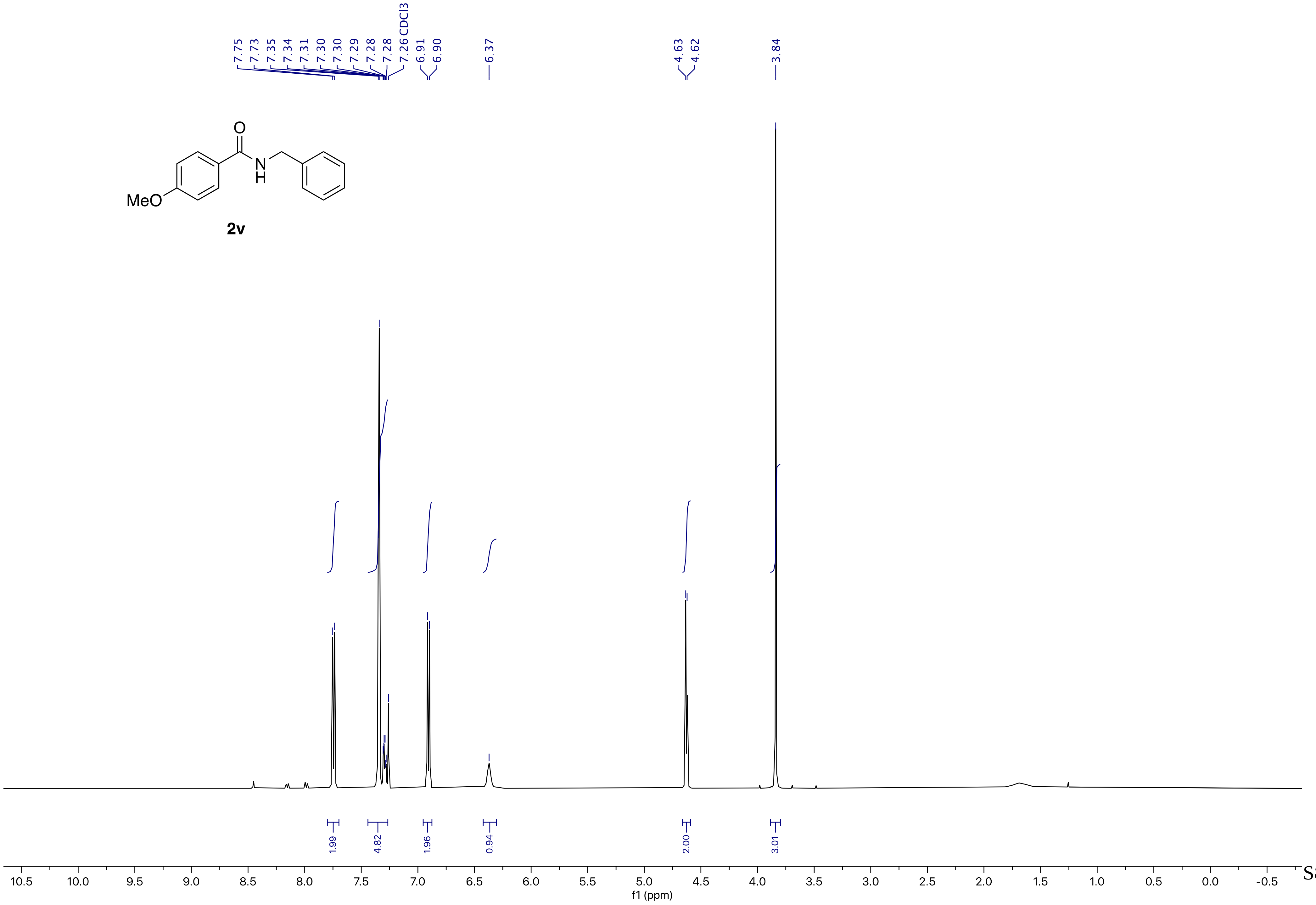

2v

$^{13}C\{^1H\}$ NMR: 126 MHz, $CDCl_3$

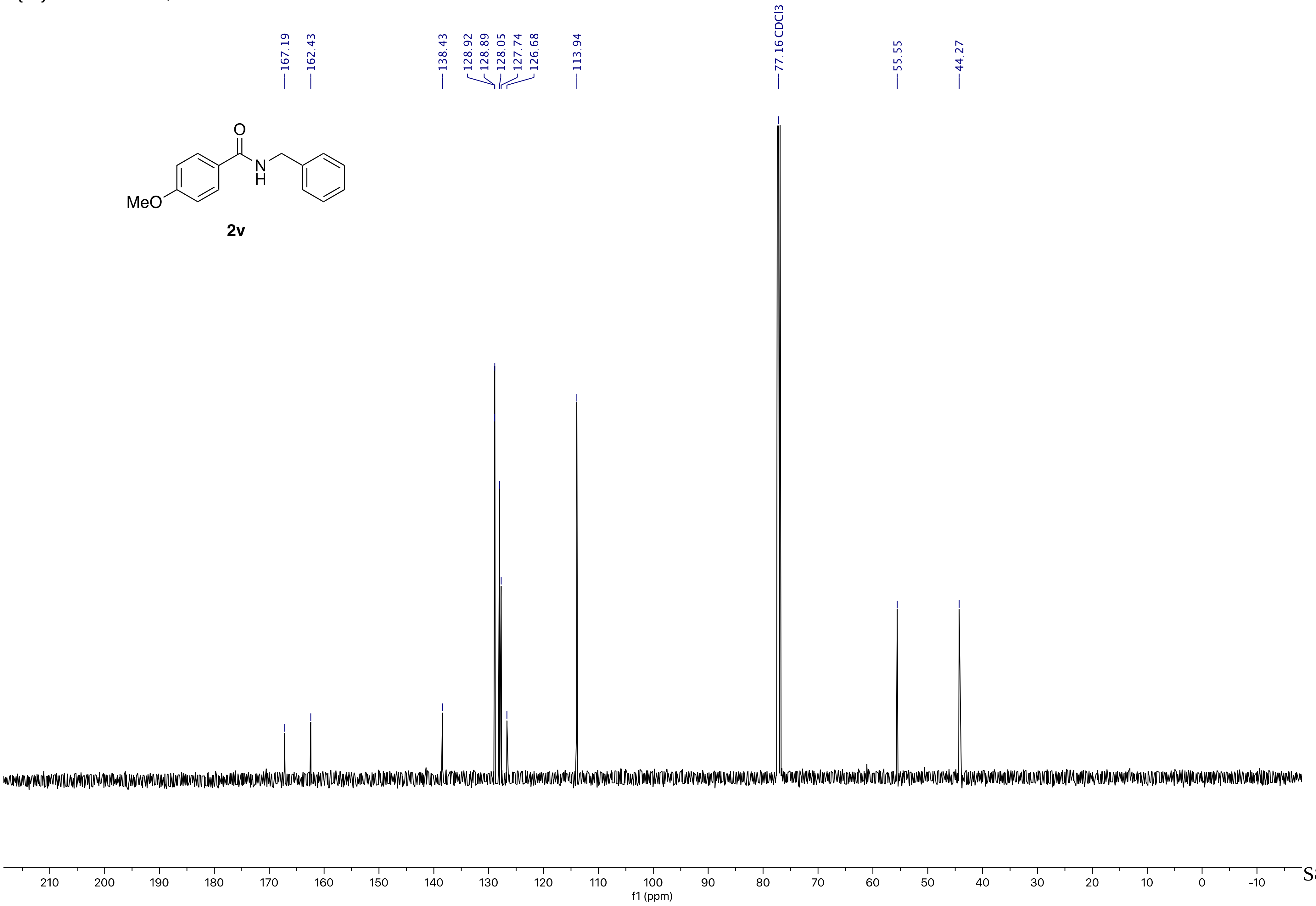

2v

¹H NMR: 400 MHz, $CDCl_3$

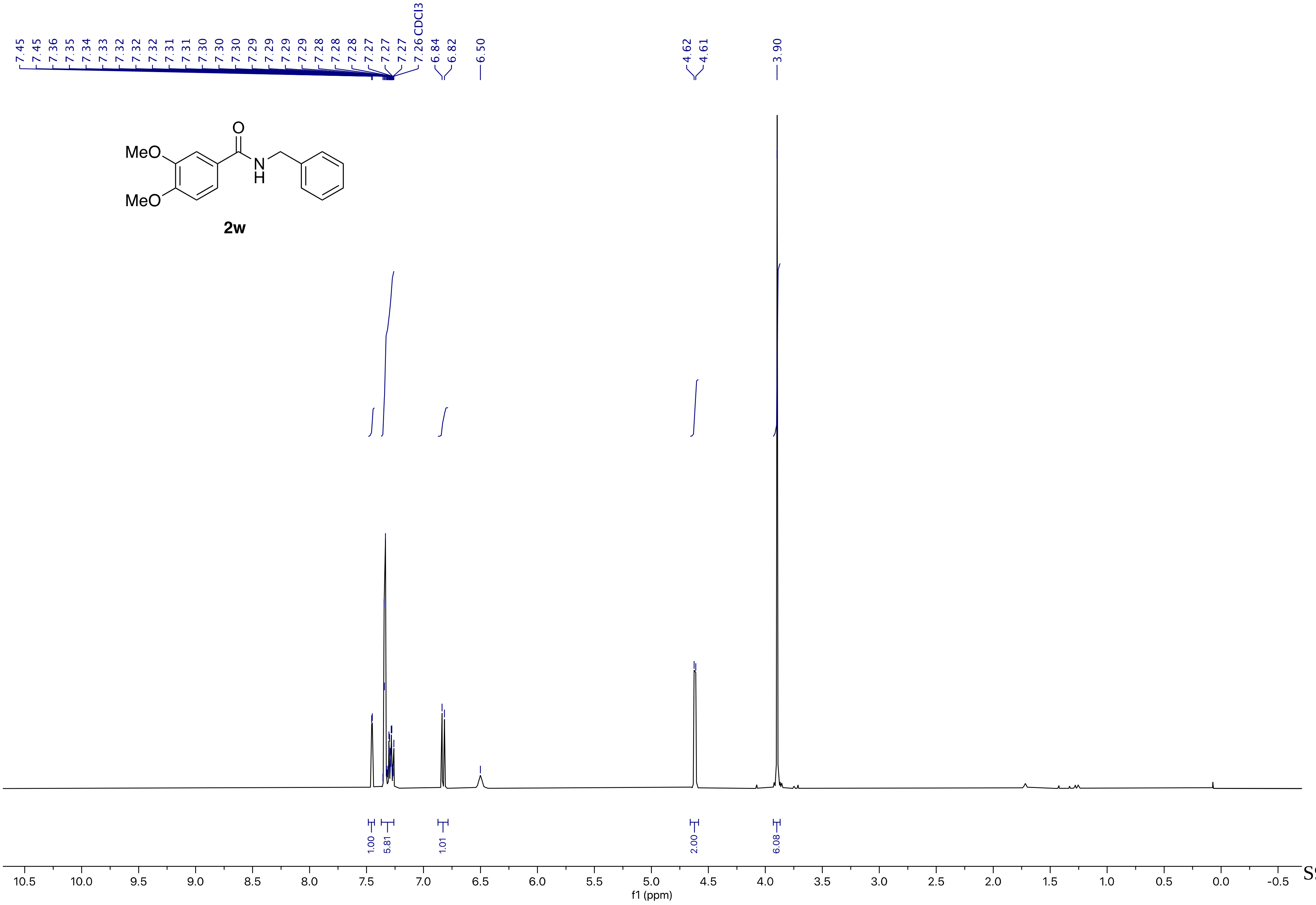

$^{13}C\{^{1}H\}$ NMR: 101 MHz, $CDCl_3$

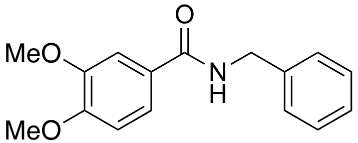

2w

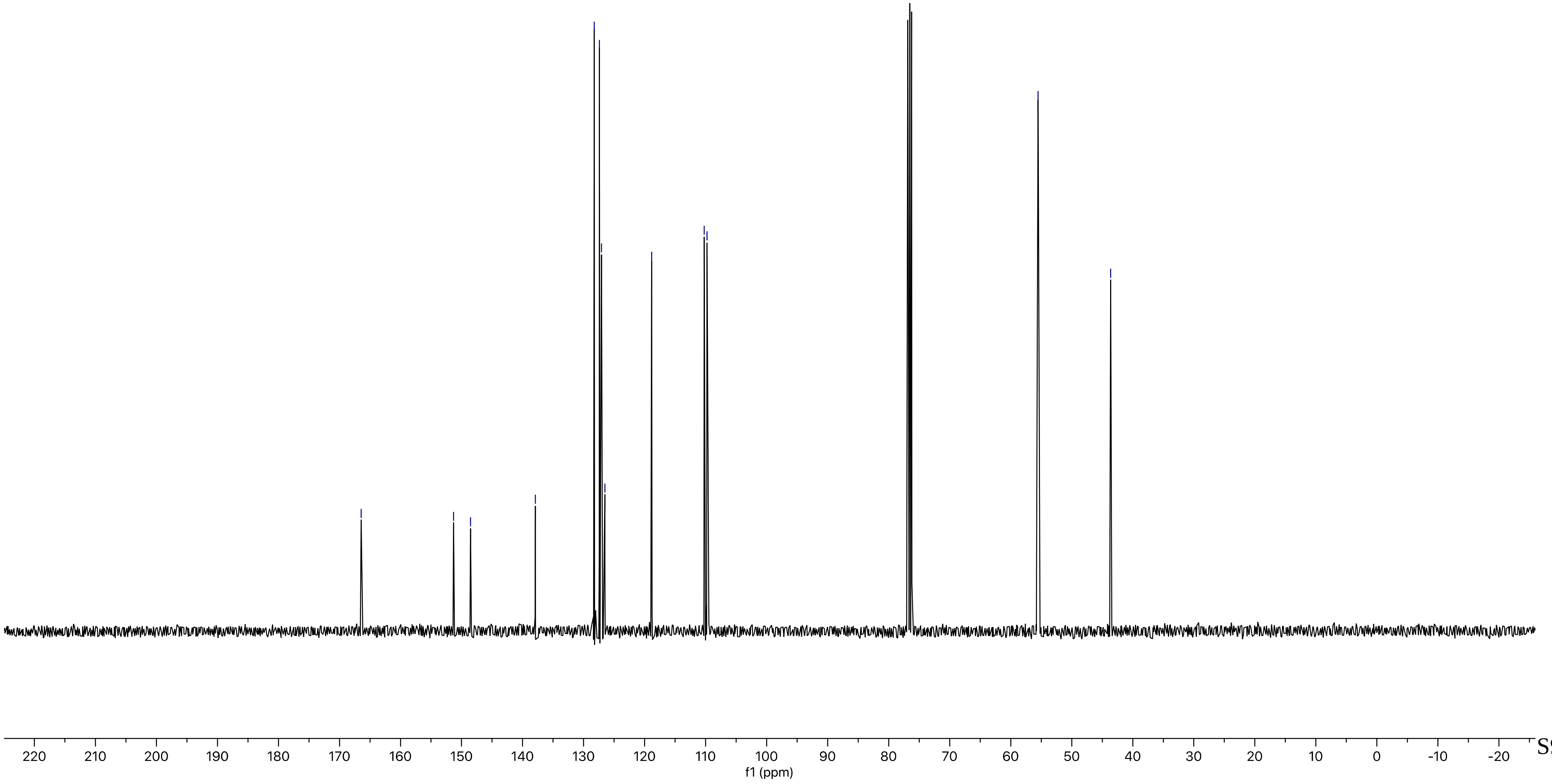

^{1}H NMR: 500 MHz, $CDCl_3$

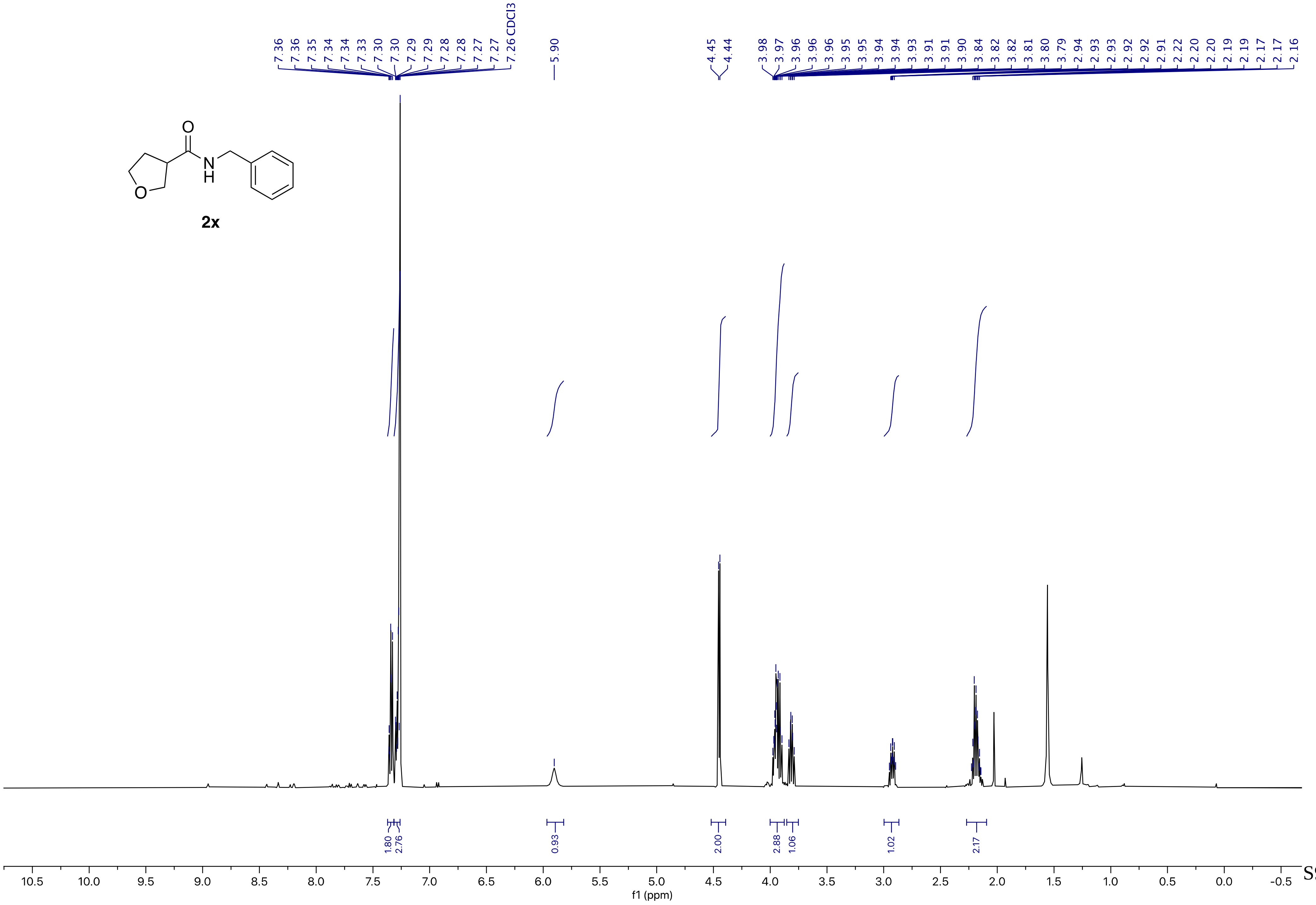

$^{13}C\{^1H\}$ NMR: 126 MHz, $CDCl_3$

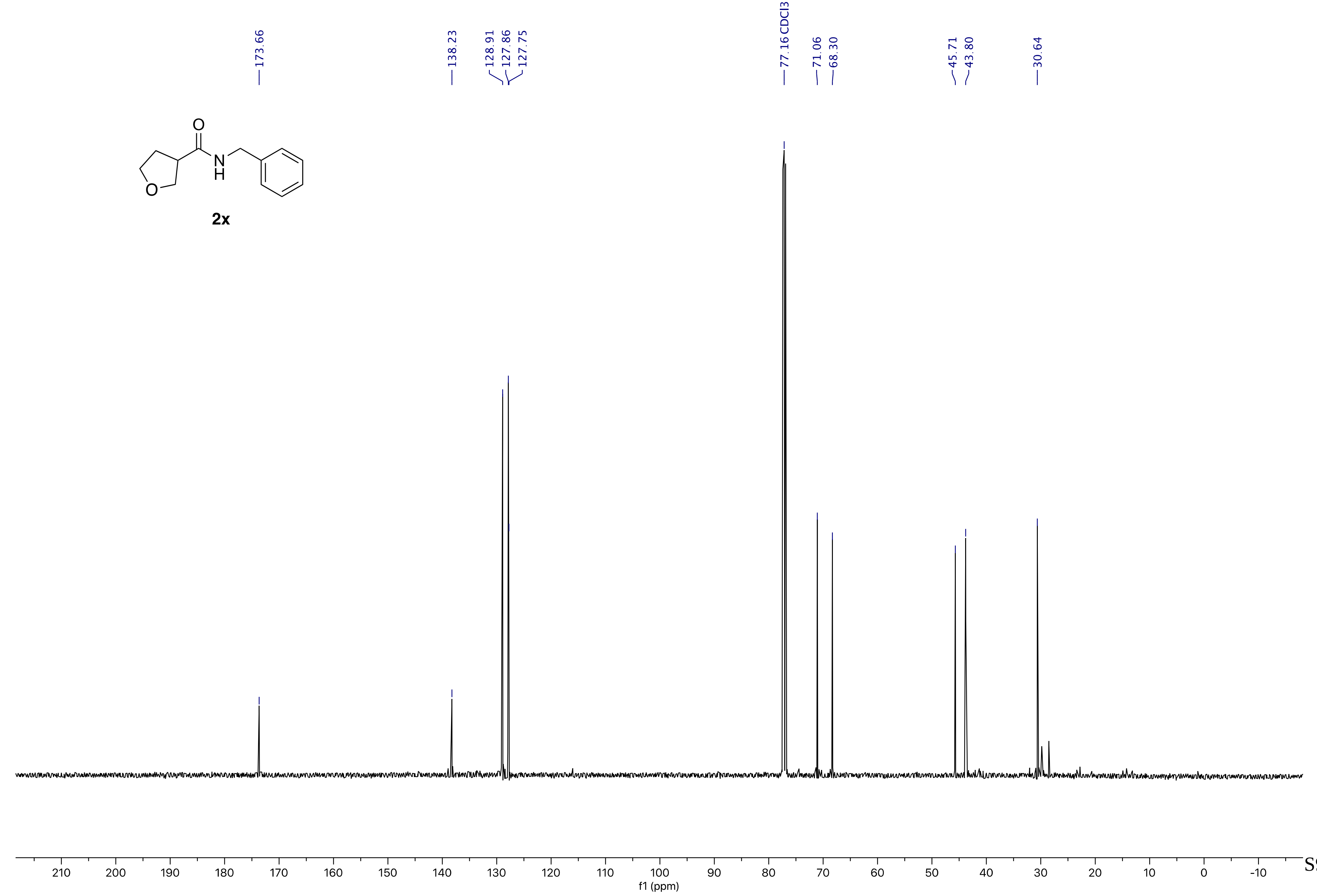

^{1}H NMR: 400 MHz, $CDCl_3$

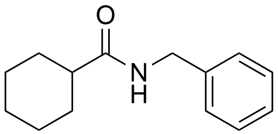

2y

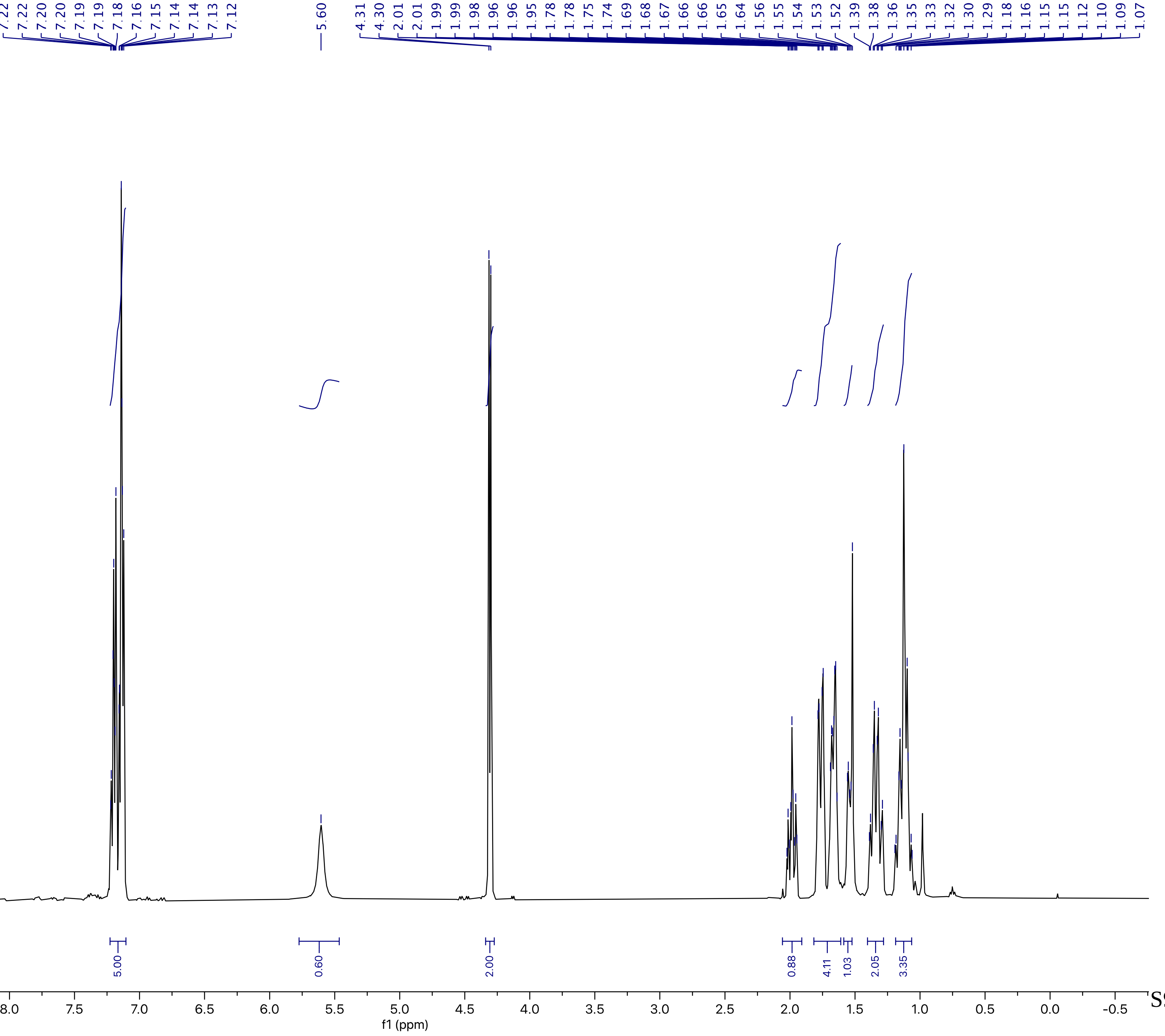

$^{13}C\{^{1}H\}$ NMR: 101 MHz, $CDCl_3$

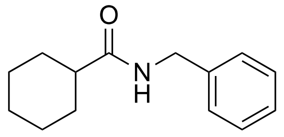

2y

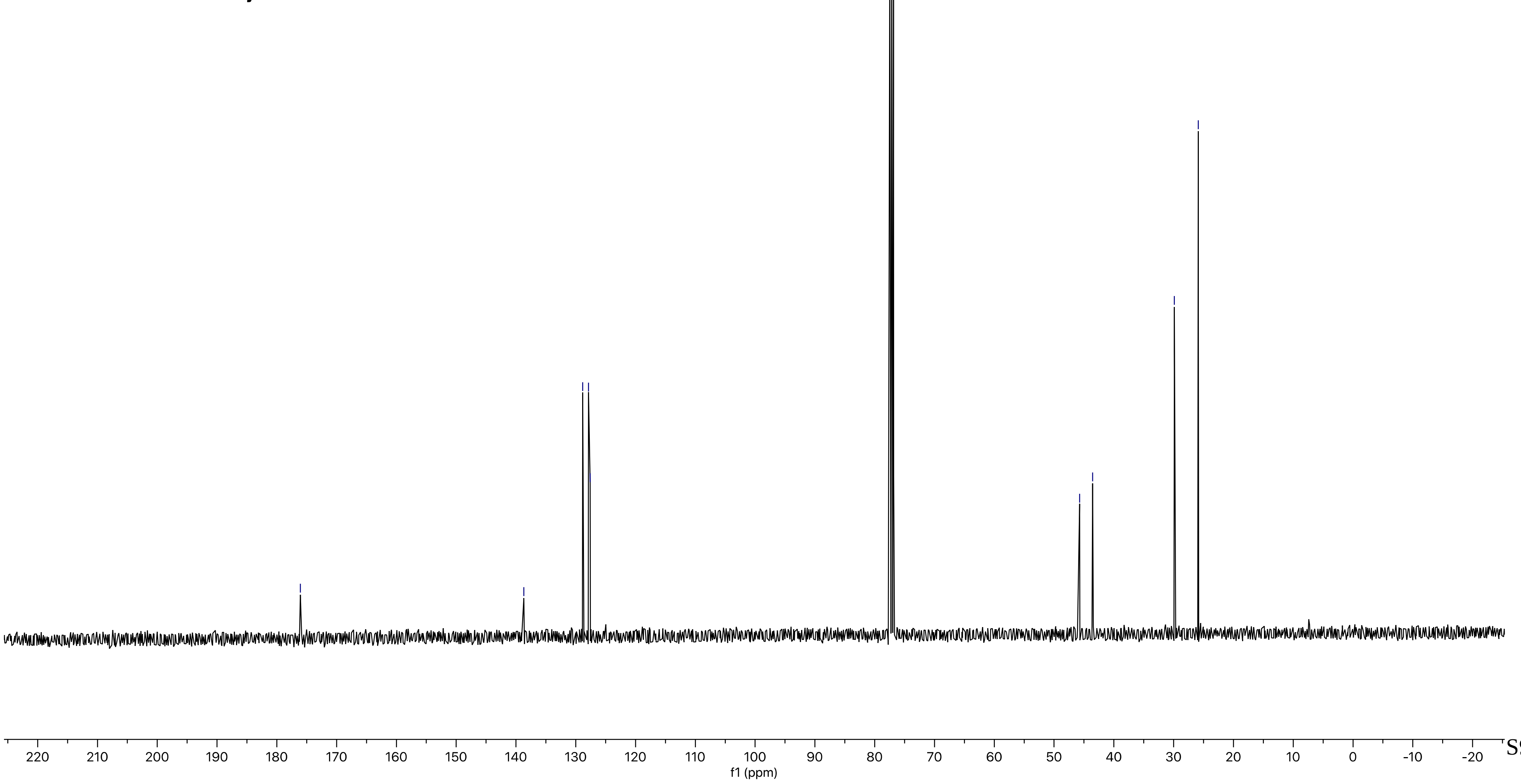

^{1}H NMR: 500 MHz, $CDCl_3$

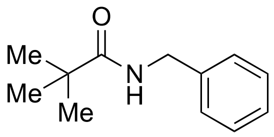

2z

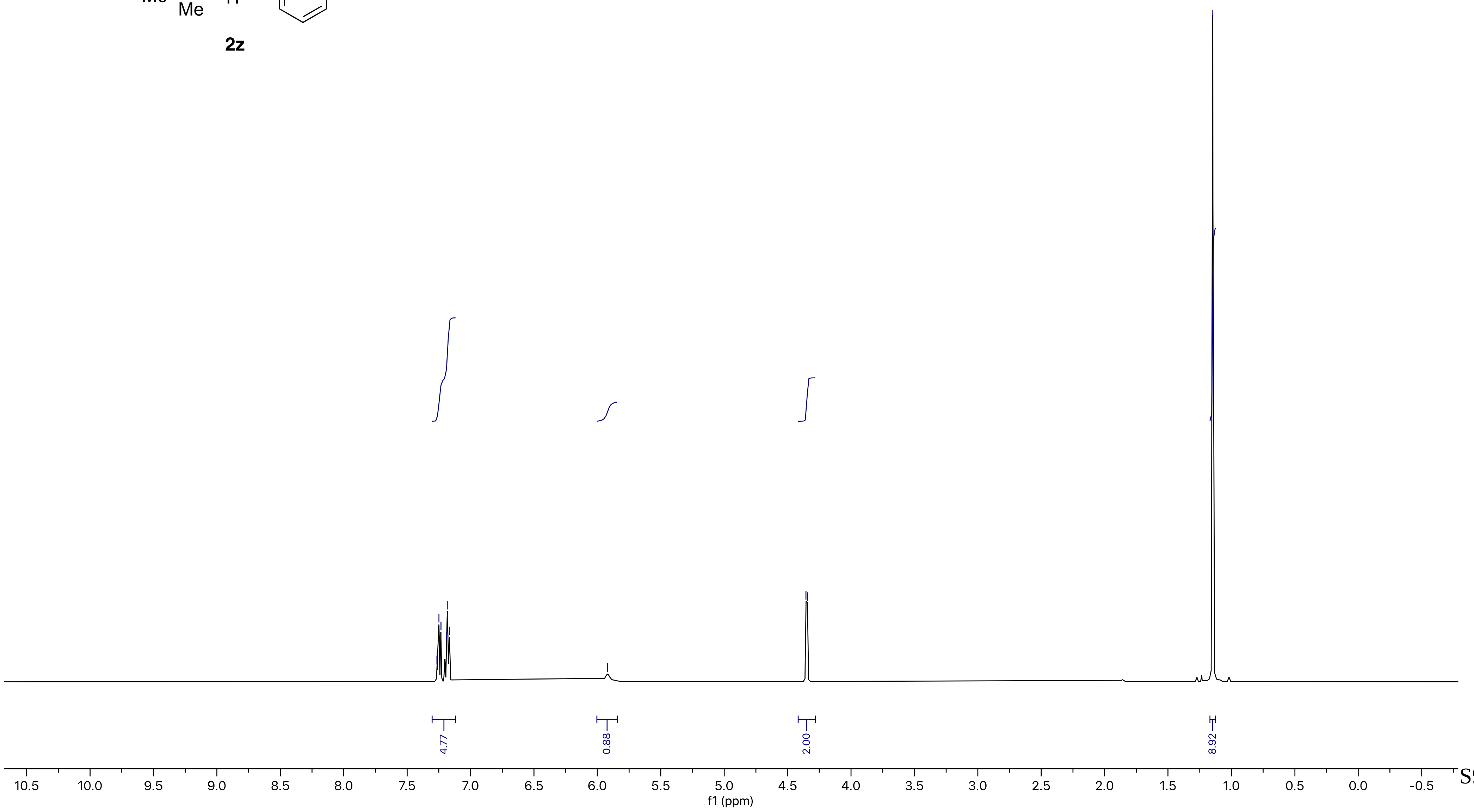

$^{13}C\{^{1}H\}$ NMR: 126 MHz, $CDCl_3$

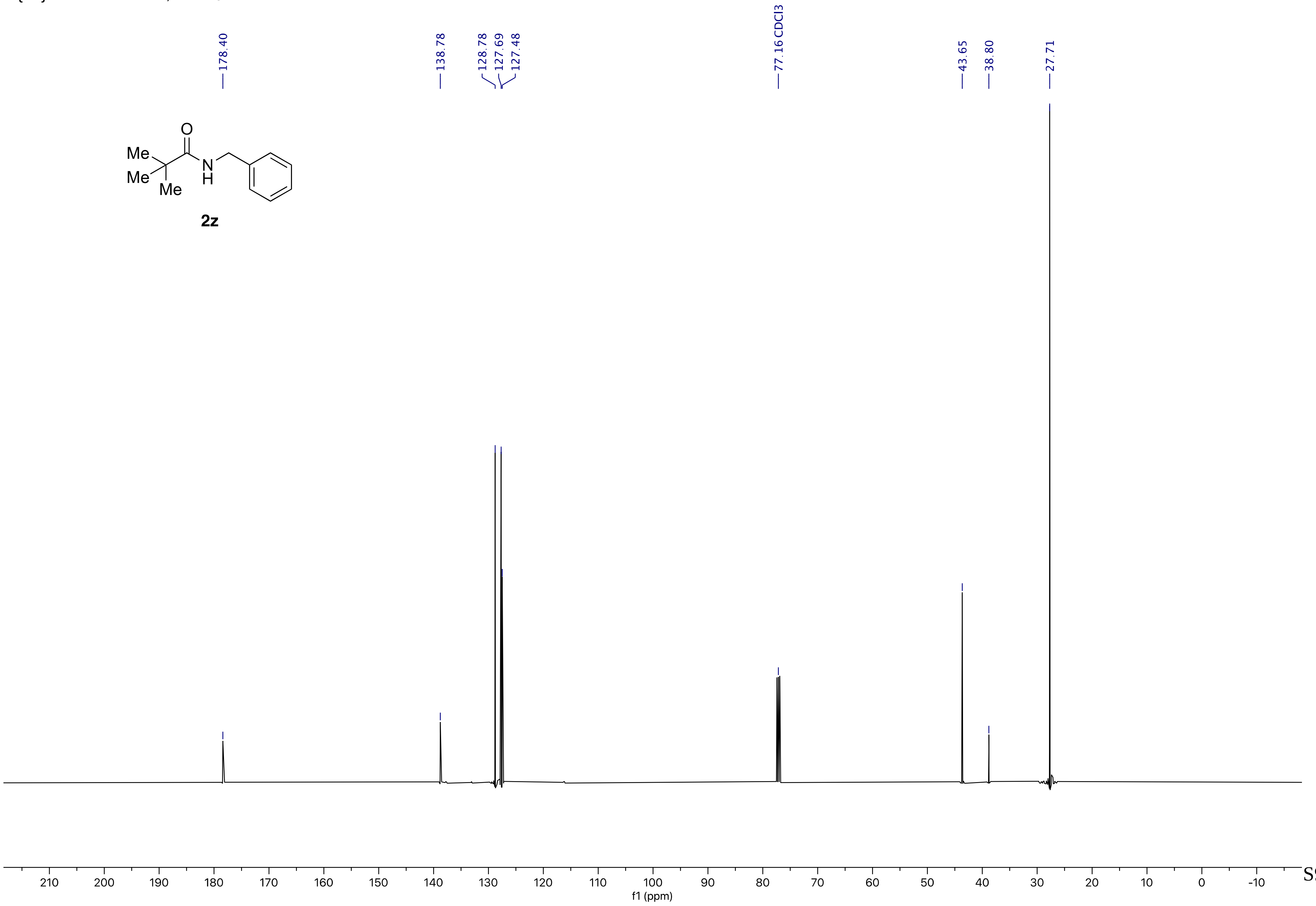

^1H NMR: 500 MHz, $CDCl_3$

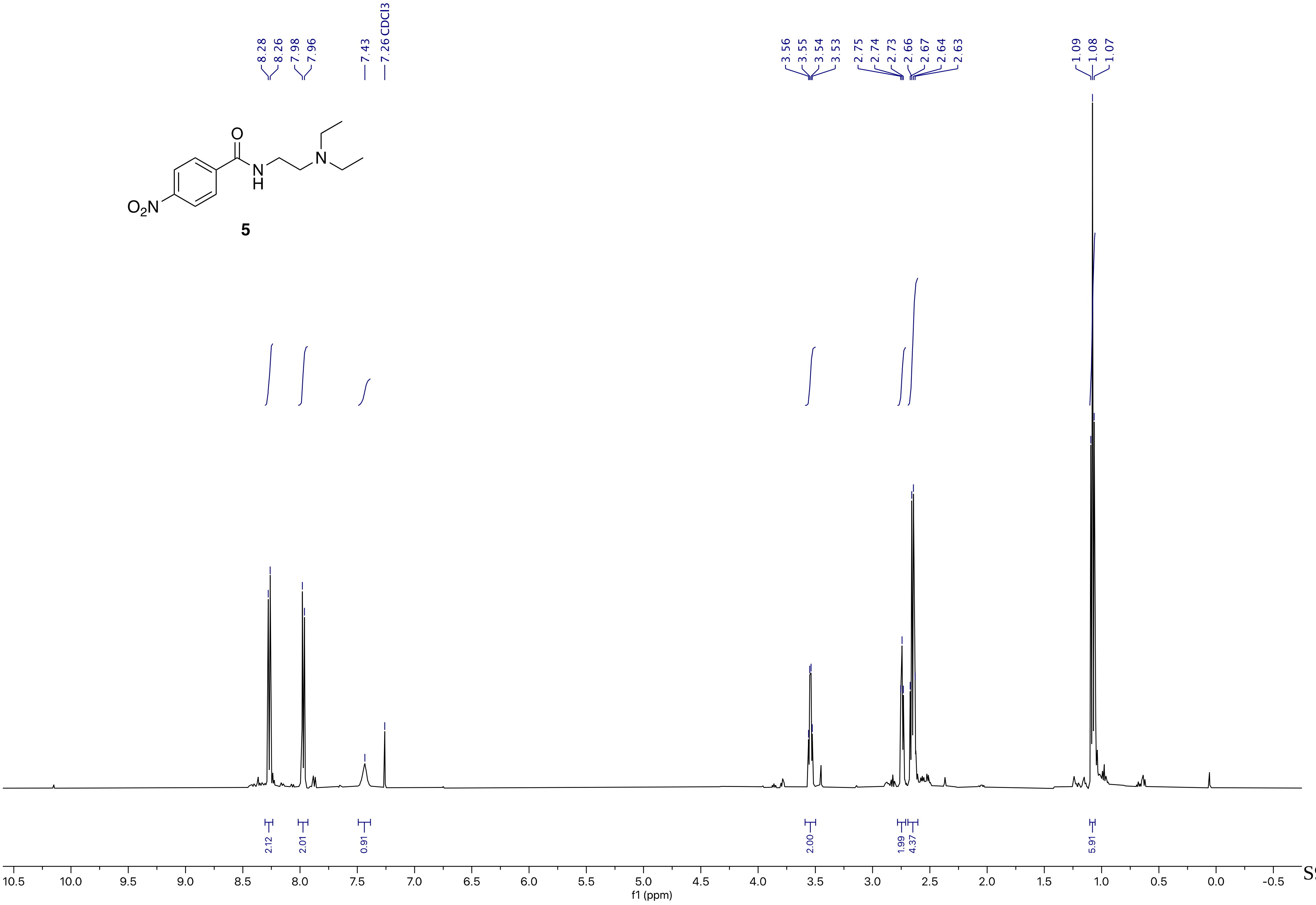

$^{13}C\{^{1}H\}$ NMR: 126 MHz, $CDCl_3$

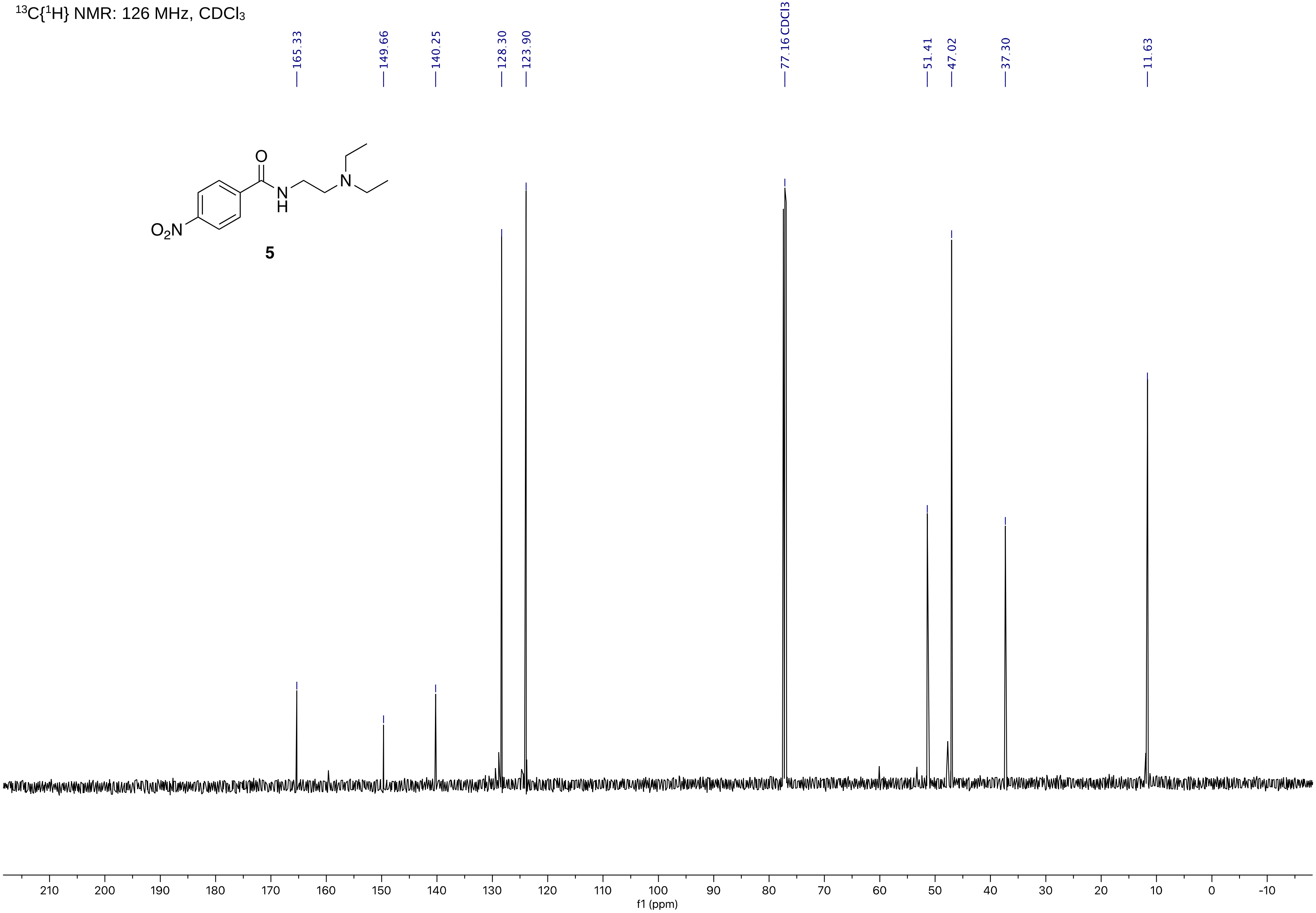

^{1}H NMR: 500 MHz, $CDCl_3$

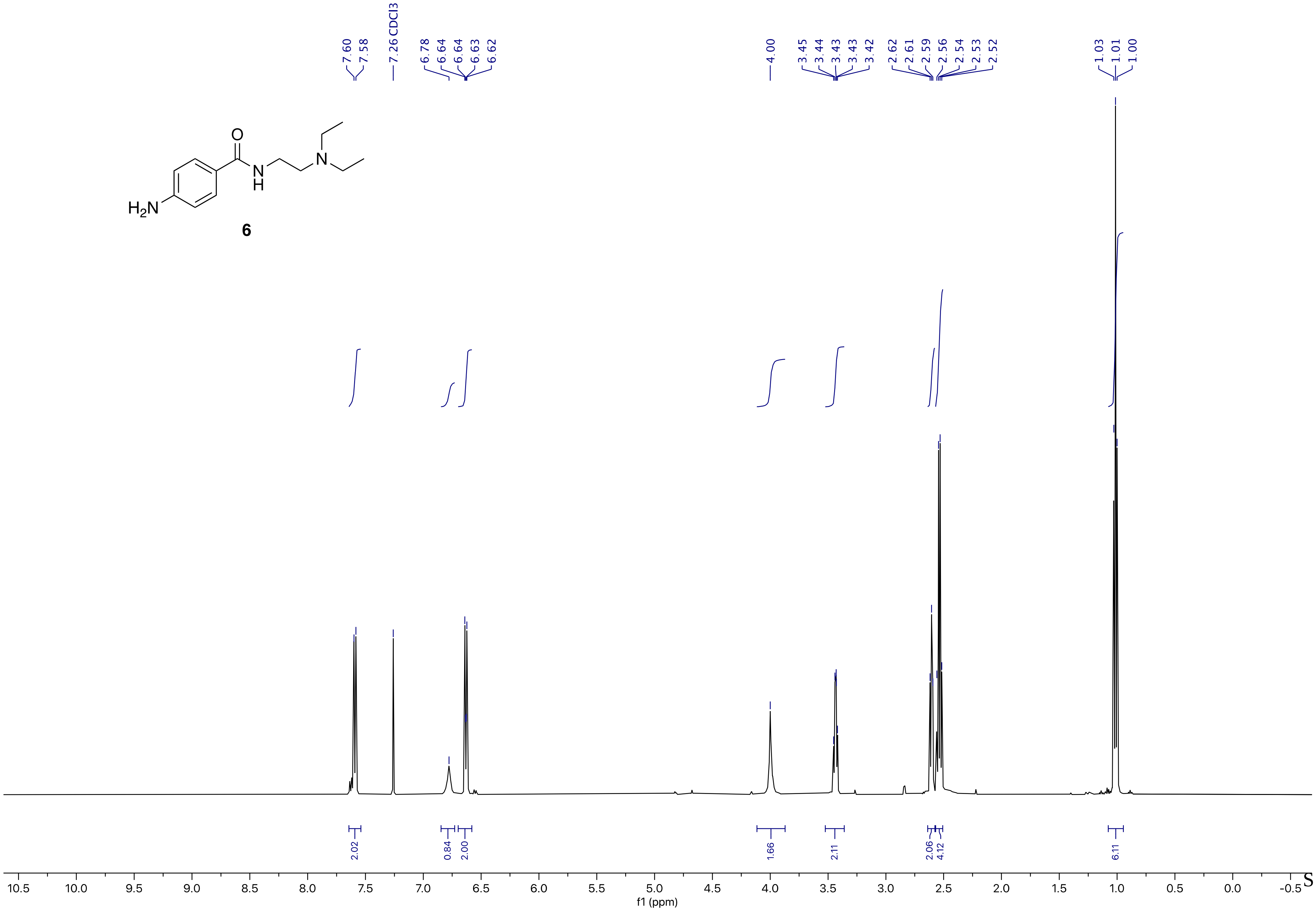

$^{13}C\{^1H\}$ NMR: 126 MHz, $CDCl_3$

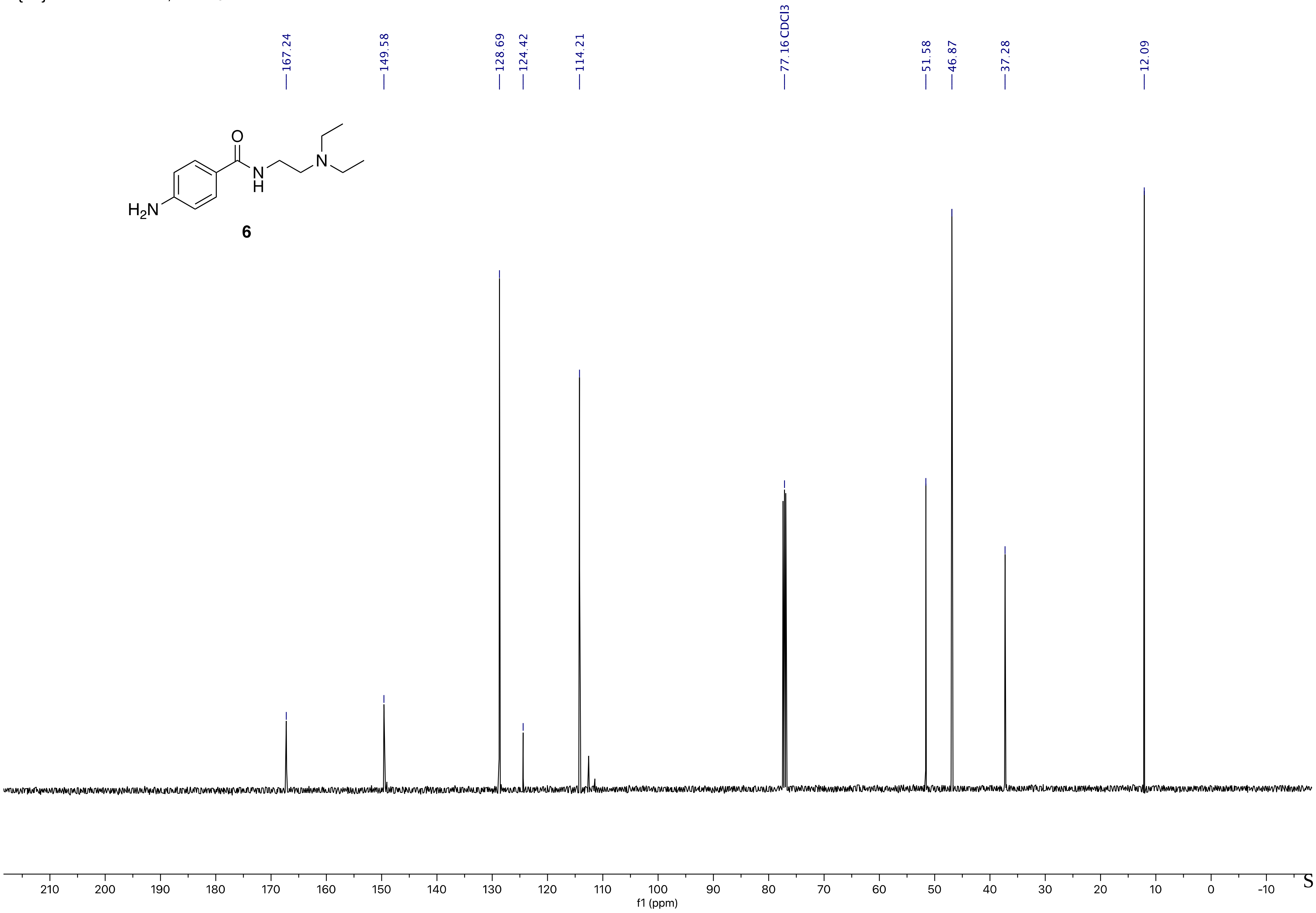

Supplement: Supplementary file 1 — jo4c00575_si_001.pdf [file jo4c00575_si_001.pdf]
